# Exploring Multivalent Architectures for Binding and Stabilization of *N*-Acetylgalactosamine 6-Sulfatase

**DOI:** 10.3390/molecules30102222

**Published:** 2025-05-20

**Authors:** Maria Giulia Davighi, Francesca Clemente, Giampiero D’Adamio, Macarena Martínez-Bailén, Alessio Morano, Andrea Goti, Amelia Morrone, Camilla Matassini, Francesca Cardona

**Affiliations:** 1 Department of Chemistry “U. Schiff” (DICUS), University of Florence, Via della Lastruccia 3-13, 50019 Sesto Fiorentino, Italy; giulia.davighi@gmail.com (M.G.D.); g.dadamio@fabotape.com (G.D.); mmartinez45@us.es (M.M.-B.); alessio.morano@unifi.it (A.M.); andrea.goti@unifi.it (A.G.); 2 Laboratory of Molecular Biology of Neurometabolic Diseases, Neuroscience Department, Meyer Children’s Hospital IRCCS, Viale Pieraccini 24, 50139 Firenze, Italy; amelia.morrone@unifi.it; 3 Department of Neurosciences, Psychology, Drug Research and Child Health University of Florence, Viale Pieraccini 24, 50139 Firenze, Italy; 4 European Laboratory for Non-Linear Spectroscopy (LENS), University of Florence, 50019 Sesto Fiorentino, Italy

**Keywords:** Morquio A, *N*-acetylgalactosamine 6-sulfatase, enzyme stabilizers, iminosugars, dendrimers, multivalent glycomimetics

## Abstract

Morquio A syndrome is a lysosomal disorder caused by the deficiency of the lysosomal enzyme *N*-acetylgalactosamine 6-sulfatase (GALNS, EC 3.1.6.4). Currently, enzyme replacement therapy (ERT) is used to treat Morquio A through the infusion of the recombinant enzyme VIMIZIM^®^ (elosulfase alfa, BioMarin). Unfortunately, the recombinant enzyme exhibits low conformational stability in vivo. A promising approach to address this issue is the coadministration of recombinant human GALNS (rhGALNS) with a pharmacological chaperone (PC), a molecule that selectively binds to the misfolded protein, stabilizes its conformation, and assists in the restoration of the impaired function. We report in this work the synthesis of a library of multivalent glycomimetics exploiting the copper(I)-catalyzed azide-alkyne cycloaddition (CuAAC) reaction between several dendrimeric scaffolds armed with terminal alkynes and azido ending iminosugars of different structures (pyrrolidines, piperidines, and pyrrolizidines) or simple azido ending carbohydrates as bioactive units. The biological evaluation identified pyrrolidine-based nonavalent dendrimers **1** and **36** as the most promising compounds, able both to bind the native enzyme with IC_50_ in the micromolar range and to act as enzyme stabilizers toward rhGALNS in a thermal denaturation study, thus identifying promising compounds for a combined PC/ERT therapy.

## 1. Introduction

Over the past twenty years, multivalency has gained significant importance in biomedicine and bioorganic synthesis due to its fundamental role in various biological phenomena. Although multivalency had been previously extensively studied essentially in carbohydrate–lectin interactions [1,2,3,4,5], its potential in enzyme inhibition started to be explored only since 2009, when iminosugar clusters were identified as potent inhibitors of enzyme Jack bean α-mannosidase [6]. The multivalent effect (MVE) refers to the increased binding affinity exhibited by a multivalent compound, which contains multiple bioactive units attached to a common scaffold, compared with a monovalent counterpart taken as reference. The relative potency (rp) of a multivalent compound is calculated by dividing the IC_50_ (or Ki) value of the monovalent reference compound by the IC_50_ (or Ki) value of the multivalent structure against the target enzyme, and further division by the number of bioactive units (n) quantifies the MVE of a multivalent ligand. It was defined that a positive MVE occurs when this ratio (rp/n) is higher than 1 [7].

Morquio A syndrome (or Mucopolysaccharidosis IVA) is a lysosomal storage disorder caused by the deficiency of the lysosomal enzyme *N*-acetylgalactosamine 6-sulfatase (GALNS, EC 3.1.6.4) that leads to the accumulation of two glycosaminoglycans (GAGs), keratan sulphate and chondroitin 6-sulphate, within lysosomes [8,9,10,11]. The global incidence of Morquio A ranges between 0.009 and 0.14 cases per 10,000 live births. The disease primarily manifests through skeletal abnormalities, connective tissue defects, and cognitive impairments in the more severe forms. Currently, enzyme replacement therapy (ERT) is used to treat Morquio A through the infusion of the recombinant enzyme VIMIZIM^®^ (elosulfase alfa, BioMarin) [12,13], which unfortunately exhibits low conformational stability in vivo and thus limits its effectiveness. One promising approach to improve this treatment is the coadministration of recombinant human GALNS (rhGALNS) with a pharmacological chaperone (PC), a molecule that selectively binds to the misfolded protein, stabilizes its conformation, and assists in the restoration of the impaired function [14,15,16,17,18]. This combined PC/ERT therapy can reduce the required enzyme dosage and administration frequency, thereby minimizing hospitalization and enhancing patients’ quality of life. Successful applications of PC/ERT therapy have been reported for other lysosomal storage disorders [19,20,21,22,23,24].

For this reason, the identification of new compounds that bind rhGALNS is a crucial strategy for the development of enzyme stabilizers.

In addition to the field of lysosomal storage disorders, recent findings associated overexpression of GALNS with cancer [25] and degradation of perineural nets (composed of glycosaminoglycans (GAGs)) around neurons with neuropathic pain [26], thus increasing the interest of the scientific community toward this enzyme.

So far, three non-carbohydrate-based GALNS PCs (bromocriptine, ezetimibe, and pranlukast) [27,28] have been recognized through molecular docking-based virtual screening, and preliminary studies show their ability to increase the activity and thermostability of rhGALNS [29].

The X-ray structure of GALNS reveals a dimeric nature and a certain promiscuity in substrate recognition, with a large open depression on the surface of the enzyme that allows a wide variety of molecules access to the catalytic site [30]. The dimeric nature, in particular, which GALNS shares with other enzymes such as Jack Bean α-mannosidase, α-galactosidase, or β-glucocerebrosidase, has been shown to be prone to accepting multivalent substrates [7,31,32,33,34,35,36].

**Figure 1:**
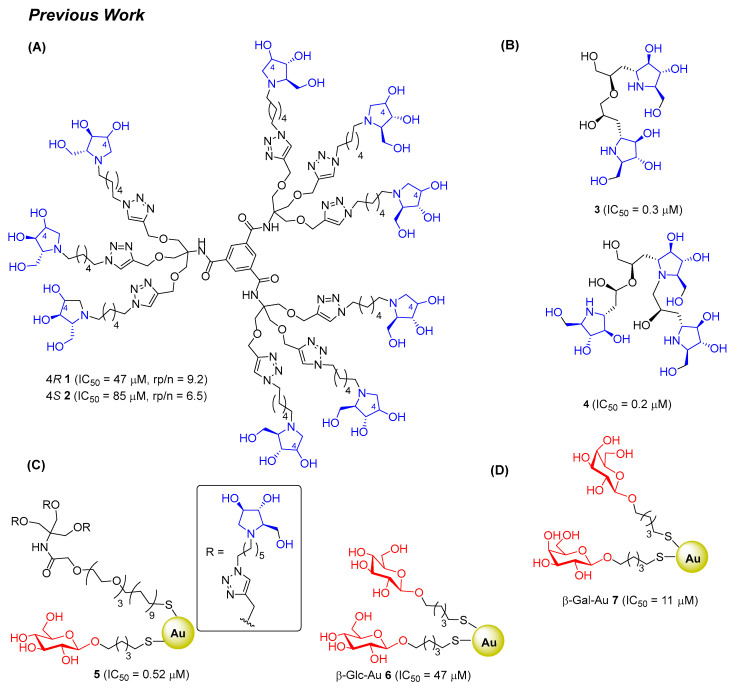
Dendrimers and Au GNPs already known as GALNS inhibitors. (**A**) Nonavalent pyrrolidine architectures. (**B**) Dimer and trimer of DAB-1. (**C**) Glucose-based and mixed glucose-iminosugar gold nanoparticles. (**D**) Monosaccharide-based gold nanoparticles.

In our research group, we previously reported the synthesis of two nonavalent architectures, **1** and **2**, characterized by a rigid aromatic core decorated with the natural 1,4-dideoxy-1,4-imino-d-arabinitol (DAB-1) iminosugar and one of its epimers at C-4, respectively [37]. Compounds **1** and **2** were the first multivalent iminosugar-based GALNS inhibitors identified in the literature. They showed IC_50_ values in the micromolar range (47–85 μM) and a positive MVE (rp/n 6.5–9.2) (Figure 1A).

Afterward, it was found that dimerization and trimerization of DAB-1 obtained through a quite complex nitrone cycloaddition/ring-opening strategy afforded very potent GALNS inhibitors **3** and **4**, with IC_50_ = 0.3 μM and IC_50_ = 0.2 μM, respectively (Figure 1B) [38].

Some of us also reported the first example of gold glyconanoparticles (Au GNPs) functionalized with DAB-1 moiety and 5-(thio)pentyl d-glucopyranoside (GlcC_5_S) that strongly inhibited GALNS in the low micromolar/high nanomolar range (compound **5**, IC_50_ = 0.52 μM). Unexpectedly, the Au GNPs **6**, functionalized with 100% of GlcC_5_S, showed a remarkable inhibition (IC_50_ = 47 μM) toward GALNS, corroborating GALNS promiscuity in substrate recognition and suggesting a synergistic effect of the sugar moiety and/or the scaffold with the iminosugar component in determining the inhibitory activity (Figure 1C) [39]. For this reason, we investigated a library of Au GNPs decorated with various monosaccharides (glucose, mannose, or galactose) with different anomeric configurations (α or β). Among the analyzed compounds, β-Gal-Au GNPs **7** (Figure 1D) was identified as the most effective inhibitor (IC_50_ = 11 μM). Interestingly, this compound was also able to recover enzyme activity after thermal denaturation when tested on the enzyme used for therapy [40].

Considering the above-mentioned results and with the aim of developing new potent and selective binders and stabilizers of the enzyme GALNS, we report herein the synthesis of new dendrimeric multivalent compounds that differ for (i) the bioactive unit (sugar or iminosugar), (ii) the valency, (iii) the linker between the scaffold and the bioactive unit, and (iv) the use of a protected or unprotected bioactive unit. The synthesis of a nonavalent pyrrolidine without hydroxy groups was also undertaken in order to evaluate an eventual synergistic effect of the scaffold in the binding to GALNS.

All synthesized dendrimers were tested as GALNS inhibitors on human leucocyte extracts. The most promising compounds were compared with the appropriate monovalent reference counterpart to quantitatively determine the relative potency (rp) and the multivalent effect (MVE, rp/n). The relative potency with respect to the appropriate corresponding scaffold, which lacked the hydroxy groups, was also evaluated to investigate synergistic effects. For the most promising compounds, the inhibitory activity toward the rhGALNS used for therapy and the ability to impart thermal stabilization to this enzyme were assessed.

## 2. Results and Discussion

We exploited the copper(I)-catalyzed azide-alkyne cycloaddition (CuAAC) reaction as the key synthetic step to obtain the multivalent compounds due to its efficiency and versatility [41,42,43]. CuAAC has proven to be an ultra-fast synthetic procedure, allowing the conjugation of a great number of azide-functionalized moieties onto a single alkynyl-armed multivalent scaffold with high yields and reduced reaction times.

Based on our previous experience in the synthesis of multivalent iminosugars and given the polarity of our azide components, the reaction conditions for the CuAAC that we employed in this study were the following: catalytic CuSO_4_ (0.3 equiv), sodium ascorbate (0.6 equiv), a 2:1 THF:H_2_O mixture as the solvent, microwave irradiation (MW 80 °C) for 45 min time, followed by flash column chromatography (FCC) or size exclusion chromatography (SEC) and eventual treatment with copper scavenger resins [44].

**Figure 2:**
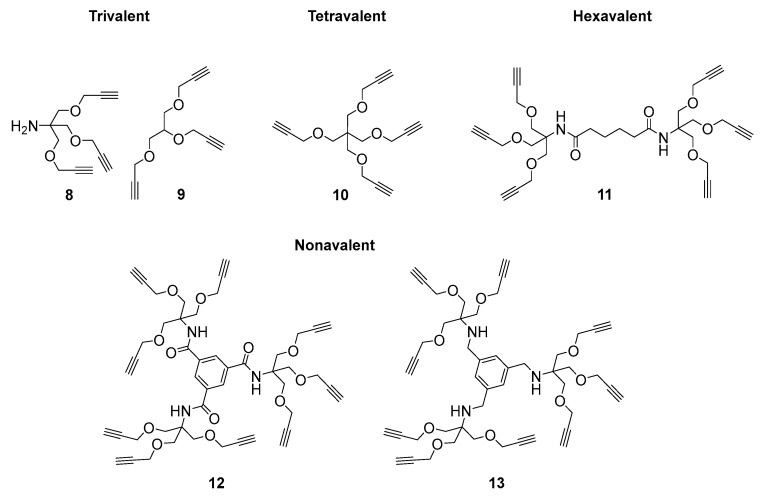
Alkynyl-armed multivalent scaffold used in the CuAAC.

A series of scaffolds with different geometries and numbers of alkyne moieties, from trivalent and tetravalent to hexavalent and nonavalent, were selected (Figure 2). The trivalent and tetravalent **8** [45,46], **9** [47], and **10** [48] were obtained by propargylation of the corresponding alcohols (tris(hydroxymethyl)aminomethane, glycerol, and pentaerythritol), while the dendrimeric hexavalent and nonavalent **11** [49] and **12** [50] were prepared by coupling adipic acid and trimesoyl chloride, respectively, with tris[(propargyloxy)methyl]aminomethane **8**. The new more basic and flexible scaffold **13** was also investigated in this study.

Concerning the synthesis of iminosugar-derived dendrimers, the initial attempts of CuAAC were successfully performed on the alkynyl-armed tetravalent **10** with the benzylated DAB-1 analog **14** [51] bearing a 6-azido hexane linker at the nitrogen atom. However, as revealed in our previous works, significant challenges arose during the deprotection of the perbenzylated adducts and purification of the crude reaction due to the high basicity and hydrophilicity of the polyhydroxylated compounds [51,52].

**Scheme 1:**
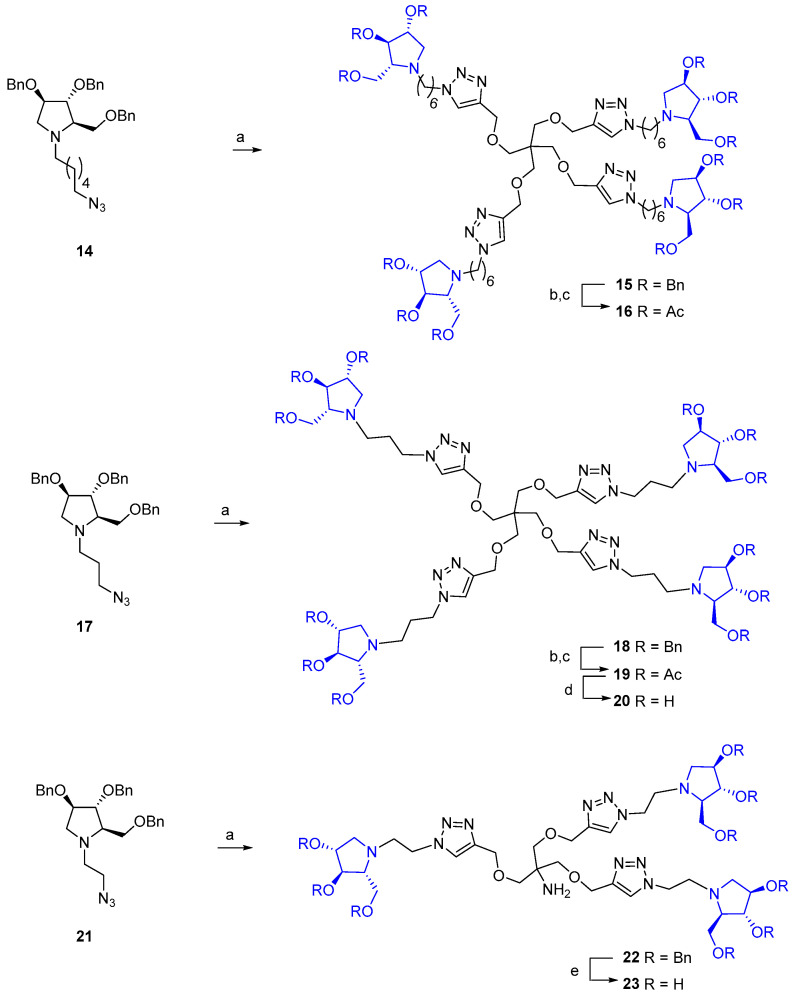
(a) **10**, CuSO_4_, sodium ascorbate, THF:H_2_O 2:1, MW 80 °C, 45 min, 96% for **15** (Ref. [51]), 86% for **18,** 68% for **22** (Ref. [55]). (b) H_2_, Pd/C, MeOH, HCl, r.t., 3 d. (c) Ac_2_O, pyridine, r.t., 16 h, 49% over 2 steps for **16**, 38% over 2 steps for **19**. (d) Ambersep 900 OH, MeOH, r.t., 16 h, 95%. (e) BCl_3_ 1M in hexane, CH_2_Cl_2_, r.t., 18 h, then Ambersep 900 OH, EtOH, r.t., 30 min, quantitative.

Purification could be achieved after per *O*-acetylation of the hydroxy groups after the debenzylation by catalytic hydrogenation to better characterize the final multivalent architectures, which gave us the opportunity to test also the peracetylated adducts (Scheme 1). The choice of the acetyls as the protecting group for the hydroxyls was motivated by the observation that peracetylated compounds can act as pro-drugs when tested on cells by facilitating cellular uptake, as previously reported by Compain and co-workers [53]. Catalytic hydrogenation of **15** [51] with Pd/C in acidic MeOH for 3 days followed by treatment with an excess of acetic anhydride and pyridine at room temperature for 16 h furnished the compound **16** in 49% yield over two steps (Scheme 1). To investigate the influence of a shorter linker between the polyhydroxypyrrolidines and the branch point of the multivalent architecture on GALNS inhibition, we selected the tetravalent scaffold **10** as it is easier to prepare compared with the other alkynes, along with the polyhydroxylated azido intermediate **17** bearing a 3-carbon-atom chain (Scheme 1). The CuAAC reaction of **10** with **17**, carried out under the previously described conditions, afforded a product that could be used in the next step without further purification. The tetravalent pyrrolidine compound **18** was isolated in 86% of yield after FCC. The deprotection of the perbenzylated adduct **18** by catalytic hydrogenation in acidic MeOH, followed by acetylation of the crude with an excess of acetic anhydride in pyridine, provided peracetylated compound **19** in 38% yield over two steps. The final treatment of **19** with the strongly basic resin Ambersep 900 OH in MeOH for 16 h gave the pure polyhydroxylated multivalent iminosugar **20** in 95% yield (Scheme 1). We then synthesized the trivalent compound **23**, which contained a free amine group on the scaffold and featured three pyrrolidines separated from the triazole moieties by a two-carbon aliphatic chain starting from azido-compound **21** (Scheme 1). To avoid undesired *N*-acetylation during the final acetylation step, the debenzylation procedure was modified by employing an acidic treatment with BCl_3_, followed by a basic workup [54]. The reaction of **22** [55] with BCl_3_ in CH_2_Cl_2_ at room temperature for 18 h followed by quenching with EtOH and treatment with the strongly basic Ambersep 900 OH resin for 30 min furnished **23** in 96% yield after filtration and purification over Sephadex LH-20 resin (Merk Life Science S.r.l., Merck KGaA, Germany) (Scheme 1).

**Scheme 2:**
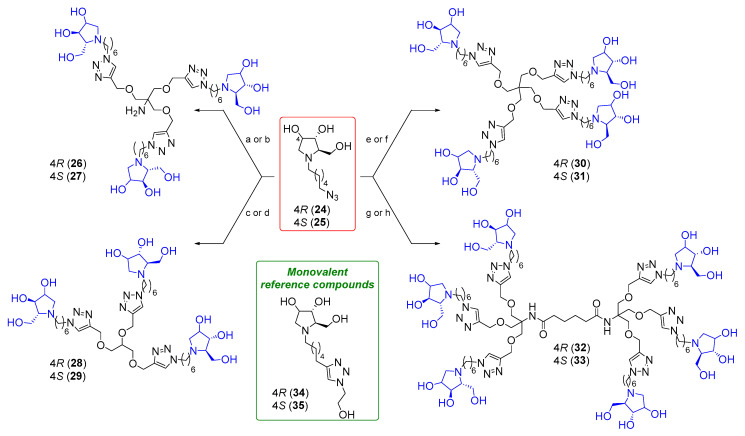
(a) From **24**, reaction with **8**, CuSO_4,_ sodium ascorbate, THF:H_2_O 2:1, MW 80 °C, 45 min, 76% of **26** (Ref. [51]). (b) From **25**, reaction with **8**, CuSO_4,_ sodium ascorbate, THF:H_2_O 2:1, MW 80 °C, 45 min, 62% of **27**. (c) From **24**, reaction with **9**, CuSO_4,_ sodium ascorbate, THF:H_2_O 2:1, MW 80 °C, 45 min, 78% of **28**. (d) From **25**, reaction with **9**, CuSO_4,_ sodium ascorbate, THF:H_2_O 2:1, MW 80 °C, 45 min, 67% of **29**. (e) From **24**, reaction with **10**, CuSO_4,_ sodium ascorbate, THF:H_2_O 2:1, MW 80 °C, 45 min, 48% of **30** (Ref. [51]). (f) From **25**, reaction with **10**, CuSO_4,_ sodium ascorbate, THF:H_2_O 2:1, MW 80 °C, 45 min, 73% of **31**. (g) From **24**, reaction with **11**, CuSO_4,_ sodium ascorbate, THF:H_2_O 2:1, MW 80 °C, 1 h 30 min, 73% of **32**. (h) From **25**, reaction with **11**, CuSO_4,_ sodium ascorbate, THF:H_2_O 2:1, MW 80 °C, 45 min, 72% of **33**.

To reduce the overall number of synthetic steps, we chose to employ the polyhydroxylated azido intermediate **24** instead of the benzylated derivative **14** in the CuAAC reactions depicted in Scheme 2.

Copper-catalyzed cycloaddition of the deprotected azide **24** was run with the trivalent **9** and the hexavalent **11** in the conditions described above. After treatment with the copper scavenger resin Quadrasil MP^®^ (Merk Life Science S.r.l., Merck KGaA, Germany) and purification by flash column chromatography (FCC) or size exclusion chromatography (SEC), the final multivalent iminosugars **28** and **32** were afforded in 78% and 73% yields, respectively (Scheme 2).

Analogous multivalent architectures **26** and **30** were previously reported by our research group with good to fair yields (76% and 48%, respectively) following the same synthetic strategy and tested against different types of α-mannosidases (Jack bean α-mannosidase (JBMan), Golgi α-mannosidase (GMII), and lysosomal α-mannosidase (bLAM)) [51]. To investigate the role of different C-4 configurations on the biological activity, the epimeric azide **25** underwent CuAAC reactions with the alkynyl-armed multivalent scaffolds **8**–**12** under the previously described conditions, leading to the synthesis of polyhydroxylated multivalent iminosugars **27**, **29**, **31**, and **33**.

After treatment with the copper scavenger resin Quadrasil MP^®^ and purification upon FCC and/or by SEC, the polyhydroxylated compounds **27**, **29**, **31**, and **33** were obtained in 62–73% yields (Scheme 2). Derivatives **34** and **35**, which were previously synthesized by our research group, were used as monovalent reference compounds [37].

**Scheme 3:**
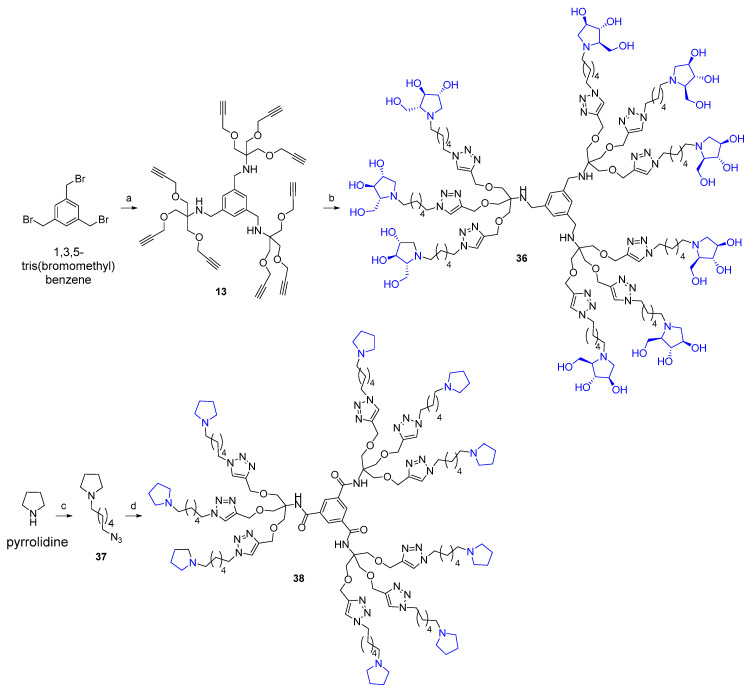
(a) **8**, K_2_CO_3_, CH_3_CN, r.t., 18 h, 68%. (b) **24**, CuSO_4,_ sodium ascorbate, THF:H_2_O 2:1, MW 80 °C, 45 min, 82%. (c) 1-azido-6-bromohexane, K_2_CO_3_, CH_3_CN:H_2_O 5:1, MW 120 °C, 3 h, 36%. (d) **12**, CuSO_4,_ sodium ascorbate, THF:H_2_O 2:1, MW 80 °C, 45 min, 90%.

Given the promising inhibition data for the nonavalent architectures **1** and **2** [37], we synthesized two new nonavalent derivatives introducing some structural modifications to investigate their potential impact on the biological activity against the GALNS enzyme. First, we modified the scaffold carrying out the synthesis of the more basic and flexible compound **36**, an analog of **1** that shows a stronger positive multivalent effect. In compound **36**, the three amide bonds were replaced with amine moieties, while retaining the 4*R* configuration of the iminosugar ring (Scheme 3), which gave the best results (see Table 1, compound **1** vs. **2**).

Starting from 1,3,5-tris(bromomethyl)benzene, the substitution of the bromine groups with the amine **8** in the presence of K_2_CO_3_ in CH_3_CN at room temperature for 18 h afforded the alkynyl-armed nonavalent scaffold **13** in 68% yield.

The CuAAC reaction of **13** with the deprotected (4*R*) azide **24** in the conditions described above yielded the nonavalent compound **36** (82%) after treatment with the copper scavenger resin Quadrasil MP^®^ and purification over Sephadex LH-20 resin (Scheme 3).

In addition, we synthesized a reference nonavalent compound, maintaining **12** as the scaffold but featured with simple pyrrolidines as inhitopes, in order to assess the role of polyhydroxyl groups and to examine the eventual synergistic role of the scaffold itself on the binding affinity toward GALNS enzyme. To our knowledge, the role of the scaffold has not been investigated so far in relation to multivalent iminosugars. The reaction of pyrrolidine with 1-azido-6-bromohexane [56] in the presence of K_2_CO_3_ in a 5:1 CH_3_CN:H_2_O mixture under MW irradiation at 120 °C for 3 h resulted in the alkylated compound **37** in 36% yield (Scheme 3). Hence, **37** was employed in the CuAAC reaction with the nonapropargylated scaffold **12** in the usual conditions described above to yield the compound **38** in 90% yield after treatment with QuadraSil^®^ MP resin, followed by size exclusion chromatography (Sephadex LH-20 resin) (Scheme 3).

**Scheme 4:**
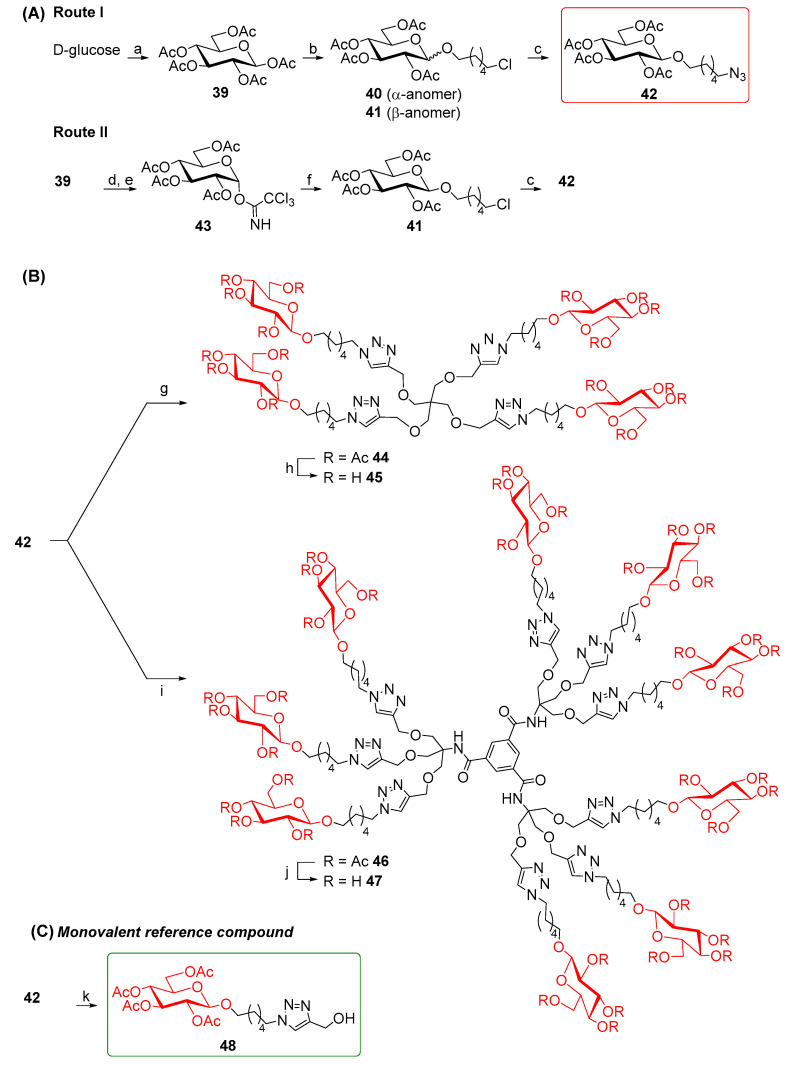
(**A**) Synthesis of glucose derivative **42.** (**B**) Synthesis of multivalent glucose derivatives. (**C**) Synthesis of the monovalent reference compound **48.** (a) Ac_2_O, NaOAc, reflux to r.t., 74%. (b) 6-chlorohexanol, BF_3_·OEt_2_, 3Å MS, dry CH_2_Cl_2_, 0 °C to r.t., 26 h, 25% (mixture of α and β anomers). (c) **41** (β-anomer), NaN_3_, dry DMF, 50 °C, 4 days, 79%. (d) 1,2-diamino ethane, AcOH, dry THF, r.t., 7 h, 86%. (e) Cl_3_CCN, DBU, dry CH_2_Cl_2_, 0 °C to r.t., 5 h, 65%. (f) 6-chlorohexanol, BF_3_·OEt_2_, 3Å MS, dry CH_2_Cl_2_,—78 °C to r.t., 18 h, 58%. (g) **10**, CuSO_4_, sodium ascorbate, THF:H_2_O 2:1, MW 80 °C, 45 min, 79%. (h) Ambersep 900 OH, MeOH, r.t., 18 h, 88%. (i) **12**, CuSO_4_, sodium ascorbate, THF:H_2_O 2:1, MW 80 °C, 45 min, 91%. (j) Na_2_CO_3_, MeOH, r.t., 18 h, 61%. (k) propargyl alcohol, CuSO_4_, sodium ascorbate, THF:H_2_O 2:1, r.t., 40 h, 61%.

Since gold glyconanoparticles (Au GNPs) functionalized with sugar moieties (β-glucose and β-galactose) exhibited excellent GALNS inhibition [39,40], we synthesized new dendrimers decorated with β-glucose units, in both their acetylated and polyhydroxylated forms, to further investigate the role of the sugar component in the inhibition by multivalent compounds. To synthesize the sugar-derived **42** functionalized on the anomeric position with a six-carbon-atom chain ending with an azido moiety, we followed two synthetic routes starting from d-glucose. The azide **42** was first obtained by directly performing the glycosylation reaction on β-d-glucose pentaacetate **39** with 6-chlorohexanol, BF_3_·OEt_2_, and 3 Å molecular sieves in dry CH_2_Cl_2_, yielding both the α- and β-anomers **40** and **41** (Route I, Scheme 4A) in a 25% overall yield. The β:α anomer ratio was 1.17:1. The β-anomer **41** was subsequently treated with NaN_3_ in DMF at 50 °C for four days to give the azide **42** [57,58] in 79% yield. Alternatively, trichloroacetimidate **43** [59] underwent a glycosylation reaction with 6-chlorohexanol in the presence of BF_3_·OEt_2_ as Lewis acid and 3Å molecular sieves in dry CH_2_Cl_2_ affording selectively the corresponding β-anomer **41** in 58% yield. Compound **41** was then converted into the azide **42** (Route II, Scheme 4A). The overall yield of Route II was 19%. Although the first synthetic strategy (Route I) resulted in a slightly lower overall yield (8%) compared with Route II, it required fewer steps (Scheme 4A). In addition, Route I produced a mixture of two diastereoisomers (**40** and **41**), which complicated the purification of the intermediate step. Despite requiring more steps, Route II proceeded with a higher overall yield (19%), nearly double that of Route I. Given the substantially higher yield, Route II was the more favorable route as maximizing the yield of product **42** was a priority in this case.

The CuAAC reaction of the azido derivative **42** with the tetravalent scaffold **10** was carried out as usual to afford the acetylated tetravalent derivative **44** in 79% yield, after flash column chromatography (Scheme 4B). Deprotection of acetyl groups of **44** using the strongly basic resin Ambersep 900 OH in MeOH followed by purification over the size exclusion Bio-Beads SX-8 resin (Merk Life Science S.r.l., Merck KGaA, Germany) furnished the tetravalent dendrimer **45** in 88% yield. The CuAAC reaction of the nonavalent **12** and the sugar-derived azide **42** gave the acetylated nonavalent compound **46** in 91% yield after purification by FCC (Scheme 4B).

We faced some challenges in the cleavage of the acetyl groups of compound **46**. The main attempts for the basic deprotection at room temperature are reported in the Supporting Information. Finally, the treatment of **46** with Na_2_CO_3_ for 18 h, followed by neutralization of the reaction mixture with 1% HCl in MeOH and subsequent filtration, afforded **47** in 61% yield. To evaluate the relative inhibitory activity enhancement of these newly derived sugar multivalent architectures, a corresponding monovalent counterpart was also synthesized. Starting from the azido derivative **42**, a CuAAC reaction was performed with propargyl alcohol in the presence of CuSO_4_ and sodium ascorbate in THF:H_2_O 2:1 mixture at room temperature for 40 h, yielding the adduct **48** in 61% yield (Scheme 4C).

**Scheme 5:**
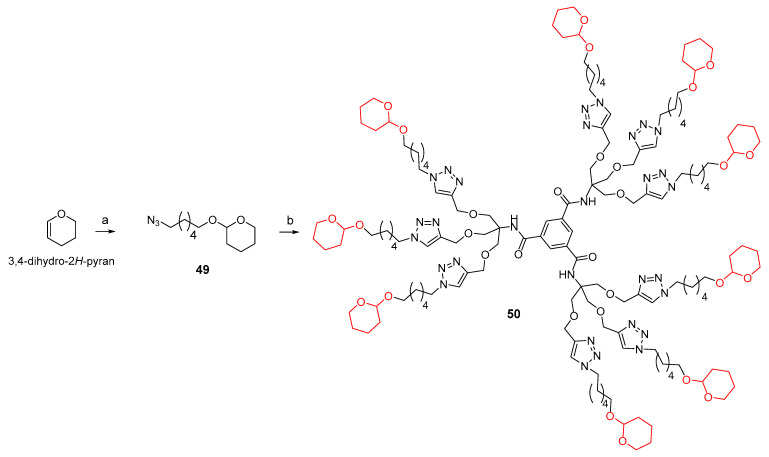
(a) 6-azidohexan-1-ol, Amberlyst 15, Hexane:Et_2_O 1:1, r.t., 3h, 89%. (b) **12**, CuSO_4,_ sodium ascorbate, THF:H_2_O 2:1, MW 80 °C, 45 min, 46%.

In this case as well, we synthesized a reference nonavalent compound, using **12** as the scaffold while incorporating tetrahydro-2*H*-pyran as the inhitope. This design aimed to evaluate the role of polyhydroxyl groups and investigate any potential synergistic effect of the scaffold itself on binding affinity toward the GALNS enzyme. The reaction of 3,4-dihydro-2H-pyran with azidohexan-1-ol [60] in the presence of Amberlyst 15 in a 1:1 Hexane:Et_2_O mixture at room temperature for 3 h yielded the acetal **49** with an 89% yield (Scheme 5). Subsequently, **49** underwent a CuAAC reaction with the nonapropargylated scaffold **12** under the previously described conditions, affording the desired product **50** in a 46% yield after treatment with QuadraSil^®^ MP resin, followed by flash column chromatography (Scheme 5).

## 3. Biological Screening

A preliminary biological screening of the newly synthesized multivalent compounds at 1 mM inhibitor concentration was performed against GALNS in leukocytes isolated from healthy donors. Enzymatic assays were performed in triplicate by incubating leukocyte homogenate, compound, and substrate (4-methylumbelliferyl-β-galactoside-6-sulfate.Na) in Na-acetate/acetic acid buffer (pH 4.3) at 37 °C for 17 h, followed by the addition of β-galactosidase from *Aspergillus oryzae* and further incubation to release 4-methylumbelliferone. The fluorescence was measured, and the inhibition percentages were calculated relative to the control. The results are summarized in Table 1, Table 2, Table 3 and Table 4, along with new inhibition data toward GALNS of multivalent inhibitors whose synthesis was reported previously by our group (**23**, **26**, **30**, **51**, **52–54**). For compounds showing poor to negligible inhibitory activity, only the GALNS inhibition percentage at 1 mM was reported.

Conversely, IC_50_ values were calculated by fitting hydrolysis rates to a dose–response model using Origin for the best GALNS inhibitors (i.e., GALNS inhibition at 1 mM ≥ 75%), as well as for those compounds for which a numerical value was necessary to compare multivalent ligands with reference compounds (i.e., compounds **34**, **35**, and **38**; Table 1 entries 1, 2, and 6). The only exception was represented by compound **33** (Table 2), but given the poor result (IC_50_ = 350 μM), we did not proceed further. Monovalent reference compounds **34** and **35** showed negligible inhibition against GALNS (IC_50_ = 3900 μM and 5000 μM, respectively; Table 1, entries 1, 2 [37]) and were used as monovalent reference compounds to evaluate the MVE for the multivalent derivatives.

In general, as previously reported in the literature for **1** and **2** [37], the nonavalent architectures were found to be most effective in inhibiting the GALNS enzyme, with rp/n values >1. Better results were obtained for the d-arabinose-configured compound (**1**) with respect to the d-ribose-configured one (**2**) (Table 1, entry 3 vs. entry 4). The GALNS preference for the d-arabinose vs. the d-ribose configuration was evident also considering the d-ribose-configured hexavalent compound **33**, for which a little rp/n = 2.4 was calculated (Table 2).

Interestingly, the newly synthesized d-arabinose-configured **36** exhibited a very high inhibition value (Table 1, entry 5, 93%) at 1 mM with an IC_50_ in the low micromolar range (IC_50_ = 28 µM). Therefore, the replacement of the amide bonds in **1** with amine bonds in **36** almost doubled the inhibitory activity and showed an enhanced MVE (rp/n = 15.4 for **36** vs. rp/n = 9.2 for **1**; Table 1, entry 5 vs. 3). We can only envisage, at this stage, that the improved inhibitory value with compound **36** was due to the higher basicity of the scaffold or its higher flexibility.

Conversely, the IC_50_ measured for the nonavalent compound **38** featured with simple pyrrolidines as inhitopes (IC_50_ = 70 μM; Table 1, entry 6) showed that the nonavalent scaffold itself played a key role in determining GALNS inhibition, even without the hydroxy groups, suggesting a synergistic effect between the iminosugar and the multivalent scaffold in imparting the inhibitory activity.

Taking **38** as a multivalent reference compound and by evaluating the relative potency inhibition of **1** with respect to **38**, we calculated an rp = 1.5, which demonstrated that the presence of the iminosugars was still relevant to improve inhibition (Table 1, entry 3).

Instead, comparing the inhibitory activity of the d-ribose-configured **2** with respect to **38** resulted in no advantage resulting from the presence of the iminosugar (Table 1, entry 4 vs. 6), thus corroborating the preference of GALNS for the d-arabinose configuration.

Decreasing the valency of the multivalent compounds was not beneficial for the inhibitory properties toward GALNS. Indeed, the trivalent compounds **28**, **29**, **26 [51]**, **27**, and **23** [55] derived from glycerol or from the tris[(propargyloxy)methyl]aminomethane amine **8** exhibited inhibition percentages lower than 75% (Table 2). Again, among the trivalent compounds with a six-carbon-atom linker **28**, **29**, **26**, and **27**, we observed that the d-arabinose-derived compounds **28** and **26** (with inhibition rates of 63% and 73% at 1 mM, respectively; Table 2) were preferred over the d-ribose-derived **29** and **27** (with inhibition rates of 18% and 46% at 1 mM, respectively; Table 2). This confirmed that changes in the absolute configuration at C-4 played a role in determining their affinity for GALNS. Coupling the free amine of **26** with 4-nitrobenzoyl chloride to afford the less polar derivative **51** [55] resulted in lower GALNS inhibition (49% for **51** vs. 73% for **26**; Table 2). Similarly, reducing the linker between the polyhydroxy pyrrolidines and the triazole moieties (35% for **23** vs. 73% for **26**; Table 2) resulted in lower inhibition. In analogy to the trivalent derivatives, also the tetravalent compounds resulted poor inhibitors. However, an opposite trend was observed with the d-ribose-configured **31** being twice as potent as the d-arabinose-derived **30** (inhibition of 73% for **31** vs. 35% for **30**; Table 2). The acetylation of the hydroxy groups in **30** to yield compound **16** did not significantly improve the inhibition properties toward GALNS (35% for **30** vs. 47% for **16**; Table 2). Similarly, the acetylated **19** with the shorter linker between the trihydroxy pyrrolidines and the triazole moieties exhibited a very poor 25% GALNS inhibition (Table 2).

Both the d-arabinose and d-ribose hexavalent **32** and **33** showed moderate inhibition properties (66% and 72% at 1 mM, respectively; Table 2) against GALNS. For the d-ribose-derived **33**, an IC_50_ of 350 ± 80 μM was determined, which allowed the calculation of a low but positive multivalent effect rp/n of 2.4 when compared with its monovalent counterpart **35** (Table 1).

We then considered other multivalent compounds present in house, containing either pyrrolizidine or piperidine iminosugars as bioactive inhitopes.

The multivalent bicyclic pyrrolizidines [44] (compounds **52**, **53**, and **54**; Table 2) did not overall improve GALNS inhibition compared with the corresponding multivalent pyrrolidine-armed compounds. Indeed, almost negligible inhibition was found for the tetravalent **54** (9% at 1 mM; Table 2), indicating again that a short linker between trihydroxy pyrrolidine and the triazole moiety (in analogy to compound **23**; Table 2) had a detrimental effect on the inhibitory activity.

The longer linker in the trivalent compound **52** led to a mild improvement in its inhibitory activity (52% at 1 mM; Table 2). Increasing the number of iminosugar units and considering the same aromatic scaffold that yielded good results with the pyrrolidine series as in the nonavalent **53** led to a 63% inhibitory activity (Table 2). Considering these data and the more complicated and longer synthesis of pyrrolizidine iminosugars compared with the pyrrolidine derivatives [44], we deemed that it was not worthwhile to continue studying the GALNS inhibition properties with pyrrolizidine multivalent derivatives.

Conversely, the analysis of the previously synthesized monovalent and multivalent piperidine derivatives **55** [61], **56** [62], **57** [62], and **58** [52] (Table 3) revealed that all these compounds, regardless of the valency, showed inhibitory percentages no higher than 50% and even lower than that of the monovalent compound **55** taken as reference (57%; Table 3).

Among the newly synthesized glucose-derived dendrimers, the acetyl-protected dendrimers **44** and **46** showed good inhibitory activity against GALNS (66% and 78% inhibition, respectively; Table 4, entries 1 and 3), with again the nonavalent **46** (Table 4, entry 3) being better than the tetravalent **44** (Table 4, entry 1). In contrast, their corresponding deprotected counterparts **45** and **47** were almost negligible inhibitors showing only 17% and 27% inhibition, respectively (Table 4, entries 2 and 4). This disagreed with what we previously observed with the sugar-coated Au GNPs **6** and **7** (Figure 1), where the best inhibitory results were achieved using the corresponding deprotected carbohydrates with respect to the protected analogues [39,40].

The monovalent reference compound **48** exhibited very poor inhibition activity (16% inhibition; Table 4, entry 6) compared with its corresponding multivalent **44** and **46** as observed with the pyrrolidine derivatives discussed above. The nonavalent compound **50**, featuring simple pyrans as inhitopes, exhibited the same inhibition percentage (~30%) as its glucose-containing analogue (Table 4, entries 4 and 5). This result highlights that hydroxyl groups are not crucial for the interaction with the enzyme. Instead, it reinforces the pivotal role of the nonavalent scaffold itself in driving GALNS inhibition, even in the absence of hydroxyl groups. Furthermore, when compared with the nonavalent **38**, which incorporates pyrrolidines, the data suggest that tertiary amines contribute to the interaction, further influencing the inhibitory effect (Table 4, entry 5, vs. Table 1, entry 6).

The presence of pH-sensitive acetal groups in β-d-glucose-derived dendrimers led us to question whether these compounds would remain stable under the biological conditions of the screening assay. To assess their stability under the experimental conditions, we synthesized 2-(nonyloxy)tetrahydro-2H-pyran acetal following the literature procedure [63] as a control and conducted an NMR stability study. The NMR sample was prepared in D_2_O at pH* 4.3, where pH* refers to the pH measured in a D_2_O solution using an H_2_O-calibrated pH meter [64]. Spectra were recorded immediately after sample preparation and subsequently monitored over 17 h at 37 °C. The hydrolysis of the 2-(nonyloxy)tetrahydro-2H-pyran acetal yielded nonan-1-ol and 5-hydroxypentanal. The compound stability at pH* 4.3, assessed by ¹H-NMR experiments, is reported in the Supporting Information. The results indicated that acetal remained stable after 19 h at 37 °C and pH* 4.3, confirming the feasibility of the inhibitory assay for this class of β-d-glucose-derived dendrimers.

**Figure 3:**
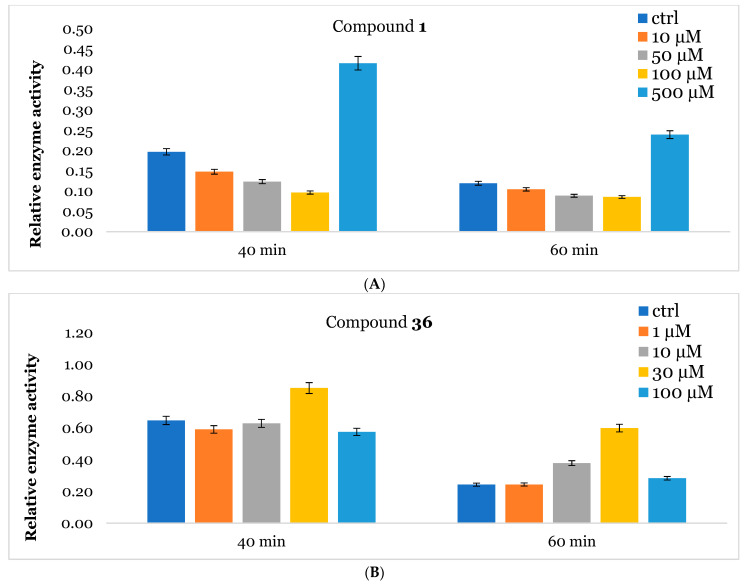
Stabilization of rhGALNS VIMIZIM^®^ in the presence of **1** (**A**) and **36** (**B**) was evaluated in vitro by using heat inactivation. Relative enzymatic activity after thermal treatment (48 °C) for 40 and 60 min at the indicated inhibitor concentrations (μM) with respect to the corresponding assay at 37 °C (no denaturation). Data for control were obtained as above except that no inhibitor was present. Experiments were performed in triplicate (see Supporting Information). Error bars reflect propagated uncertainties between two measurements.

The promising IC_50_ values observed for **1** and **36** prompted us to assess their effects on the commercially available wild-type recombinant GALNS (rhGALNS VIMIZIM^®^). The results showed comparable IC_50_ values of 110 µM for **1** and 30 µM for **36**, closely matching those obtained from leukocyte extracts (see Supporting Information). Building on these findings, we investigated the potential stabilizing effect of these compounds on the tertiary structure of GALNS through a thermal denaturation study. rhGALNS was preincubated with varying concentrations of the compounds before being subjected to heating at 48 °C for 40 and 60 min. The relative enzyme activity was expressed as the ratio between the activity measured after thermal denaturation at 48 °C and that obtained under physiological conditions at 37 °C. Notably, GALNS activity remained well preserved in the presence of **1** at the higher 500 µM concentration (around two-fold enzyme activity with respect to the control) across both time points (Figure 3A). In contrast, for compound **36**, the highest relative enzyme activity was observed at 30 µM, with increased enzyme activity by 1.3-fold and 2.4-fold with respect to the control, after 40 and 60 min of denaturation, respectively (Figure 3B). These findings suggested that both compounds could serve as potential stabilizers for the recombinant enzyme, with **36** showing the highest stabilization after 60 min of denaturation at a lower concentration [40].

## 4. Conclusions

The lysosomal enzyme *N*-acetylgalactosamine 6-sulfatase (GALNS) raised attention in relation to its important role in the field of lysosomal storage diseases and, more recently, due to its connection to cancer and neuropathic pain. Given its multimeric nature, GALNS is an enzyme prone to accepting multivalent ligands.

Following our ongoing interest in the identification of new GALNS inhibitors and stabilizers, we successfully synthesized in this work new iminosugar- and glucose-armed dendrimers through the CuAAC approach in quite good yields.

The new compounds, which differed for the type of the bioactive inhitope (pyrrolidine, pyrrolizidine, piperidine iminosugars, or carbohydrates), the valency, and the length of the linker between the bioactive inhitope and the triazole moiety, were preliminarily tested as GALNS inhibitors in leukocytes isolated from healthy donors, and their activity was compared with that of compounds previously synthesized in our group.

Among the screened compounds, the nonavalent dendrimers showed the highest inhibitory potency, and in particular, the nonavalent pyrrolidine compounds were confirmed as the best GALNS inhibitors since replacing them with other iminosugars (piperidine or pyrrolizidine) or with carbohydrates (either in their protected or deprotected form) did not improve the inhibitory properties of the multivalent adducts against GALNS. The observed superior performance of the nonavalent compounds with respect to lower-valent ones could be attributed to different effects, which are commonly invoked to explain the multivalent effect (statistical rebinding, chelating secondary binding, and cluster effects [34]). This was likely due to the specific spatial arrangement (presentation topology) of the bioactive units, which may enable the simultaneous engagement of multiple binding interactions or promote aggregation of the enzyme upon the binding. A deep investigation of which of these mechanisms are at work in this case is of great interest and will be the subject of future studies. Notably, we verified that the scaffold alone (compound **12**) exhibited negligible inhibitory activity (25%; see Supporting Information), suggesting that the enhanced potency of the nonavalent compounds was only minimally due to the core structure itself but could rather be attributed to the multivalency and spatial arrangement of the bioactive moieties. The nonavalent **36** featured by a slightly modified scaffold with respect to the previously reported **1** resulted in the best inhibitor reported in this work, with an IC_50_ value in the low micromolar range (IC_50_ = 28 μM), which was lower than that reported for **1** (IC_50_ = 47 μM). This result shows that the choice of the scaffold is crucial in the identification of more potent inhibitors. Conversely, the nonavalent dendrimer **38**, decorated with nine pyrrolidines and lacking the hydroxy groups, exhibited also a good inhibitory activity (IC_50_ = 70 μM), demonstrating the ability of the aromatic scaffold itself to inhibit GALNS enzyme and suggesting a synergistic effect between the nonavalent scaffold and the bioactive unit.

Building on the promising IC_50_ values of nonavalent dendrimers **1** and **36**, we investigated their stabilizing effects on GALNS through a thermal denaturation study. Both compounds preserved enzyme activity after 40 and 60 min of denaturation, with **1** maintaining high activity (almost two-fold vs. the control) at 500 µM, while **36** showed stabilization peaks at a much lower concentration (namely, 30 µM) with 2.4-fold enhanced activity with respect to the control after 60 min. These findings underscore their dual role as inhibitors and stabilizers, with **36** exhibiting a remarkable stabilization effect at lower concentration, which can be in principle exploited in a combined PC/ERT therapy for Morquio A syndrome.

## Figures and Tables

**Table 1:** Inhibitory activities of the monovalent pyrrolidine derivatives **34** and **35** and of the multivalent compounds **1**, **2**, **36**, and **38** toward *N*-acetylgalactosamine-6-sulfatase (GALNS).

Compound	Entry	Valency	% Inhibition (1 mM)	IC_50_ (µM)	rp	rp/n
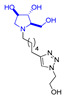 **34** (Ref. [37])	1	1	15 ^[a]^	3900 ^[a]^	-	-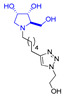 **35** (Ref. [37])	2	1	6 ^[a]^	5000 ± 200 ^[a]^	-	-
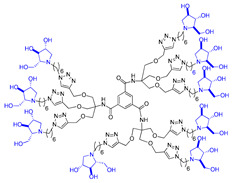 **1** (Ref. [37])	3	9	94 ^[a]^	47 ± 5 ^[a]^	83 ^[b]^ 1.5 ^[c]^	9.2 ^[b]^
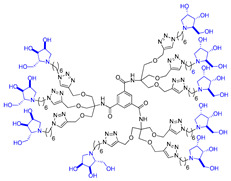 **2** (Ref. [37])	4	9	85 ^[a]^	85 ± 8 ^[a]^	59 ^[d]^ 0.8 ^[c]^	6.5 ^[d]^
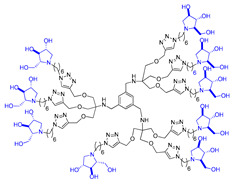 **36**	5	9	93	28 ± 5	139 ^[b]^	15.4 ^[b]^
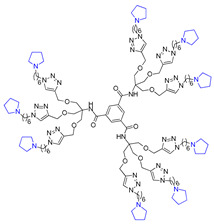 **38**	6	9	68	70 ± 15	-	-

^[a]^ Ref. [35]. ^[b]^ Calculated considering **34** as the monovalent reference compound. ^[c]^ Calculated with respect to the scaffold **38**. ^[d]^ Calculated considering **35** as the monovalent reference compound.

**Table 2:** Inhibitory activities of the multivalent pyrrolidines **16**, **19**, **23**, **26**–**29**, **30**–**33**, and **51** and of the multivalent pyrrolizidine compounds **52**–**54** toward *N*-acetylgalactosamine-6-sulfatase (GALNS).

Compound	% Inhibition (1 mM)	Compound	% Inhibition (1 mM)
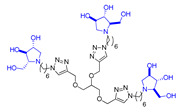 **28**	63	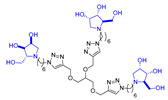 **29**	18
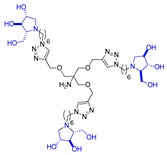 **26** (Ref. [51])	73	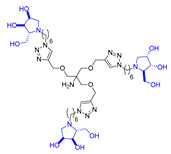 **27**	46
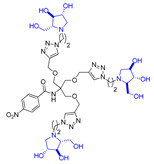 **51** (Ref. [55])	49	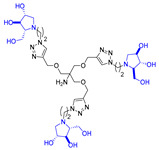 **23** (Ref. [55])	35
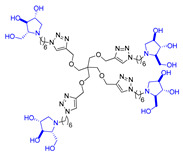 **30** (Ref. [51])	35	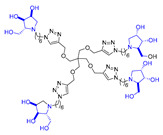 **31**	73
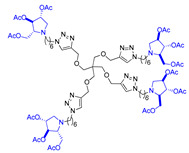 **16**	47	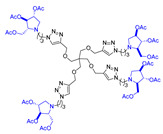 **19**	25
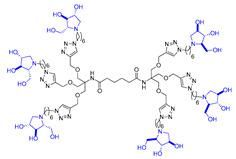 **32**	66	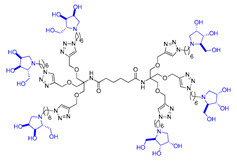 **33**	72 ^[a][b][c]^
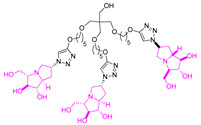 **52** (Ref. [44])	52	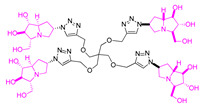 **54** (Ref. [44])	9
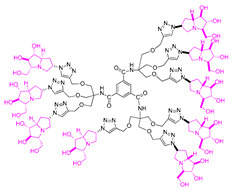 **53** (Ref. [44])	63		

^[a]^ IC_50_ = 350 ± 80. ^[b]^ rp = 14 evaluated considering **35** as the monovalent reference compound. ^[c]^ rp/n = 2.4 evaluated considering **35** as the monovalent reference compound.

**Table 3:** Inhibitory activities of the monovalent piperidine **55** and multivalent piperidines **56**–**58** toward *N*-acetylgalactosamine-6-sulfatase (GALNS).

Compound	% Inhibition (1 mM)	Compound	% Inhibition (1 mM)
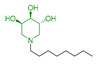 **55** (Ref. [61])	33	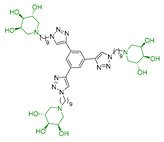 **57** (Ref. [62])	13
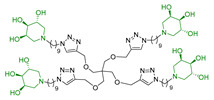 **56** (Ref. [62])	37	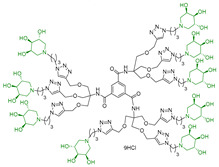 **58** (Ref. [52])	50

**Table 4:** Inhibitory activities of monovalent sugar compound **48** and multivalent sugar compounds **44**–**47** and multivalent compound **50** toward *N*-acetylgalactosamine-6-sulfatase (GALNS).

Compound	Entry	Valency	% Inhibition (1 mM)	IC_50_ (µM)	rp	rp/n
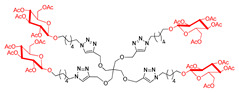 **44**	1	4	66	-	-	-
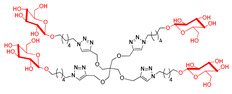 **45**	2	4	17	-	-	-
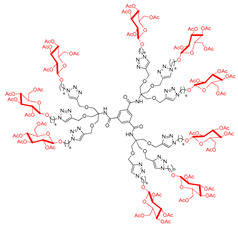 **46**	3	9	78	100 ± 20	-	-
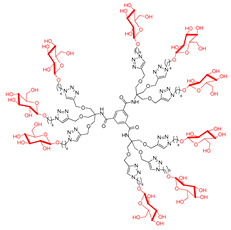 **47**	4	9	27	-	-	-
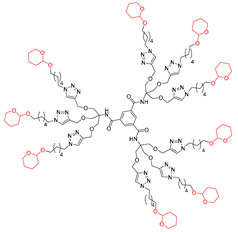 **50**	5	9	35	-	-	-
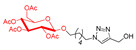 **48**	6	1	16	>>1 mM	-	-

## Data Availability

The original contributions presented in this study are included in the article/Supplementary Materials. Further inquiries can be directed to the corresponding authors.

## Supplementary Materials

The following supporting information can be downloaded at https://www.mdpi.com/article/10.3390/molecules30102222/s1: The thorough experimental section for this manuscript is detailed in the Supporting Information Refs. [65,66] are cited in Supplementary Materials.
