# Supplementary material for: Exploring Multivalent Architectures for Binding and Stabilization of N-Acetylgalactosamine 6-Sulfatase
Source: Molecules. 2025 May 20;30(10):2222. doi: 10.3390/molecules30102222 (PMC12113998; doi:10.3390/molecules30102222)
Supplement: Supplementary file 1 [file molecules-30-02222-s001.zip › molecules-3589949-supplementary.pdf]

Exploring Multivalent Architectures for Binding and Stabilization of *N*-Acetylgalactosamine 6-Sulfatase

Maria Giulia Davighi [1,†], Francesca Clemente[1,*,†], Giampiero D'Adamio[1], Macarena Martínez-Bailén[1,‡], Alessio Morano[1], Andrea Goti[1], Amelia Morrone[2,3], Camilla Matassini[1,4*] and Francesca Cardona[1,4*]

[1] Department of Chemistry "U. Schiff" (DICUS), University of Florence, via della Lastruccia 3-13, 50019 Sesto Fiorentino, Italy. giulia.davighi@gmail.com (M.G.D.); g.dadamio@fabotape.com (G.D.); mmartinez45@us.es (M.M.-B.); alessio.morano@unifi.it (A.M.); andrea.goti@unifi.it (A.G.)
[2] Laboratory of Molecular Biology of Neurometabolic Diseases, Neuroscience Department, Meyer Children's Hospital IRCCS, Viale Pieraccini 24, 50139 Firenze, Italy; amelia.morrone@unifi.it
[3] Department of Neurosciences, Psycology, Drug Research and Child Health University of Florence, Viale Pieraccini 24, 50139 Firenze, Italy.
[4] European Laboratory for Non-Linear Spectroscopy (LENS), University of Florence,50019 Sesto Fiorentino, Italy.
* Correspondence: francesca.clemente@unifi.it (F.C.); camilla.matassini@unifi.it (C.M.); francesca.cardona@unifi.it (F.C.)
† These authors contributed equally to the work.
‡ Current address: Departamento de Química Orgánica y Farmacéutica, Facultad de Farmacia, Universidad de Sevilla, C/Prof. García González, 2, 41012, Sevilla, Spain.

Electronic Supplementary Information (ESI)

Tables of contents

Deprotection of compound **46**	**S3**
Syntheses of new compounds	**S4**
NMR spectra	**S15**
Stability assessment at pH* 4.3 for 2-(nonyloxy)tetrahydro-2H-pyran acetal	**S44**
Enzymatic assays towards human GALNS in leukocytes isolated from healthy donors	**S45**
IC_{50} determination	**S47**
Stabilization of Recombinant GALNS under Thermal Denaturation Conditions	**S50**
References	**S51**

Deprotection of compound 46

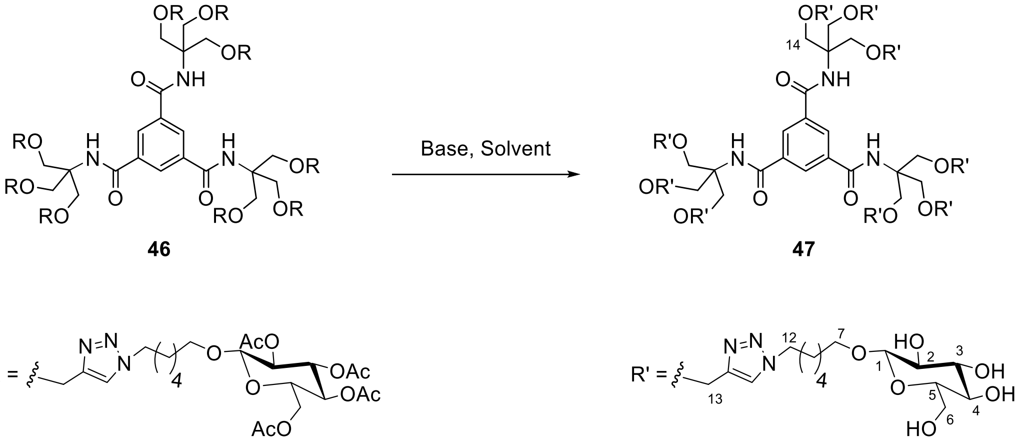

We faced challenges in the cleavage of the acetyl groups from compound **46**. The main attempts for the basic deprotection at room temperature are summarized in Table S1.

Entry	Base	Solvent	Time	Work up
1	Ambersep 900 OH	CD_3OD	8 h	Filtration
2	NaOMe	MeOH	80 min	Neutralization with Amberlyst 15 followed by filtration
3	Na_2CO_3	MeOH	18 h	Filtration
4	Na_2CO_3	MeOH	18 h	Neutralization with HCl 1% in MeOH followed by filtration

Table S1: attempts to obtain the deprotected **47**.

The treatment of **46** with the strongly basic resin Ambersep 900 OH (entry 1, Table S1) resulted in the degradation of the compound due to the concurrent hydrolysis of some amide groups during the deprotection of acetyl groups. Basic deprotection with NaOMe (entry 2, Table S1) followed by neutralization with the acid resin Amberlyst 15 produced the desired **47** along with an unidentified by-product which could not be separated from **46** using size exclusion chromatography (Bio-Beads SX-8). Deprotection with Na_2CO_3, followed by the filtration of the salts (entry 3, Table S1) led to the deprotected **47**; however, residual Na_2CO_3 was observed in the ^{13}C NMR at 161.2 ppm, which persisted despite further treatment with a size exclusion resin. Additionally, compound **47** could not be purified by flash column chromatography due to its high hydrophilicity and exclusive solubility in polar solvents (MeOH, H_2O). Finally, pure **47** was obtained by treating compound **46** with Na_2CO_3 for 18 hours, followed by neutralization of the reaction mixture with 1% HCl in MeOH and subsequent filtration (entry 4, Table S1), affording **47** in 63% yield.

Materials and Methods

General Experimental Procedures for the Syntheses

Commercial reagents were used as received. All reactions were carried out under magnetic stirring and monitored by TLC on 0.25 mm silica gel plates (Merck F254). Column chromatographies were carried out on Silica Gel 60 (32–63 μm) or on silica gel (230–400 mesh, Merck). Yields refer to spectroscopically and analytically pure compounds unless otherwise stated. Melting points were obtained with a Stuart Scientific melting point apparatus and are uncorrected. ^{1}H NMR and ^{13}C NMR spectra were recorded on a Varian Gemini 200 MHz, a Varian Mercury 400 MHz or on a Varian INOVA 400 MHz instrument at 25 °C. The notations of multiplicity (s, d, t, q) in the ^{13}C-NMR spectra do not derive from a decoupled experiment but are deduced on the basis of signal attribution through 2D spectra. ^{1}H-NMR and ^{13}C-NMR spectra were referenced against the residual solvent signal. [1] Integrals are in accordance with assignments, coupling constants are given in Hz. For detailed peak assignments 2D spectra were measured (COSY, HSQC). Small scale microwave assisted syntheses were carried out in a microwave apparatus for synthesis (CEM Discover) with an open reaction vessel and external surface sensor. IR spectra were recorded with a BX FT-IR Perkin-Elmer System or IRAffinity-1S SHIMADZU or IRAffinity-1 SHIMADZU system spectrophotometers. Optical rotation measurements were performed on a JASCO DIP-370 polarimeter. ESI-MS spectra were recorded with a Thermo Scientific™ LCQ fleet ion trap mass spectrometer. High Resolution Mass spectrometry (HRMS) were recorded with a Thermo Scientific™ LTQ Orbitrap equipped with an ESI IonMax source. Elemental analyses were performed with a Thermoscientific Flash Smart Elemental Analyzer CHNS/O.

Syntheses

Synthesis of Peracetylated tetravalent iminosugar **16**

To a solution of **15** [2] (113 mg, 0.0470 mmol) in methanol (20 mL), Pd/C (56 mg) and HCl 12M (60 μL) were added under nitrogen atmosphere. Then nitrogen was replaced by hydrogen gas, bubbling hydrogen with a balloon and the reaction mixture was stirred at room temperature for 3 days until ^{1}H NMR analysis assessed the disappearance of benzyl and aromatic protons. The catalyst was removed by filtration on a short pad of Celite and the solvent was evaporated under vacuum. The crude yellow oil was dissolved in pyridine (2 mL) and acetic anhydride (1 mL) was added. The solution was stirred at room temperature for 18 hours and then was concentrated under vaccum. The crude mixture was purified by FCC (CH_2Cl_2:MeOH 12:1) affording 42 mg of pure **16** (0.0230 mmol, 49% yield) as a pale yellow oil.

16: R_f = 0.28 (CH_2Cl_2:MeOH 12:1). $[\alpha]_D^{24}$ = - 43.0 (*c* = 0.80, $CHCl_3$); ^{1}H-NMR (400 MHz, $CDCl_3$): δ ppm = 7.55 (s, 4H, triazole), 5.09-5.01 (m, 8H, H-3, H-4), 4.54 (s, 8H, O*CH₂*Triazole), 4.31 (t, *J* = 7.2 Hz, 8H, H-12), 4.21-4.08 (m, 8H, H-6), 3.46 (s, 8H, CCH_2O), 3.12 (d, *J* = 11.2 Hz, 4H, Ha-5), 2.83-2.65 (m, 12H, Hb-5, Ha-7 and H-2), 2.37-2.27 (m, 4H, Hb-7), 2.07 (s, 12H, OAc), 2.06 (s, 24H, OAc), 1.95-1.83 (m, 8H, H-11), 1.52-1.39 (m, 8H, H-8), 1.39-1.28 (m, 16H, H-9, H-10). ^{13}C-NMR (100 MHz, $CDCl_3$): δ ppm = 171.0, 170.4, 169.8 (s, 12C, C=O), 145.4 (s, 4C, triazole), 122.5 (d, 4C, triazole), 78.9 (d, 4C, C-3), 76.5 (d, 4C, C-4), 69.3 (t, 4C, CCH_2O), 67.9 (d, 4C, C-2), 65.2 (t, 4C, OCH_2Triazole), 63.3 (t, 4C, C-6), 57.6 (t, 4C, C-5), 54.7 (t, 4C, C-7), 50.3 (t, 4C, C-12), 45.4 (s, *C*(CH_2)$_4$), 30.4 (t, 4C, C-11), 27.9 (t, 4C, C-8), 26.8, 26.5 (t, 8C, C-9, C-10), 21.2, 21.1, 21.0 (q, 12C, CH_3). IR ($CDCl_3$): $\tilde{\nu}$ = 2939, 2862, 2810, 1738, 1464, 1372, 1234, 1051 cm^{-1}. MS-ESI m/z (%) = 935.39 (100)$[M+2Na]^{2+}$. $C_{85}H_{132}N_{16}O_{28}$ (1826.05): calcd. C, 55.91, H, 7.29, N, 12.27; found: C, 55.93, H, 7.32, N, 12.22.

Synthesis of benzylated tetravalent iminosugars **18**

Azide **17** [3] (95 mg, 0.195 mmol), $CuSO_4$ (2 mg, 0.0125 mmol) and sodium ascorbate (6 mg, 0.0303 mmol) were added to a solution of tetravalent scaffold **10** [4] (14 mg, 0.0486 mmol) in THF (2 mL) and milliQ water (1 mL). The mixture was stirred under microwave irradiation at 80 °C for 45 minutes, until a TLC control (PEt:

EtOAc 3:1) attested the disappearance of the starting material **10** (R_f = 0.65) and the formation of a new product (R_f = 0.00). After filtration through Celite®, the solvent was removed under vacuum and the crude was purified by FCC (CH_2Cl_2:MeOH 20:1) to give 94 mg of pure **18** (0.0421 mmol, 86% yield) as a yellow oil.

18: R_f = 0.20 (CH_2Cl_2:MeOH 20:1). $[\alpha]_D^{25}$ = - 30.0 (*c* = 1.22, $CHCl_3$). ^{1}H-NMR (400 MHz, $CDCl_3$): δppm = 7.54 (s, 4H, triazole), 7.36-7.22 (m, 60H, Ar), 4.53-4.43 (m, 32H, C*H*$_2$Ph, OC*H*$_2$Triazole), 4.41-4.27 (m, 8H, H-9), 3.97-3.91 (m, 4H, H-4), 3.89-3.84 (m, 4H, H-3), 3.58-3.50 (m, 8H, H-6), 3.47 (s, 8H, CC*H*$_2$O), 3.19 (d, *J* = 10.6 Hz, 4H, Ha-5), 2.89 (dt, *J* = 12.2, 7.8 Hz, 4H, Ha-7), 2.77-2.70 (m, 4H, H-2), 2.61-2.51 (m, 4H, Hb-5), 2.45-2.35 (m, 4H, Hb-7), 2.11-1.99 (m, 8H, H-8). ^{13}C-NMR (100 MHz, $CDCl_3$): δppm = 145.2 (s, 4C, triazole), 138.3, 138.1 (s, 12C, Ar), 128.5, 128.4, 127.9, 127.8, 127.7 (d, 60C, Ar), 122.9 (d, 4C, triazole), 85.2 (d, 4C, C-3), 81.6 (d, 4C, C-4), 73.2 (t, 4C, *C*H$_2$Ph), 71.5 (t, 4C, *C*H$_2$Ph), 71.1 (t, 4C, *C*H$_2$Ph), 70.9 (t, 4C, C-6), 69.4 (t, 4C, C*CH$_2$*O), 69.3 (d, 4C, C-2), 65.1 (t, 4C, O*C*H$_2$Triazole), 57.3 (t, 4C, C-5), 52.1 (t, 4C, C-7), 48.2 (t, 4C, C-9), 45.4 (s, *C*(CH_2)$_4$), 29.0 (t, 4C, C-8). IR ($CDCl_3$): $\tilde{\nu}$ = 3088, 3067, 3032, 2864, 1497, 1454, 1366, 1213, 1095 cm^{-1}. MS-ESI (m/z, %) = 1139.80 (100) $[M+2Na]^{2+}$. $C_{133}H_{156}N_{16}O_{16}$ (2234.76): calcd. C, 71.48, H, 7.04, N, 10.03; found: C, 71.42, H, 7.01, N, 10.02.

Synthesis of peracetylated tetravalent iminosugar **19**

To a solution of **18** (94 mg, 0.0421 mmol) in methanol (15 mL), Pd/C (47 mg) and HCl 12M (60 µL) were added under nitrogen atmosphere. Then nitrogen was replaced by hydrogen gas, bubbling hydrogen with a balloon and the reaction mixture was stirred at room temperature for 3 days until a TLC analysis (CH_2Cl_2:MeOH 20:1) showed disappearance of the starting material (R_f = 0.20) and the formation of a new product (R_f = 0.00). Then, the mixture was filtered on a short pad of Celite and the solvent was removed under vacuum. The crude yellow oil was dissolved in pyridine (4 mL) and acetic anhydride (2 mL) was added. The solution was stirred at room temperature for 16 hours and then was concentrated under vacuum. The crude mixture was purified by FCC (CH_2Cl_2:MeOH 12:1) to give 26 mg of pure **19** (0.0157 mmol, 38% yield) as a pale yellow oil.

19: R_f = 0.25 (CH_2Cl_2:MeOH 12:1). $[\alpha]_D^{23}$ = - 31.4 (c = 0.7, $CHCl_3$). ^{1}H-NMR (400 MHz, $CDCl_3$): δppm = 7.62 (s, 4H, triazole), 5.14-5.02 (m, 8H, H-3, H-4), 4.54 (s, 8H, OC*H*$_2$Triazole), 4.49-4.32 (m, 8H, H-9), 4.26-4.19 (m, 4H, Ha-6), 4.13-4.05 (m, 4H, Hb-6), 3.46 (s, 8H, CC*H*$_2$O), 3.16 (d, *J* = 11.2 Hz, 4H, Ha-5), 2.86 (dt, *J* = 11.9, 7.8 Hz, 4H, Ha-7), 2.79-2.70 (m, 8H, Hb-5, H-2), 2.49-2.38 (m, 4H, Hb-7), 2.16-2.02 (m, 44H, OAc, H-8). ^{13}C-NMR (100 MHz, $CDCl_3$): δppm = 171.0, 170.4, 169.8 (s, 12C, C=O), 145.4 (s, 4C, triazole), 122.9 (d, 4C, triazole), 78.7 (d, 4C, C-3), 76.3 (d, 4C, C-4), 69.3 (t, 4C, C*C*H$_2$O), 68.1 (d, 4C, C-2), 65.1 (t, 4C, O*C*H$_2$Triazole), 63.1 (t, 4C, C-6), 57.5 (t, 4C, C-5), 51.6 (t, 4C, C-7), 48.1 (t, 4C, C-9), 45.4 (s, 1C, *C*(CH_2)$_4$), 29.0 (t, 4C, C-8), 21.2, 21.1 (q, 12C, CH_3). IR ($CDCl_3$): $\tilde{\nu}$ = 2939, 2862, 2811, 1738, 1464, 1372, 1233, 1051 cm^{-1}. MS-ESI (m/z, %) = 849.89 (100) $[M+2Na]^{2+}$. $C_{73}H_{108}N_{16}O_{28}$ (1657.73): calcd. C, 52.89, H, 6.57, N, 13.52; found: C, 52.87, H, 6.53, N, 13.50.

Synthesis of polyhydroxylated tetravalent iminosugar **20**

To a solution of **19** (19 mg, 0.0115 mmol) in MeOH (5 mL), the ion exchange resin Ambersep 900 OH (600 mg) was added and the suspension was slowly stirred at room temperature for 16 hours. The mixture was filtered, and the solvent was removed under vacuum affording 12 mg of pure **20** (0.0104 mmol, 95% yield) as a yellow oil.

20: $[\alpha]_D^{25}$ = - 38.5 (*c* = 1.1, MeOH). ^{1}H-NMR (400 MHz, D_2O): δ ppm = 7.92 (s, 4H, triazole), 4.47 (s, 8H, OC*H*$_2$Triazole), 4.41 (t, *J* = 6.9 Hz, 8H, H-9), 4.09-4.02 (m, 4H, H-4), 3.93-3.84 (m, 4H, H-3), 3.65-3.57 (m, 8H, H-6), 3.34 (s, 8H, CC*H*$_2$O), 2.98 (d, *J* = 11.3 Hz, 4H, Ha-5), 2.82-2.70 (m, 4H, Ha-7), 2.69-2.61 (m, 4H, Hb-5), 2.52-2.41 (m, 4H, H-2), 2.36-2.27 (m, 4H, Hb-7), 2.16-1.96 (m, 8H, H-8). ^{13}C-NMR (50 MHz, D_2O): δ ppm = 144.1 (s, 4C, triazole), 124.8 (d, 4C, triazole), 78.7 (d, 4C, C-3), 75.2 (d, 4C, C-4), 71.9 (d, 4C, C-2), 67.9 (t, 4C, C*C*H$_2$O), 63.4 (t, 4C, O*C*H$_2$Triazole), 60.7 (t, 4C, C-6), 58.2 (t, 4C, C-5), 51.5 (t, 4C, C-7), 48.3 (t, 4C, C-

9), 44.4 (s, 1C, *C*(CH_2)$_4$), 27.4 (t, 4C, C-8). MS-ESI (m/z, %) = 1175.85 (100) $[M+Na]^+$. $C_{49}H_{84}N_{16}O_{16}$ (1153.29): calcd. C, 51.03, H, 7.34, N, 19.43; found: C, 51.00, H, 7.30, N, 19.42.

Synthesis of polyhydroxylated trivalent iminosugar **23**

To a solution of compound **22** [4] (37 mg, 0.0224 mmol) in dry CH_2Cl_2 (2.2 mL), BCl_3 1M in hexane was added at 0 °C under nitrogen atmosphere. The reaction mixture was allowed to warm to room temperature and stirred for 18 hours until the disappearance of **22** was attested by a TLC analysis (CH_2Cl_2: MeOH 20: 1). Then, EtOH was added to the mixture until the visible precipitate was fully dissolved, and the solvent was removed under vacuum. The crude was dissolved in the minimum amount of H_2O, then the strongly basic resin Ambersep 900 OH was added and the mixture was stirred for 30 minutes. The resin was removed by filtration washing with MeOH and the solution was concentrated. The crude residue was purified by Size Exclusion Chromatography Sephadex LH-20 (eluting with H_2O) to yield 18 mg of pure **23** (0.0214 mmol, 96% yield).

23: $[\alpha]_D^{22}$ = - 17.6 (*c* = 0.99, H_2O). ^{1}H-NMR (400 MHz, D_2O): δppm = 8.09 (s, 3H, triazole), 4.72-4.52 (m, 12H, OC*H$_2$*Triazole, H-8), 4.15-4.08 (m, 3H, H-4), 3.95-3.88 (m, 3H, H-3), 3.66-3.51 (m, 12H, H-6, CC*H$_2$*O), 3.43-3.31 (m, 3H, Ha-7), 3.01-2.92 (m, 6H, Ha-5, Hb-7), 2.83-2.73 (m, 3H, Hb-5), 2.66-2.57 (m, 3H, H-2). ^{13}C-NMR (50 MHz, D_2O): δppm = 143.7 (s, 3C, triazole), 125.3 (d, 3C, triazole), 78.6 (d, 3C, C-3), 75.4 (d, 3C, C-4), 71.5 (d, 3C, C-2), 63.5 (t, 3C, O*C*H_2Triazole), 60.7 (t, 6C, C-6, C*C*H_2O), 59.5 (s, 1C, H_2N*C*(CH_2)$_3$), 58.1 (t, 3C, C-5), 53.8 (t, 3C, C-7), 48.8 (t, 3C, C-8). MS-ESI (m/z, %) = 297.74 (100) $[M+2Na+H]^{3+}$, 864.47 (28) $[M+Na]^+$. $C_{34}H_{59}N_{13}O_{12}$ (841.91): calcd. C, 48.50, H, 7.06, N, 21.63; found: C, 48.88, H, 7.23, N, 21.50.

Synthesis of polyhydroxylated trivalent iminosugar **28**

Azide **24** [5] (44 mg, 0.176 mmol), $CuSO_4$ (3 mg, 0.0160 mmol) and sodium ascorbate (6 mg, 0.0320 mmol) were added to a solution of alkyne **9** [6] (11 mg, 0.0533) in THF (2 mL) and milliQ water (1 mL). The reaction mixture was stirred in a MW reactor at 80°C for 45 minutes until a TLC analysis (PEt:EtOAc 4:1) showed the disappearance of the starting material (R_f = 0.45) and formation of the desired product (R_f=0.00). After filtration through Celite®, the solvent was removed under reduced pressure and subsequently the crude product was treated with the “Quadrasil MP®” resin keeping the mixture under stirring at room temperature in the minimum amount of H_2O for 1 hour (1 g of resin for each mmol of copper). Then, the resin was filtered off through Celite® and the crude was purified by FCC (CH_2Cl_2:MeOH:NH_4OH(6%) 3:3:0.1), affording 40 mg of **28** (0.0408 mmol, 78% yield) as yellow oil.

28: R_f = 0.85 (CH_2Cl_2:MeOH:NH_4OH(6%) 3:3:1). $[\alpha]_D^{24}$ = - 16.5 (c = 0.57, MeOH). ^{1}H-NMR (400 MHz, CD_3OD): δppm = 7.95 (s, 2H, triazole), 7.93 (s, 1H, triazole), 4.69-4.54 (m, 6H, OC*H$_2$*Triazole), 4.42-4.32 (m, 6H, H-12), 4.15-4.08 (m, 3H, H-4), 3.96-3.89 (m, 3H, H-3), 3.80-3.75 (m, 1H, OCH_2C*H*CH_2O), 3.76-3.69 (m, 6H, H-6), 3.66-3.60 (m, 2H, OC*H$_2$*CHCH_2O), 3.57-3.51 (m, 2H, OCH_2CHC*H$_2$*O), 3.15-3.05 (m, 3H, Ha-5), 2.96-2.81 (m, 6H, Hb-5, Ha-7), 2.77-2.66 (m, 3H, H-2), 2.55-2.42 (m, 3H, Hb-7), 1.93-1.74 (m, 6H, H-11), 1.55-1.38 (m, 6H, H-8), 1.31-1.13 (m, 12H, H-9, H-10). ^{13}C-NMR (100 MHz, CD_3OD): δppm = 145.9 (s, 3C, triazole), 125.1, 125.0 (d, 3C, triazole), 111.4 (interference), 79.6 (d, 3C, C-3), 79.4 (d, 1C, OCH_2*C*HCH_2O), 76.7 (d, 3C, C-4), 76.6 (d, 3C, C-2), 76.2 (t, 2C, O*C*H_2CH*C*H_2O), 65.2 (t, 3C, O*C*H_2Triazole), 61.8 (t, 3C, C-6), 60.6 (t, 3C, C-5), 57.4 (t, 3C, C-7), 51.2 (t, 3C, C-12), 31.1 (t, 3C, C-11), 27.7, 27.4, 27.1 (t, 9C, C-8, C-9, C-10). MS-ESI (m/z, %) = 1003.75 (100) $[M+Na]^+$. $C_{45}H_{80}N_{12}O_{12}$ (981.19): calcd. C, 55.08, H, 8.22, N, 17.13; found: C, 54.84, H, 8.17, N, 17.16.

Synthesis of polyhydroxylated hexavalent iminosugar **32**

Azide **24** [5] (42 mg, 0.163 mmol), $CuSO_4$ (1 mg, 0.00774 mmol) and sodium ascorbate (3 mg, 0.0155 mmol) were added to a solution of hexavalent scaffold **11** [7] (15 mg, 0.0258) in THF (2 mL) and milliQ water (1 mL). The reaction mixture was stirred in a MW reactor at 80°C for 1 h and 30 min until TLC analysis (PEt:EtOAc 1:1) showed the disappearance of the starting material (R_f=0.22) and formation of the desired product (R_f=0.00). After filtration through Celite®, the solvent was removed under reduced pressure and subsequently

the crude product was treated with the "Quadrasil MP®" resin keeping the mixture under stirring at room temperature in the minimum amount of H_2O for 1 hour (1 g of resin for each mmol of copper). Then the resin was filtered off through Celite® and the crude was purified by Size Exclusion Chromatography Sephadex LH-20 (eluting with H_2O) affording 41 mg of pure **32** (0.0192 mmol, 73% yield) as a yellow oil.

32: $[\alpha]_D^{27}$ = - 18.2 (c = 0.3, MeOH). ¹H-NMR (400 MHz, D_2O): δ ppm = 7.92-7.86 (m, 6H, triazole), 4.55-4.45 (m, 12H, O*CH₂*Triazole), 4.39-4.28 (m, 12H, H-12), 4.11-4.03 (m, 6H, H-4), 3.92-3.86 (m, 6H, H-3), 3.71-3.65 (m, 12H, H-6) 3.63 (s, 12H, CCH_2O), 3.06-2.94 (m, 6H, Ha-5), 2.88-2.65 (m, 12H, Ha-7, Hb-5), 2.62-2.47 (m, 6H, H-2), 2.45-2.23 (m, 6H, Hb-7), 2.17-2.06 (m, 4H, CH_2CH_2C=O), 1.87-1.73 (m, 12H, H-11), 1.62-1.52 (m, 4H, CH_2CH_2C=O), 1.51-1.30 (m, 12H, H-8), 1.28-1.11 (m, 24H, H-9, H-10). ¹³C-NMR (100 MHz, D_2O): δ ppm = 176.5, 176.0 (s, 2C, C=O), 143.9 (s, 6C, triazole), 124.7 (d, 3C, triazole), 124.6 (d, 3C, triazole), 78.9, 78.8, 78.7 (d, 6C, C-3), 75.3, 75.2 (d, 6C, C-4), 72.1 (d, 6C, C-2), 67.4, 67.2 (t, 6C, CCH_2O), 63.5, 63.4 (t, 6C, OCH_2Triazole), 61.0, 60.9 (t, 6C, C-6), 59.7, 59.6, (s, 2C, CCH₂O), 58.3 (t, 6C, C-5), 55.4, 55.3, 55.2 (t, 6C, C-7), 51.1 50.2 (t, 6C, C-12), 37.2, 36.0, 35.8 (t, 2C, CH_2CH_2C=O), 29.3, 29.2 (t, 6C, C-11), 27.8, 26.4, 26.3, 26.2, 26.0, 25.7, 25.4, 25.3, 25.2 (t, 20C, C-8, C-9, C-10, CH_2CH_2C=O). MS-ESI (m/z, %) = 1088.50 (100) $[M+2Na]^{2+}$. $C_{98}H_{172}N_{26}O_{26}$ (2130.57): calcd. C, 55.25, H, 8.14, N, 17.09; found: C, 55.29, H, 8.19, N, 16.97.

General procedure for CuAAC reaction to synthesize multivalent compounds **27**, **29**, **31** and **33**

Azide **25** [5] (appropriate equivalents), $CuSO_4$ (0.3 equiv) and sodium ascorbate (0.6 equiv) were added to a solution of alkyne **8** [8-9]-**9** [6]((1 equiv.) in THF (2 mL) and milliQ water (1 mL). The reaction mixture was stirred in a MW reactor at 80°C for 45 min until TLC analysis (PEt:EtOAc 4:1) showed the disappearance of the starting material (R_f = 0.45) and formation of the desired product. After filtration through Celite®, the solvent was removed under reduced pressure and the crude was purified by size exclusion chromatography (Sephadex LH-20® resin) and/or by FCC to obtain the multivalent adducts **27, 29, 31 and 33**.

Synthesis of polyhydroxylated trivalent iminosugar **27**

Application of the general procedure to scaffold **8** (13 mg, 0.0553 mmol) with 3.3 equiv. of **25**(47 mg, 0.182 mmol), $CuSO_4$ (3 mg, 0.0166 mmol) and sodium ascorbate (7 mg, 0.0332 mmol) gave, after purification by Sephadex LH-20 resin (eluting with MeOH), 33 mg of **27** (0.0327 mmol, 62% yield) as yellow oil.

27: $[\alpha]_D^{23}$ = - 11.1 (c = 0.73, MeOH). ¹H-NMR (400 MHz, D_2O): δ ppm = 7.92 (s, 3H, triazole), 4.53 (s, 6H, OCH_2Triazole), 4.35 (t, J = 6.9 Hz, 6H, H-12), 4.22-4.13 (m, 3H, H-4), 3.99 (t, J = 5.7 Hz, 3H, H-3), 3.75-3.66 (m, 6H, H-6), 3.49-3.42 (m, 3H, Ha-5), 3.40 (s, 6H, CCH_2O), 3.10-2.91 (m, 6H, H-2, Ha-7), 2.82-2.63 (m, 6H, Hb-5, Hb-7), 1.89-1.77 (m, 6H, H-11), 1.59-1.43 (m, 6H, H-8), 1.33-1.15 (m, 12H, H-9, H-10). ¹³C-NMR (100 MHz, D_2O): δ ppm = 143.8 (s, 3C, triazole), 124.7 (d, 3C, triazole), 71.4 (d, 3C, C-3), 70.5 (d, 3C, C-2), 69.3 (t, 3C, CCH_2O), 68.7 (d, 3C, C-4), 63.5 (t, 3C, OCH_2Triazole), 58.7 (t, 3C, C-6), 56.9 (t, 3C, C-5 and s, 1C, CCH₂O), 56.5 (t, 3C, C-7), 50.2 (t, 3C, C-12), 29.0 (t, 3C, C-11), 25.4 (t, 3C, C-8), 25.2, 25.1 (t, 6C, C-9, C-10). MS-ESI (m/z, %) = 1032.83 (100) $[M+Na]^+$. $C_{46}H_{83}N_{13}O_{12}$ (1010.23): calcd. C, 54.69, H, 8.28, N, 18.02; found: C, 54.71, H, 8.30, N, 18.00.

Synthesis of polyhydroxylated trivalent iminosugar **29**

Application of the general procedure to scaffold **9** (15 mg, 0.0727 mmol) with 3.3 equiv. of **25** (63 mg, 0.244 mmol), $CuSO_4$ (3 mg, 0.0218 mmol) and sodium ascorbate (9 mg, 0.0436 mmol) gave, after purification by FCC (CH_2Cl_2:MeOH:NH_4OH(6%) 3:2:1), 49 mg of **29** (0.0499 mmol, 67% yield) as a yellow waxy solid.

29: R_f = 0.75 (CH_2Cl_2:MeOH:NH_4OH(6%) 3:2:0.1). $[\alpha]_D^{26}$ = - 22.7 (c = 0.34, MeOH). ¹H-NMR (400 MHz, D_2O): δ ppm = 8.00 (s, 2H, triazole), 7.98 (s, 1H, triazole), 4.70 (s, 2H, OCH_2Triazole), 4.62 (s, 4H, OCH_2Triazole), 4.49-4.35 (m, 6H, H-12), 4.18-4.08 (m, 3H, H-4), 3.94 (t, J = 4.9 Hz, 3H, H-3), 3.87-3.78 (m, 1H, OCH₂CHCH₂O), 3.72-3.56 (m, 10H, H-6, OCH_2CHCH_2O), 3.31-3.23 (m, 3H, Ha-5), 2.87-2.75 (m, 3H, Ha-7), 2.73-2.65 (m, 3H, H-2), 2.55-2.38 (m, 6H, Hb-5, Hb-7), 1.97-1.79 (m, 6H, H-11), 1.54-1.37 (m, 6H, H-8), 1.33-

1.18 (m, 12H, H-9, H-10). ^{13}C-NMR (100 MHz, D_2O): δ ppm = 144.1 (s, 1C, triazole), 143.8 (s, 2C, triazole), 124.8 (d, 3C, triazole), 76.4 (d, 1C, $OCH_2\mathit{C}HCH_2O$), 72.4 (d, 3C, C-3), 70.8 (d, 3C, C-2), 69.0 (d, 3C, C-4), 68.8 (t, 3C, C-6), 63.3 (t, 2C, $O\mathit{C}H_2CH\mathit{C}H_2O$), 62.0 (t, 2C, $O\mathit{C}H_2$Triazole), 60.9 (t, 1C, $O\mathit{C}H_2$Triazole), 56.6 (t, 3C, C-5), 55.8 (t, 3C, C-7), 50.3 (t, 3C, C-12), 29.1 (t, 3C, C-11), 26.3 (t, 3C, C-8), 25.9, 25.2 (t, 6C, C-9, C-10). MS-ESI (m/z, %) = 1003.75 (100) [M+Na]$^+$. $C_{45}H_{80}N_{12}O_{12}$ (981.19): calcd. C, 55.08, H, 8.22, N, 17.13; found: C, 54.97, H, 8.11, N, 17.06.

Synthesis of polyhydroxylated tetravalent iminosugars **31**

Application of the general procedure to scaffold **10** [4] (9 mg, 0.0312 mmol) with 4 equiv. of **25** [5] (32 mg, 0.125 mmol), $CuSO_4$ (1 mg, 0.00936 mmol) and sodium ascorbate (4 mg, 0.0187 mmol) gave, after purification by FCC (MeOH:CH_2Cl_2:NH_4OH 1:1:0.3) and Size Exclusion Chromatography Sephadex LH-20 (eluting with H_2O), 29 mg of **31** (0.0219 mmol, 73% yield) as a yellow oil.

31: R_f = 0.30 (MeOH:CH_2Cl_2:NH_4OH 1:1:0.3). $[\alpha]_D^{21}$ = - 11.0 (c = 0.46, MeOH). ^{1}H-NMR (400 MHz, D_2O): δ ppm = 7.82 (s, 4H, triazole), 4.40 (s, 8H, $OC\mathit{H_2}$Triazole), 4.27 (t, J = 6.8 Hz, 8H, H-12), 4.12-4.04 (m, 4H, H-4), 3.90 (t, J = 5.3 Hz, 4H, H-3), 3.65-3.57 (m, 8H, H-6), 3.33-3.28 (m, 4H, Ha-5), 3.27 (s, 8H, $CC\mathit{H_2}O$), 2.90-2.81 (m, 4H, Ha-7), 2.80-2.74 (m, 4H, H-2), 2.59-2.43 (m, 8H, Hb-5, Hb-7), 1.81-1.68 (m, 8H, H-11), 1.48-1.31 (m, 8H, H-8), 1.22-1.07 (m, 16H, H-9, H-10). ^{13}C-NMR (100 MHz, D_2O): δ ppm = 144.1 (s, 4C, triazole), 124.5 (d, 4C, triazole), 72.0 (d, 4C, C-3), 70.7 (d, 4C, C-2), 68.9 (d, 4C, C-4), 67.9 (t, 4C, $C\mathit{CH_2}O$), 63.4 (t, 4C, $O\mathit{CH_2}$Triazole), 60.1 (t, 4C, C-6), 56.8 (t, 4C, C-5), 56.0 (t, 4C, C-7), 50.2 (t, 4C, C-12), 44.4 (s, 1C, $\mathit{C}(CH_2)_4$), 29.2 (t, 4C, C-11), 26.0 (t, 4C, C-8), 25.7, 25.2 (t, 8C, C-9, C-10). MS-ESI (m/z, %) = 1343.90 (100) [M+Na]$^+$. $C_{61}H_{108}N_{16}O_{16}$ (1321.61): calcd. C, 55.44, H, 8.24, N, 16.96; found: C, 55.40, H, 8.22, N, 16.93.

Synthesis of polyhydroxylated hexavalent iminosugar **33**

Application of the general procedure to scaffold **11** [7] (25 mg, 0.0431) with 6.3 equiv. of **25** [5] (70 mg, 0.272 mmol), $CuSO_4$ (2 mg, 0.0129 mmol) and sodium ascorbate (5 mg, 0.0259 mmol) gave, after purification by Size Exclusion Chromatography Sephadex LH-20 (eluting with H_2O), 66 mg of pure **33** (0.0310 mmol, 72% yield) as a yellow waxy solid.

33: $[\alpha]_D^{25}$ = - 63.9 (c = 1.1, H_2O). ^{1}H-NMR (400 MHz, D_2O): δ ppm = 7.83 (s, 6H, triazole), 4.43 (s, 12H, $OC\mathit{H_2}$Triazole), 4.28 (t, J = 6.8 Hz, 12H, H-12), 4.11-4.05 (m, 6H, H-4), 3.90 (t, J = 5.2 Hz, 6H, H-3), 3.64-3.54 (m, 24H, H-6, $CC\mathit{H_2}O$), 3.32-3.23 (m, 6H, Ha-5), 2.90-2.79 (m, 6H, Ha-7), 2.79-2.72 (m, 6H, Ha-2), 2.61-2.42 (m, 12H, Hb-7, Hb-5), 2.11-1.98 (m, 4H, $CH_2C\mathit{H_2}C{=}O$), 1.81-1.69 (m, 12H, H-11), 1.48-1.32 (m, 16H, $C\mathit{H_2}CH_2C{=}O$, H-8), 1.25-1.08 (m, 24H, H-9, H-10). ^{13}C-NMR (100 MHz, D_2O): δ ppm = 176.1 (s, 2C, C=O), 143.9 (s, 6C, triazole), 124.6 (d, 6C, triazole), 72.0 (d, 6C, C-3), 70.7 (d, 6C, C-2), 68.9 (d, 6C, C-4), 67.3 (t, 6C, $C\mathit{C}H_2O$), 63.4 (t, 6C, $O\mathit{C}H_2$Triazole), 60.1 (t, 6C, C-6), 59.6 (s, 2C, $\mathit{C}CH_2O$), 56.8 (t, 6C, C-5), 56.1 (t, 6C, C-7), 50.2 (t, 6C, C-12), 35.8 (t, 2C, $\mathit{C}H_2CH_2C{=}O$), 29.2 (t, 6C, C-11), 25.9, 25.7, 25.2 (t, 18C, C-8, C-9, C-10), 24.5 (t, 2C, $\mathit{C}H_2CH_2C{=}O$). MS-ESI (m/z, %) = 1119.58 (100) [M+2Na+MeOH]$^{2+}$, 727.67 (54) [M+H+2Na]$^{3+}$. $C_{98}H_{172}N_{26}O_{26}$ (2130.57): calcd. C, 55.25, H, 8.14, N, 17.09; found: C, 55.35 H, 8.20, N, 16.99.

Synthesis of nonavalent scaffold **13**

To a solution of tris[(propargyloxy)methyl]aminomethane **8** [8-9] (59 mg, 0.251 mmol) in CH_3CN (1 mL), K_2CO_3 (35 mg, 0.251 mmol) and 1,3,5-Tris(bromomethyl)benzene (20 mg, 0.0560) were added. The mixture was stirred at room temperature for 18 hours until a TLC analysis (PEt:EtOAc 1:1) showed the disappearance of 1,3,5-Tris(bromomethyl)benzene. The solvent was removed under vacuum and the crude was purified by FCC on silica gel (PEt:EtOAc 1:1) affording 31 mg of **13** (0.0381 mmol, 68% yield).

13: R_f = 0.33 (PEt:EtOAc 1:1). ^{1}H-NMR (400 MHz, $CDCl_3$): δ ppm = 7.23 (s, 3H, Ar), 4.23-4.12 (m, 18H, $OC\mathit{H_2}C{\equiv}C$), 3.82 (s, 6H, $C\mathit{H_2}N$), 3.59 (s, 18H, $CC\mathit{H_2}O$), 2.45 (t, J = 2.3 Hz, 9H, $C{\equiv}C\mathit{H}$), 2.21-1.93 (br s, 3H,

N*H*). ^{13}C-NMR (100 MHz, $CDCl_3$): δppm = 140.9 (s, 3C, Ar), 127.3 (d, 3C, Ar), 80.0 (s, 9C, *C*≡CH), 74.7 (d, 9C, C≡*C*H), 69.8 (t, 9C, C*C*H_2O), 59.7 (s, 3C, *C*CH_2O), 58.8 (t, 9C, *C*H_2C≡C), 46.8 (t, 3C, *C*H_2N). MS-ESI (m/z, %) = 820.50 (26) $[M+H]^+$, 842.67 (100) $[M+Na]^+$. $C_{48}H_{57}N_3O_9$ (819.98): calcd. C, 70.31, H, 7.01, N, 5.12; found: C, 70.10, H, 7.15, N, 5.33.

Synthesis of nonavalent compound **36**

Azide **24** [5] (41 mg, 0.158 mmol), $CuSO_4$ (1 mg, 0.00510 mmol) and sodium ascorbate (2 mg, 0.0102 mmol) were added to a solution of nonavalent scaffold **13** (13 mg, 0.0170) in THF (2 mL) and milliQ water (1 mL). The reaction mixture was stirred in a MW reactor at 80°C for 45 min until TLC analysis (PEt:EtOAc 1:1) showed the disappearance of the starting material (R_f=0.22) and formation of the desired product (R_f=0.00). After filtration through Celite®, the solvent was removed under vacuum and subsequently the crude product was treated with the "Quadrasil MP®" resin keeping the mixture under stirring at room temperature in the minimum amount of a 1:1 H_2O:MeOH mixture for 1 hour (1 g of resin for each mmol of copper). Then the resin was filtered off through Celite® and the crude was purified by Size Exclusion Chromatography Sephadex LH-20 (eluting with H_2O) affording 44 mg of pure **36** (0.0139 mmol, 82% yield) as a pale yellow waxy solid.

36: $[\alpha]_D^{25}$ = - 46.7 (c = 0.9, MeOH). ^{1}H-NMR (400 MHz, D_2O): δppm detected signals = 7.89 (s, 9H, triazole), 7.08-6.78 (m, 3H, Ar), 4.55-4.38 (m, 18H, O*CH_2*Triazole), 4.34-4.16 (m, 18H, H-12), 4.11-3.96 (m, 9H, H-4), 3.91-3.82 (m, 9H, H-3), 3.71-3.59 (m, 18H, H-6), 3.55-3.31 (m, 18H, C*CH_2*O), 3.06-2.89 (m, 9H, Ha-5), 2.84-2.61 (m, 18H, Hb-5, Ha-7), 2.60-2.44 (m, 9H, H-2), 2.39-2.19 (m, 9H, Hb-7), 1.81-1.60 (m, 18H, H-11), 1.43-1.25 (m, 18H, H-8), 1.19-1.01 (m, 36H, H-9, H-10). ^{13}C-NMR (100 MHz, D_2O): δppm detected signals = 144.1 (s, 9C, triazole), 124.6 (d, 9C, triazole), 78.3 (d, 9C, C-3), 75.0 (d, 9C, C-4), 72.6 (d, 9C, C-2), 68.1 (t, 9C, C*C*H_2O), 63.4 (t, 9C, O*C*H_2Triazole), 60.5 (t, 9C, C-6), 58.3 (t, 9C, C-5), 55.4 (t, 9C, C-7), 50.2 (t, 9C, C-12), 29.2 (t, 9C, C-11), 26.0, 25.8, 25.3 (t, 27C, C-8, C-9, C-10). Some protons (C*H2*NH) and some carbons (Ar, Ar*C*H2NH and HN*C*$(CH_2O)_3$) are missing due to some relaxation and acquisition factors. MS-ESI (m/z, %) = 1056.42 (100) $[M+H+2Na]^{3+}$. $C_{147}H_{255}N_{39}O_{36}$ (3142.93): calcd. C, 56.14, H, 8.17, N, 17.37; found: C, 56.24, H, 8.08, N, 17.07.

Synthesis of compound 1-(6-azidohexyl)pyrrolidine **37**

A solution of pyrrolidine (77 mg, 1.08 mmol), 1-azido-6-bromohexane (334 mg, 1.62 mmol) and K_2CO_3 (226 mg, 1.62 mmol) in a 5:1 CH_3CN:H_2O mixture (4.8 mL), was stirred in a MW reactor at 120 °C for 3 hours. Then, the crude was filtered through Celite® and the solvent was removed under vaccum. The resulting residue was purified by FCC on silica gel (gradient eluent from EtOAc:MeOH:NH_4OH (6%) 10:1:0.1 to 2:1:0.1) to give 77 mg of the desired compound **37** (0.392 mmol, 36% yield) as a colourless oil.

37: R_f =0.38 (EtOAc:MeOH:NH_4OH (6%) 10:1:0.1). ^{1}H-NMR (400 MHz, $CDCl_3$): δppm = 3.24 (t, 2H, J = 6.9, H-6), 2.80-2.67 (m, 4H, H-2, H-5), 2.65-2.56 (m, 2H, H-11), 1.88 (quint, 4H, J = 3.4, H-3, H-4), 1.69-1.54 (m, 4H, H-7, H-10), 1.43-1.29 (m, 4H, H-8, H-9). ^{13}C-NMR (100 MHz, $CDCl_3$): δppm = 56.1 (t, 1C, C-11), 54.0 (t, 2C, C-2, C-5), 51.3 (t, 1C, C-6), 28.7, 27.8 (t, 2C, C-7, C-10), 26.9, 26.5 (t, 2C, C-8, C-9), 23.4 (t, 2C, C-3, C-4). MS-ESI (m/z, %) = 197.25 (100) $[M+H]^+$. IR ($CDCl_3$): $\tilde{\nu}$ = 2938, 2863, 2801, 2100, 1460, 1259 cm^{-1}. $C_{10}H_{20}N_4$ (196.30): calcd. C, 61.19, H 10.27, N 28.54; found C, 61.58, H 10.21, N 28.26.

Synthesis of nonavalent compound **38**

Nonapropargylated scaffold **12** [10] (15 mg, 0.0174 mmol), $CuSO_4$ (1 mg, 0.00522 mmol) and sodium ascorbate (2 mg, 0.0104 mmol) were added to a solution of **37** (33 mg, 0.168 mmol) in THF (2 mL) and milliQ water (1 mL). The reaction mixture was stirred in a MW reactor at 80 °C for 45 min. After filtration through Celite®, the solvent was removed under vacuum and subsequently the crude product was treated with the "Quadrasil MP®" resin keeping the mixture under stirring at room temperature in the minimum amount of MeOH for 1 hour (1 g of resin for each mmol of copper). Then the resin was filtered off through Celite® and the

crude was purified by Size Exclusion Chromatography Sephadex LH-20 (eluting with MeOH) affording 41 mg of pure **38** (0.0156 mmol, 90% yield) as a yellow oil.

38: ^{1}H-NMR (400 MHz, CD_3OD): δ ppm = 8.21 (s, 3H, Ar), 8.03 (s, 9H, triazole), 4.60 (s, 18H, OCH_2Triazole), 4.39 (t, 18H, J = 6.9, H-11), 3.91 (br s, 18H, CCH_2O), 3.18-2.99 (m, 36H, H-2, H-5), 2.98-2.84 (m, 18H, H-6), 2.05-1.94 (m, 36H, H-3, H-4), 1.93-1.83 (m, 18H, H-10), 1.74-1.56 (m, 18H, H-7), 1.44-1.27 (m, 36H, H-8, H-9). ^{13}C-NMR (100 MHz, CD_3OD): δ ppm = 168.7 (s, 3C, C=O), 145.8 (s, 9C, triazole), 137.2 (s, 3C, Ar), 130.1 (d, 3C, Ar), 125.2 (d, 9C, triazole), 69.2 (t, 9C, C*C*H_2O), 65.4 (t, 9C, O*C*H_2Triazole), 62.2 (s, 3C, *C*CH_2O), 56.5 (t, 9C, C-6), 55.0 (t, 18C, C-2, C-5), 51.2 (t, 9C, C-11), 31.1 (t, 9C, C-10), 27.7 (t, 9C, C-7), 27.4, 27.1 (t, 18C, C-8, C-9), 24.1 (t, 18C, C-3, C-4). IR (neat): $\tilde{\nu}$ = 3414, 3254, 2934, 1661, 1539, 1456, 1288, 1086. HRESIMS calcd for $C_{138}H_{231}N_{39}O_{12}$ $[M+5H]^{5+}$: 526.38056; found 526.38103.

Synthesis of (6-Chlorohexyl)-2,3,4,6-tetra-*O*-acetyl-β-D-glucopyranoside **41** (*Route II*) and (6-Chlorohexyl)-2,3,4,6-tetra-*O*-acetyl-α-D-glucopyranoside **40**

A suspension of peracetylated glucose **39** [11] (200 mg, 0.512 mmol), 6-chlorohexanol (102 µl, 0.761 mmol) and 3Å MS in dry DCM (2 mL) was cooled to 0 °C and $BF_3{\cdot}OEt_2$ (316 µl, 2.56 mmol) was added dropwise. The reaction mixture was stirred at room temperature for 26 hours until a the disappearance of **39** was attested by a TLC control (Hexane:EtOAc 2:1). The reaction mixture was neutralized at 0 °C by adding a $NaHCO_3$ saturated solution and then extracted with $NaHCO_3$, H_2O and brine. The organic layer was dried over Na_2SO_4 and concentrated. The crude was purified by flash column chromatography on silica gel (PEt:EtOAc 3:1) to give 27 mg of **40** (0.0578 mmol) as a colourless oil and 32 mg of **41** (R_f = 0.40 PEt:EtOAc 1:1, 0.0685 mmol) as a white solid with an overall yield of 25%.

We synthesized **41** following different procedures (*Routes I and II*) with respect to that reported in literature [12]. The characterization data for **41** are in accordance with that reported in literature [12].

40: R_f = 0.44 (PEt:EtOAc 1:1). $[\alpha]_D^{24}$ = + 71.2 (c = 1, $CHCl_3$). ^{1}H-NMR (400 MHz, $CDCl_3$) δ ppm: 5.47 (t, J = 9.8 Hz, 1H, H-3), 5.09-5.01 (m, 2H, H-1, H-4), 4.84 (dd, J = 3.7, 10.2 Hz, 1H, H-2), 4.25 (dd, J = 4.5, 12.4 Hz, 1H, Ha-6), 4.08 (dd, J = 2.0, 12.3 Hz, 1H, Hb-6), 4.03-3.96 (m, 1H, H-5), 3.68 (dt, J = 6.4, 9.8 Hz, 1H, Ha-7), 3.54 (t, J = 6.6 Hz, 2H, H-12), 3.41 (dt, J = 6.5, 9.8 Hz, 1H, Hb-7), 2.09 (s, 3H, OAc), 2.05 (s, 3H, OAc), 2.02 (s, 3H, OAc), 2.01 (s, 3H, OAc), 1.78 (quint, J = 6.7 Hz, 2H, H-11), 1.61 (quint, J = 6.6 Hz, 2H, H-8), 1.51-1.42 (m, 2H, H-10), 1.42-1.33 (m, 2H, H-9). ^{13}C-NMR (100 MHz, $CDCl_3$) δ ppm: 170.8 (s, 1C, C=O), 170.3 (s, 2C, C=O), 169.8 (s, 1C, C=O), 110.1 (interference), 95.8 (d, 1C, C-1), 71.0 (d, 1C, C-2), 70.3 (d, 1C, C-3), 68.7 (t, 1C, C-7), 68.6 (d, 1C, C-4), 67.3 (d, 1C, C-5), 62.1 (t, 1C, C-6), 45.1 (t, 1C, C-12), 32.6 (t, 1C, C-11), 29.2 (t, 1C, C-8), 26.7 (t, 1C, C-10), 25.5 (t, 1C, C-9), 20.9 (q, 1C, OAc), 20.9 (q, 1C, OAc), 20.8 (q, 1C, OAc), 20.8 (q, 1C, OAc). IR ($CHCl_3$): $\tilde{\nu}$ = 3691, 3556, 3033, 2960, 2942, 2865, 1749, 1432, 1369, 1239, 1203, 1168, 1038 cm^{-1}. MS-ESI (m/z, %) = 489.33 (100) $[M+Na]^+$. $C_{20}H_{31}ClO_{10}$ (466.91): calcd C, 51.45; H, 6.69; Cl, 7.59; found C, 51.65; H, 6.63, Cl, 7.41.

Synthesis of (6-Chlorohexyl)-2,3,4,6-tetra-*O*-acetyl-β-D-glucopyranoside **41** (*Route II*)

To a solution of compound **43** [13] (305 mg, 0.619 mmol) and 6-chlorohexanol (265 µl, 1.98 mmol) in dry DCM (8 mL), $BF_3{\cdot}Et_2O$ (90 µl, 0.732 mmol) was added dropwise at – 78 °C. The reaction mixture was allowed to warm to room temperature and stirred at room temperature for 18 h until the disappearance of **43** was attested by a TLC control (Hexane:EtOAc 1:1). The mixture was filtered through Celite® and then washed with aqueous $NaHCO_3$ and brine. The combined organic layers were dried on Na_2SO_4, concentrated and the crude mixture was purified by flash column chromatography on silica gel (Hexane:EtOAc 3:1) to afford 178 mg of pure **41** (0.381 mmol, 62%) as a white solid.

41: R_f = 0.2 (Hexane:EtOAc 3:1). M.p. 97.2-98.8 °C. $[\alpha]_D^{24}$ = - 11.38 (c = 0.8, $CHCl_3$). ^{1}H-NMR (400 MHz, $CDCl_3$) δ ppm: 5.19 (t, J = 9.5 Hz, 1H, H-3), 5.07 (t, J = 9.7 Hz, 1H, H-4), 4.97 (t, J = 8.2 Hz, 1H, H-2), 4.48 (d, J = 8.0 Hz, 1H, H-1), 4.25 (dd, J = 4.7, 12.3 Hz, 1H, Ha-6), 4.12 (dd, J = 2.3, 12.3 Hz, 1H, Hb-6), 3.86 (dt, J = 6.2, 9.6 Hz, 1H, Ha-7), 3.70-3.65 (m, 1H, H-5), 3.51 (t, J = 6.6 Hz, 2H, H-12), 3.47 (dt, J = 6.7, 9.6 Hz, 1H, Hb-

7), 2.07 (s, 3H, OAc), 2.03 (s, 3H, OAc), 2.01 (s, 3H, OAc), 1.99 (s, 3H, OAc), 1.75 (quint, J = 6.7 Hz, 2H, H-11), 1.64-1.53 (m, 2H, H-8), 1.43 (quint, J = 7.5 Hz, 2H, H-10), 1.38-1.30 (m, 2H, H-9). ^{13}C-NMR (100 MHz, $CDCl_3$) δ ppm: 170.8 (s, 1C, C=O), 170.4 (s, 1C, C=O), 169.5 (s, 1C, C=O), 169.4 (s, 1C, C=O), 100.9 (d, 1C, C-1), 73.0 (d, 1C, C-3), 71.9 (d, 1C, C-5), 71.5 (d, 1C, C-2), 70.0 (t, 1C, C-7), 68.6 (d, 1C, C-4), 62.1 (t, 1C, C-6), 45.1 (t, 1C, C-12), 32.6 (t, 1C, C-11), 29.3 (t, 1C, C-8), 26.6 (t, 1C, C-10), 25.2 (t, 1C, C-9), 20.9 (q, 1C, OAc), 20.8 (q, 1C, OAc), 20.7 (q, 1C, OAc), 20.7 (q, 1C, OAc). IR ($CHCl_3$): $\tilde{\nu}$ = 3749, 3673, 3649, 3021, 2962, 2855, 1754, 1374, 1261, 1235, 1092, 1040, 938 cm^{-1}. MS-ESI (m/z, %) = 489.17 (100) $[M+Na]^+$. $C_{20}H_{31}ClO_{10}$ (466.91): calcd C, 51.45; H, 6.69; Cl, 7.59; found C, 51.23; H, 6.71, Cl, 7.77.

Synthesis of (6-Azidohexyl)-2,3,4,6-tetra-*O*-acetyl-β-D-glucopyranoside **42**

To a solution of **41** [12] (166 mg, 0.356 mmol) in dry DMF (11 mL), NaN_3 (215 mg, 3.31 mmol) was added, and the mixture was stirred for 4 days at 55 °C. Then, the reaction mixture was diluted with $CHCl_3$ and extracted with $NaHCO_3$ saturated solution, H_2O and brine. The organic layer was dried over Na_2SO_4, concentrated and the crude was purified by flash column chromatography on silica gel (Hexane:EtOAc 2:1) to give 134 mg of **42** (0.283 mmol, 79%) as a white solid.

42: R_f = 0.27 (Hexane:EtOAc 2:1). M. p. = 67.2-69.1 °C. $[\alpha]_D^{24}$ = - 10.36 (c = 0.55, $CHCl_3$). ^{1}H-NMR (400 MHz, $CDCl_3$) δ ppm: 5.20 (t, J = 9.5 Hz, 1H, H-3), 5.08 (t, J = 9.8 Hz, 1H, H-4), 4.98 (t, J = 8.4 Hz, 1H, H-2), 4.48 (d, J = 7.8 Hz, 1H, H-1), 4.26 (dd, J = 4.7, 12.2 Hz, 1H, Ha-6), 4.13 (dd, J = 2.1, 12.2 Hz, 1H, Hb-6), 3.87 (dt, J = 6.3, 9.5 Hz, 1H, Ha-7), 3.71-3.66 (m, 1H, H-5), 3.47 (dt, J = 6.6, 9.5 Hz, 1H, Hb-7), 3.25 (t, J = 6.9 Hz, 2H, H-12), 2.08 (s, 3H, OAc), 2.03 (s, 3H, OAc), 2.02 (s, 3H, OAc), 2.00 (s, 3H, OAc), 1.63-1.53 (m, 4H, H-8, H-11), 1.41-1.31 (m, 4H, H-9, H-10). ^{13}C-NMR (100 MHz, $CDCl_3$) δ ppm: 170.7 (s, 1C, C=O), 170.3 (s, 1C, C=O), 169.5 (s, 1C, C=O), 169.3 (s, 1C, C=O), 100.9 (d, 1C, C-1), 72.9 (d, 1C, C-3), 71.8 (d, 1C, C-5), 71.4 (d, 1C, C-2), 70.0 (t, 1C, C-12), 68.5 (d, 1C, C-4), 62.1 (t, 1C, C-6), 51.4 (t, 1C, C-7), 29.3, 28.8 (t, 2C, C-8, C-11), 26.5, 25.5 (t, 2C, C-9, C-10), 20.8 (q, 1C, OAc), 20.7 (q, 1C, OAc), 20.7 (q, 1C, OAc), 20.7 (q, 1C, OAc). IR ($CHCl_3$): $\tilde{\nu}$ = 3748, 3690, 3650, 3032, 2942, 2865, 1757, 1455, 1433, 1368, 1237, 1198, 1171, 1042 cm^{-1}. MS-ESI (m/z, %) = 474.50 (100) $[M+H]^+$. $C_{20}H_{31}N_3O_{10}$ (473.47): calcd C, 50.73; H, 6.60; N, 8.87; found C, 50.90; H, 6.78; N, 9.00.

Synthesis of compound **49**

To a solution 6-azidohexan-1-ol [14] (184 mg, 1.285 mmol) in a 1:1 of hexane:Et_2O mixture (0.522 mL), 3,4-dihydro-2H-pyran (DHP, 141 µl, 1.542 mmol), and Amberlyst 15 (31 mg) were added, and the reaction mixture was stirred at room temperature for 3 hours. After this time, a TLC control attested the disappearance of the starting material 6-azidohexan-1-ol (Hexane:Et_2O 1:1). The mixture was filtered through Celite®, the solvent was removed under vacuum and the crude was purified by flash column chromatography on silica gel (Hexane:Et_2O 10:1) to give 260 mg of **49** (1.144 mmol, 89% yield) as a colourless oil.

49: R_f = 0.33 (Hexane:Et_2O 10:1). ^{1}H-NMR (400 MHz, $CDCl_3$) δ ppm: 4.57 (pt, 1H, H-1), 3.86 (ddd, J = 10.9, 7.3, 3.1 Hz, 1H, Ha-5), 3.74 (dt, J = 9.6, 6.7 Hz, 1H, Ha-6), 3.52 – 3.47 (m, 1H, Hb-5), 3.39 (dt, J = 13, 6.7 Hz, 1H, Hb-6), 3.26 (t, J=6.9, 2H, H-11), 1.85 – 1.78 (m, 1H, Ha-3), 1.75 -1.68 (m, 1H, Ha-2), 1.61 – 1.39 (m, 12H, Hb-3, Hb-2, H-4, H-7, H-8, H-9, H-10). ^{13}C-NMR (100 MHz, $CDCl_3$) δ ppm: 99.1 (d, 1C, C-1), 67.6 (t, 1C, C-6), 62.5 (t, 1C, C-5), 51.6 (t, 1C, C-11), 30.9 (t, 1C, C-2), 29.8, 28.9, 26.7, 26.0, 25.6 (t, 5C, C-4, C-7, C-8, C-9, C-10), 19.9 (t, 1C, C-3). IR (neat): $\tilde{\nu}$ = 2937, 2862, 2091, 1121, 1074, 1028. MS-ESI (m/z, %) = 250.08 (100) $[M+Na]^+$. $C_{11}H_{31}N_3O_2$ (227.31): calcd C, 58.12; H, 9.31; N, 18.49; found C, 58.10; H, 8.98; N, 18.48.

General procedure for CuAAC reaction to synthesize multivalent compounds **44** and **46**

Azide **42** (appropriate equivalents), $CuSO_4$ (0.3 equiv) and sodium ascorbate (0.6 equiv) were added to a solution of alkyne **10** [4] or **12** [10] (1 equiv.) in THF (2 mL) and milliQ water (1 mL). The reaction mixture was stirred in a MW reactor at 80°C for 45 min until TLC analysis (PEt:EtOAc 1:1) showed the disappearance of the starting material (R_f =0.67) and formation of the desired product. After filtration through Celite®, the

solvent was removed under reduced pressure and the crude was purified by FCC to obtain the multivalent adducts **44** and **46**

Synthesis of tetravalent compound **44**

Application of the general procedure to scaffold **10** (6 mg, 0.0208 mmol) with 4.3 equiv. of **42** (42 mg, 0.0887 mmol), $CuSO_4$ (1 mg, 0.00627 mmol) and sodium ascorbate (3 mg, 0.0151 mmol) gave, after purification by FCC on silica gel (gradient eluent from DCM:MeOH 40:1 to 20:1), 38 mg of **44** (0.0174 mmol, 79%) as a colourless oil.

44: R_f = 0.31 (DCM:MeOH 40:1). $[\alpha]_D^{28}$ = - 13.9 (c = 1, $CHCl_3$). ^{1}H-NMR (400 MHz, $CDCl_3$) δ ppm: 7.55 (s, 4H, triazole), 5.17 (m, 4H, H-3), 5.05 (m, 4H, H-4), 4.94 (m, 4H, H-2), 4.52 (s, 8H, O*CH₂*Triazole), 4.46 (m, 4H, H-1), 4.30 (t, J = 6.6 Hz, 8H, H-12), 4.27-4.20 (m, 4H, Ha-6), 4.13-4.07 (m, 4H, Hb-6), 3.87-3.79 (m, 4H, Ha-7), 3.71-3.63 (m, 4H, H-5), 3.47-3.40 (m, 12H, Hb-7, CCH_2O), 2.06-2.04 (m, 12H, OAc), 2.01-1.99 (m, 24H, OAc), 1.99-1.96 (m, 12H, OAc), 1.91-1.82 (m, 8H, H-11), 1.61-1.50 (m, 8H, H-8), 1.40-1.26 (m, 16H, H-9, H-10). ^{13}C-NMR (100 MHz, $CDCl_3$) δ ppm: 170.7 (s, 4C, C=O), 170.3 (s, 4C, C=O), 169.5 (s, 4C, C=O), 169.4 (s, 4C, C=O), 145.3 (s, 4C, triazole), 122.5 (d, 4C, triazole), 110.6, 110.1 (interference), 100.9 (d, 4C, C-1), 72.9 (d, 4C, C-3), 71.8 (d, 4C, C-5), 71.4 (d, 4C, C-2), 70.0 (t, 4C, C-7), 69.2 (t, 4C, CCH_2O), 68.5 (d, 4C, C-4), 65.1 (t, 4C, OCH_2Triazole), 62.0 (t, 4C, C-6), 50.2 (t, 4C, C-12), 45.3 (s, 1C, $\underline{C}(CH_2O)_4$-), 30.3 (t, 4C, C-11), 29.3 (t, 4C, C-8), 26.3, 25.4 (t, 8C, C-9, C-10), 20.9 (q, 4C, OAc), 20.8 (q, 4C, OAc), 20.8 (q, 4C, OAc), 20.7 (q, 4C, OAc). IR ($CHCl_3$): $\tilde{\nu}$ = 3672, 3031, 2944, 2865, 1756, 1459, 1369, 1236, 1197, 1171, 1136, 1042 cm^{-1}. HRESIMS calcd for $C_{97}H_{144}N_{12}O_{44}$ $[M+2H]^{2+}$: 1091.97892; found 1091.97693.

Synthesis of nonavalent compound **46**

Application of the general procedure to scaffold **12** (16 mg, 0.0188 mmol) with 9 equiv. of **42** (80 mg, 0.169 mmol), $CuSO_4$ (1 mg, 0.00627 mmol) and sodium ascorbate (2 mg, 0.0101 mmol) gave, after purification by FCC on silica gel (DCM:MeOH 20:1) to give 84 mg of **46** (0.0164 mmol, 91%) as a white waxy solid.

46: R_f = 0.22 (DCM:MeOH 20:1). $[\alpha]_D^{26}$ = - 7.83 (c = 0.60, $CHCl_3$). ^{1}H-NMR (400 MHz, $CDCl_3$) δ ppm: 8.26 (s, 3H, Ar), 7.61 (s, 9H, triazole), 6.99 (br s, 3H, NH), 5.18 (m, 9H, H-3), 5.05 (m, 9H, H-4), 4.94 (m, 9H, H-2), 4.58 (s, 18H, O*CH₂*Triazole), 4.47 (m, 9H, H-1), 4.35-4.20 (m, 27H, Ha-6, H-12), 4.11 (m, 9H, Hb-6), 3.89 (s, 18H, C*CH₂*O), 3.87-3.79 (m, 9H, Ha-7), 3.68 (m, 9H, H-5), 3.48-3.39 (m, 9H, Hb-7), 2.07-2.05 (m, 27H, OAc), 2.01 (m, 54H, OAc), 1.99-1.97 (m, 27H, OAc), 1.90-1.81 (m, 18H, H-11), 1.60-1.48 (m, 18H, H-8), 1.37-1.26 (m, 36H, H-9, H-10). ^{13}C-NMR (100 MHz, $CDCl_3$) δ ppm: 170.8 (s, 9C, $CH_3\underline{C}$=O), 170.4 (s, 9C, $CH_3\underline{C}$=O), 169.6 (s, 9C, $CH_3\underline{C}$=O), 169.4 (s, 9C, $CH_3\underline{C}$=O), 166.2 (s, 3C, HN$\underline{C}$=O), 144.7 (s, 9C, triazole), 135.8 (s, 3C, Ar), 128.6 (d, 3C, Ar), 122.9 (d, 9C, triazole), 110.1 (interference), 100.9 (d, 9C, C-1), 72.9 (d, 9C, C-3), 71.8 (d, 9C, C-5), 71.4 (d, 9C, C-2), 70.0 (t, 9C, C-7), 68.8 (t, 9C, C*CH₂*O), 68.5 (d, 9C, C-4), 65.0 (t, 9C, OCH_2Triazole), 62.1 (t, 9C, C-6), 60.7 (s, 3C, HN$\underline{C}(CH_2O)_3$-), 50.3 (t, 9C, C-12), 30.3 (t, 9C, C-11), 29.3 (t, 9C, C-8), 26.3, 25.4 (t, 18C, C-9, C-10), 20.9 (q, 9C, OAc), 20.8 (q, 9C, OAc), 20.7 (q, 18C, OAc). IR ($CDCl_3$): $\tilde{\nu}$ = 3673, 3418, 3032, 3007, 2943, 2865, 1755, 1712, 1669, 1511, 1366, 1237, 1042 cm^{-1}. HRESIMS calcd for $C_{228}H_{330}N_{30}O_{102}$ $[M+3H]^{3+}$: 1708.39477; found 1708.39111.

Synthesis of nonavalent compound **50**

Application of the general procedure to scaffold **12** [10] (13 mg, 0.0151 mmol) with 9.3 equiv. of **49** (32 mg, 0.140 mmol), $CuSO_4$ (1 mg, 0.00450 mmol) and sodium ascorbate (2 mg, 0.0091 mmol) gave, after purification by FCC on silica gel (DCM:MeOH 30:1) to give 20 mg of **50** (0.00688 mmol, 46%) as a white waxy solid.

50: R_f = 0.21 (DCM:MeOH 30:1). $[\alpha]_D^{20}$ = - 1.0 (c = 0.5, $CHCl_3$). ^{1}H-NMR (400 MHz, $CDCl_3$) δ ppm: 8.26 (s, 3H, Ar), 7.61 (s, 9H, Triazole), 6.95 (s, 3H, N*H*), 4.59 (s, 18H, C*H₂*Triazole), 4.53 (pt, 9H, H-1), 4.32 (t, J = 7.3 Hz, 18H, H-11), 3.90 (s, 18H, C*CH₂*O), 3.82 (ddd, J = 10.9, 7.2, 3.0 Hz, 9H, Ha-5), 3.69 (dt, J = 9.5, 7.5 Hz, 9H, Ha-6), 3.49-3.44 (m, 9H, Hb-5), 3.34 (dt, J = 9.5, 6.4 Hz, 9H, Hb-6), 1.93 – 1.85 (m, 18 H, H-10), 1.82 - 1.75

(m, 9H, Ha-3), 1.71, 1.63 (m, 9H, Ha-2), 1.59 – 1.47 (m, 54 H, Hb-2, Hb-3, H-7, H-4), 1.43 – 1.30 (m, 36H, H-9, H-8). ^{13}C-NMR (100 MHz, $CDCl_3$) δ ppm: 166.1 (s, 3C, C=O), 144.8 (s, 9C, Triazole), 135.9 (s, 3C, Ar), 128.6 (d, 3C, Ar), 122.8 (d, 9C, Triazole), 99.0 (d, 9C, C-1), 68.9 (t, 9C, CCH_2O), 67.4 (t, 9C, C-6), 65.0 (t, 9C, OCH_2Triazole), 62.6 (t, 9C, C-5), 60.7 (s, 3C, HNC$(CH_2O)_3$-), 50.38 (t, 9C, C-11), 30.8, 30.4, 29.7, 26.5, 25.9, 25.6, (t, 54C, C-2, C-4, C-7, C-8, C-9, C-10), 19.9 (t, 9C, C-3). IR (neat): $\tilde{\nu}$ = 3134, 2934, 2860, 1134, 1117, 1078, 1026. MS-ESI (m/z, %) = 992.17 (100) $[M+3Na]^{3+}$. $C_{147}H_{240}N_{30}O_{30}$ (2907.72): calcd C, 60.72; H, 8.32; N, 14.45; found C, 60.46; H, 7.95; N, 14.10.

Synthesis of tetravalent compound **45**

To a solution of **44** (35 mg, 0.0160 mmol) in MeOH (6 mL), the ion exchange resin Ambersep 900 OH (500 mg) was added and the suspension was slowly stirred at room temperature for 18 h until a TLC control attested the disappearance of the starting material **44** (DCM:MeOH 10:1). The mixture was filtered, and the solvent was removed under reduced pressure. The crude was purified by size exclusion chromatography, employing Bio-Beads_SX8 resin and eluting with MeOH to afford 21 mg of pure **45** (0.0139 mmol, 88%) as a colourless waxy solid.

45: R_f = 0.00 (DCM:MeOH 10:1). $[\alpha]_D^{26}$ = - 21.2 (c = 1, $CHCl_3$). ^{1}H-NMR (400 MHz, D_2O) δ ppm: 7.87 (s, 4H, triazole), 4.45 (br s, 8H, OCH_2Triazole), 4.38 (d, J = 8.0 Hz, 4H, H-1), 4.33 (t, J = 6.8 Hz, 8H, H-12), 3.89-3.78 (m, 8H, Ha-6, Ha-7), 3.67 (dd, J = 5.7, 12.3 Hz, 4H, Hb-6), 3.61-3.53 (m, 4H, Hb-7), 3.44 (m, 4H, H-3), 3.41-3.36 (m, 4H, H-5), 3.34 (d, J = 9.0 Hz, 4H, H-4), 3.32 (br s, 8H, CCH_2O), 3.21 (m, 4H, H-2), 1.80 (quint, J = 7.0 Hz, 8H, H-11), 1.51 (quint, J = 6.8 Hz, 8H, H-8), 1.28 (m, 8H, H-9), 1.17 (m, 8H, H-10). ^{13}C-NMR (100 MHz, D_2O) δ ppm: 144.0 (s, 4C, triazole), 124.5 (d, 4C, triazole), 102.0 (d, 4C, C-1), 75.8 (d, 4C, C-3), 75.7 (d, 4C, C-5), 73.0 (d, 4C, C-2), 70.1 (t, 4C, C-7), 69.5 (d, 4C, C-4), 67.7 (t, 4C, CCH_2O), 63.3 (t, 4C, OCH_2Triazole), 60.6 (t, 4C, C-6), 50.1 (t, 4C, C-12), 44.3 (s, 1C, C$(CH_2O)_4$-), 29.2 (t, 4C, C-11), 28.4 (t, 4C, C-8), 25.1 (t, 4C, C-10), 24.3 (t, 4C, C-9). HRESIMS calcd for $C_{65}H_{112}N_{12}O_{28}$ $[M+2H]^{2+}$: 755.39273; found 755.39258.

Synthesis of nonavalent compound **47**

Sodium carbonate (3 mg, 0.0283 mmol) was added to a solution of **46** (33 mg, 0.00644 mmol) in MeOH (1.2 mL) and the mixture was stirred at room temperature for 18 hours, until a TLC control attested the disappearance of the starting material **46** (DCM:MeOH:NH_4OH (6%) 10:1:0.1). The mixture was neutralized with a 1% HCl in MeOH solution, filtered and the solvent was removed under vacuum. The crude was purified by size exclusion chromatography, employing Bio-Beads_SX8 resin and eluting with MeOH to afford 14 mg of pure **47** (0.00388 mmol, 61%) as a colourless oil.

47: R_f = 0.00 (DCM:MeOH:NH_4OH (6%) 10:1:0.1). $[\alpha]_D^{27}$ = - 25.2 (c = 0.6, MeOH). ^{1}H-NMR (400 MHz, D_2O) δ ppm: 8.10-8.07 (m, 3H, Ar), 7.92 (s, 9H, triazole), 4.61 (s, 18H, OCH_2Triazole), 4.40 (d, J = 8.0 Hz, 9H, H-1), 4.27 (t, J = 6.5 Hz, 18H, H-12), 3.97-3.78 (m, 36H, Ha-6, Ha-7, CCH_2O), 3.71 (dd, J = 5.4, 12.3 Hz, 9H, Hb-6), 3.60-3.53 (m, 9H, Hb-7), 3.48 (m, 9H, H-3), 3.43-3.39 (m, 9H, H-5), 3.38-3.35 (m, 9H, H-4), 3.25 (m, 9H, H-2), 1.78-1.67 (m, 18H, H-11), 1.55-1.45 (m, 18H, H-8), 1.30-1.22 (m, 18H, H-9), 1.20-1.09 (m, 18H, H-10). ^{13}C-NMR (50 MHz, D_2O) δ ppm: 167.5 (s, 3C, HNC=O), 144.1 (s, 9C, triazole), 135.2 (s, 3C, Ar), 128.9 (d, 3C, Ar), 124.5 (d, 9C, triazole), 102.2 (d, 9C, C-1), 75.9 (d, 18C, C-3, C-5), 73.2 (d, 9C, C-2), 70.2 (t, 9C, C-7), 69.7 (d, 9C, C-4), 67.5 (t, 9C, CCH_2O), 63.7 (t, 9C, OCH_2Triazole), 60.8 (t, 9C, C-6), 50.2 (t, 9C, C-12), 48.9 (s, 3C, HNC$(CH_2O)_3$-), 29.3 (t, 9C, C-11), 28.6 (t, 9C, C-8), 25.3 (t, 9C, C-10), 24.4 (t, 9C, C-9). IR (neat): $\tilde{\nu}$ = 3323, 2922, 2859, 1659, 1553, 1462, 1373, 1076, 1026 cm^{-1}. HRESIMS calcd for $C_{156}H_{258}N_{30}O_{66}$ $[M+3H]^{3+}$: 1203.93354; found 1203.93344.

Synthesis of compound **48**

To a solution of azide **42** (47 mg, 0.0993 mmol) in THF (2 mL) and milliQ water (1 mL), propargyl alcohol (11 μl, 0.189 mmol), sodium ascorbate (12 mg, 0.0606 mmol) and $CuSO_4$ (5 mg, 0.0313 mmol) were added, and the reaction mixture was stirred at room temperature for 40 hours. The mixture was filtered through Celite®, the solvent was removed under vacuum the crude was purified by flash column chromatography on silica gel (DCM:MeOH 25:1) to give 17 mg of **48** (0.0321 mmol, 32% yield, 61% yield calculated on the converted starting material) as a pale-yellow oil and 22 mg of **42**.

48: R_f = 0.21 (DCM:MeOH 30:1). $[\alpha]_D^{20}$ = - 1.0 (c = 0.5, $CHCl_3$). ^{1}H-NMR (400 MHz, $CDCl_3$) δ ppm: 7.53 (s, 1H, triazole), 5.18 (t, J = 9.5 Hz, 1H, H-3), 5.06 (t, J = 9.8 Hz, 1H, H-4), 4.96 (t, J = 8.2 Hz, 1H, H-2), 4.78 (m, 2H, C*H2*OH), 4.46 (d, J = 8.0 Hz, 1H, H-1), 4.33 (t, J = 7.1 Hz, 2H, H-12), 4.28-4.20 (m, 1H, Ha-6), 4.16-4.06 (m, 1H, Hb-6), 3.90-3.76 (m, 1H, Ha-7), 3.72-3.62 (m, 1H, H-5), 3.50-3.39 (m, 1H, Hb-7), 2.90 (br s, 1H, OH), 2.06 (s, 3H, OAc), 2.02 (s, 3H, OAc), 2.01 (s, 3H, OAc), 1.99 (s, 3H, OAc), 1.88 (quint, J = 7.3 Hz, 2H, H-11), 1.63-1.47 (m, 2H, H-8), 1.42-1.25 (m, 4H, H-9, H-10). ^{13}C-NMR (50 MHz, $CDCl_3$) δ ppm: 170.9 (s, 1C, C=O), 170.4 (s, 1C, C=O), 169.6 (s, 1C, C=O), 169.5 (s, 1C, C=O), 147.8 (s, 1C, triazole), 121.7 (d, 1C, triazole), 100.9 (d, 1C, C-1), 72.9 (d, 1C, C-3), 71.8 (d, 1C, C-5), 71.4 (d, 1C, C-2), 69.9 (t, 1C, C-7), 68.5 (d, 1C, C-4), 62.0 (t, 1C, C-6), 56.5 (t, 1C, *C*H2OH), 50.3 (t, 1C, C-12), 30.2 (t, 1C, C-11), 29.2 (t, 1C, C-8), 26.2, 25.4 (t, 2C, C-9, C-10), 20.9 (q, 1C, OAc), 20.8 (q, 2C, OAc), 20.7 (q, 1C, OAc). IR ($CHCl_3$): $\tilde{\nu}$ = 3869, 3742, 3649, 2999, 2947, 2866, 1751, 1541, 1456, 1371, 1238, 1213, 1169, 1042 cm^{-1}. MS-ESI (m/z, %) = 552.24 (100) $[M+Na]^+$, 1080.55 (35) $[2M+Na]^+$. $C_{23}H_{35}N_3O_{11}$ (529.54): calcd C, 52.17; H, 6.66; N, 7.94; found C, 52.30; H, 6.87; N, 8.11.

NMR spectra

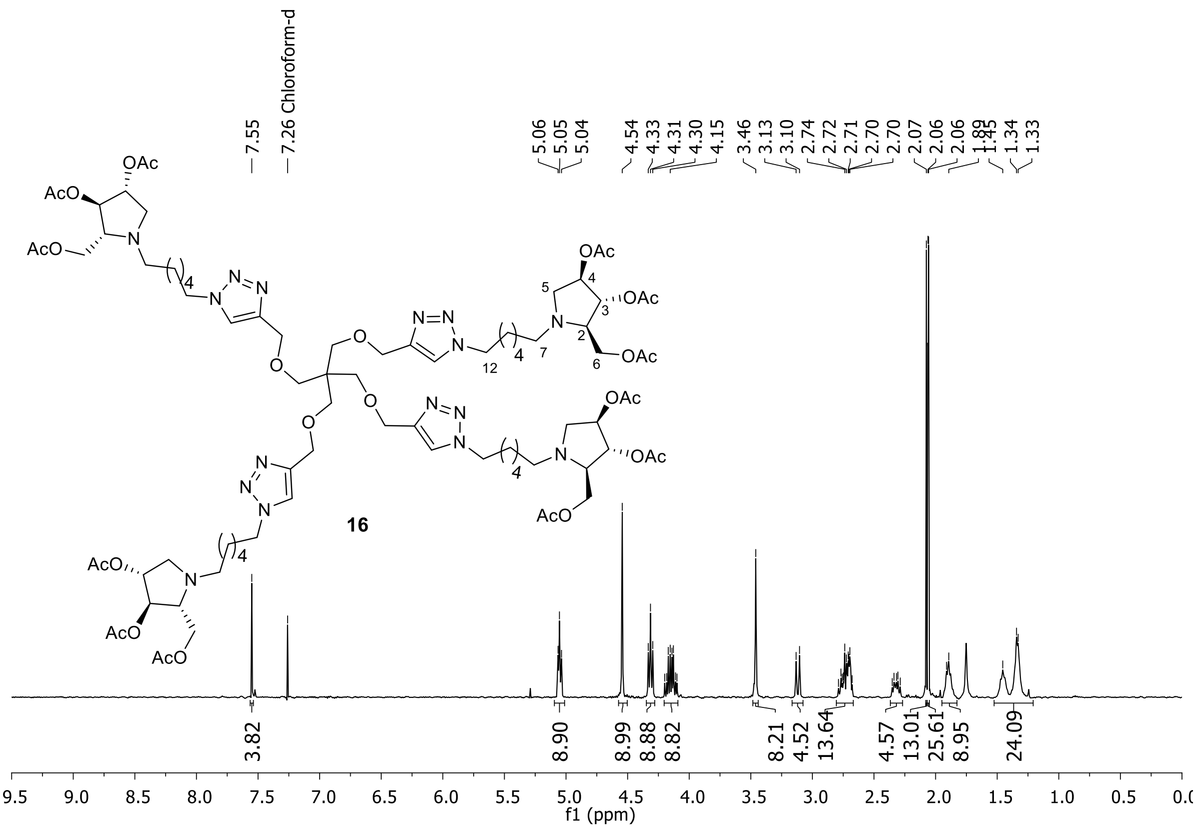

Figure S1: ^{1}H NMR spectrum of 16 (400 MHz, $CDCl_3$)

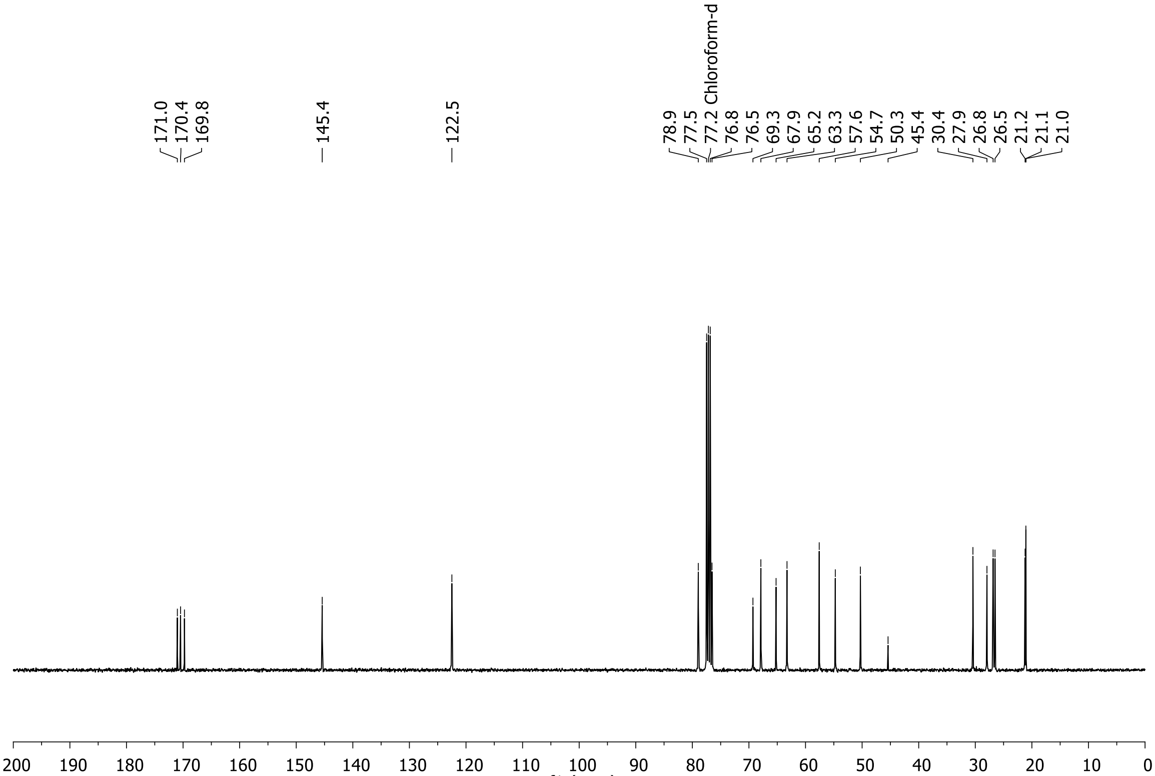

Figure S2: ^{13}C NMR spectrum of 16 (100 MHz, $CDCl_3$).

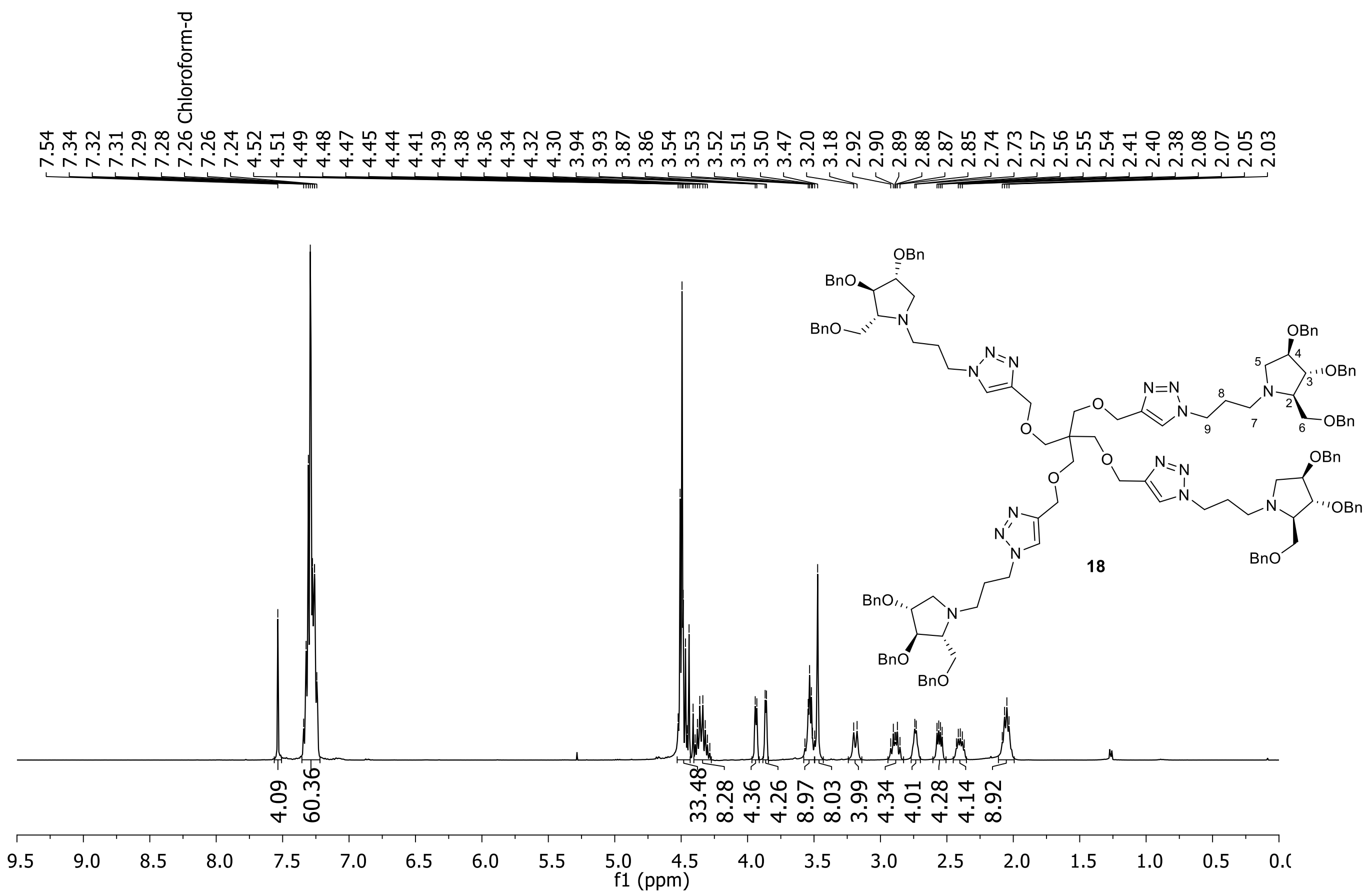

Figure S3: ^{1}H NMR spectrum of 18 (400 MHz, $CDCl_3$)

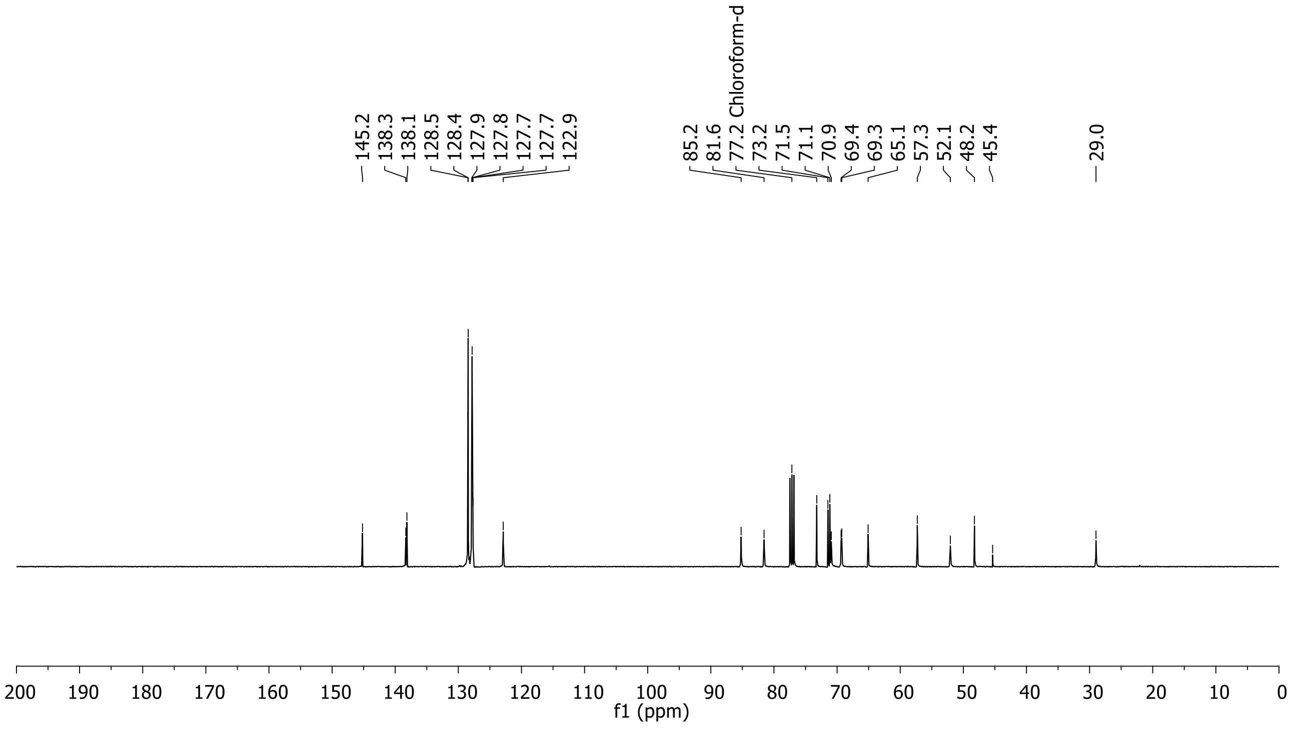

Figure S4: ^{13}C NMR spectrum of 18 (100 MHz, $CDCl_3$).

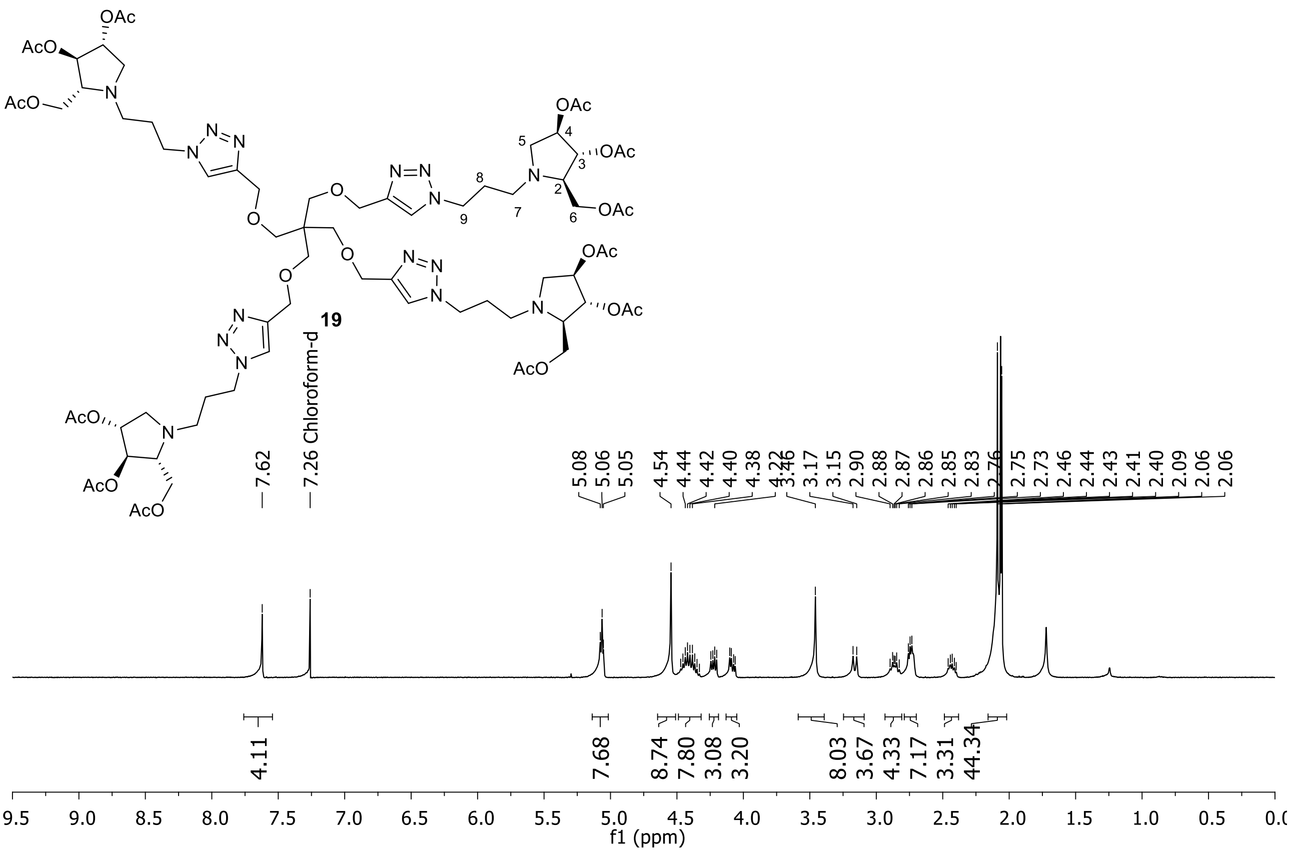

Figure S5: ^{1}H NMR spectrum of 19 (400 MHz, $CDCl_3$)

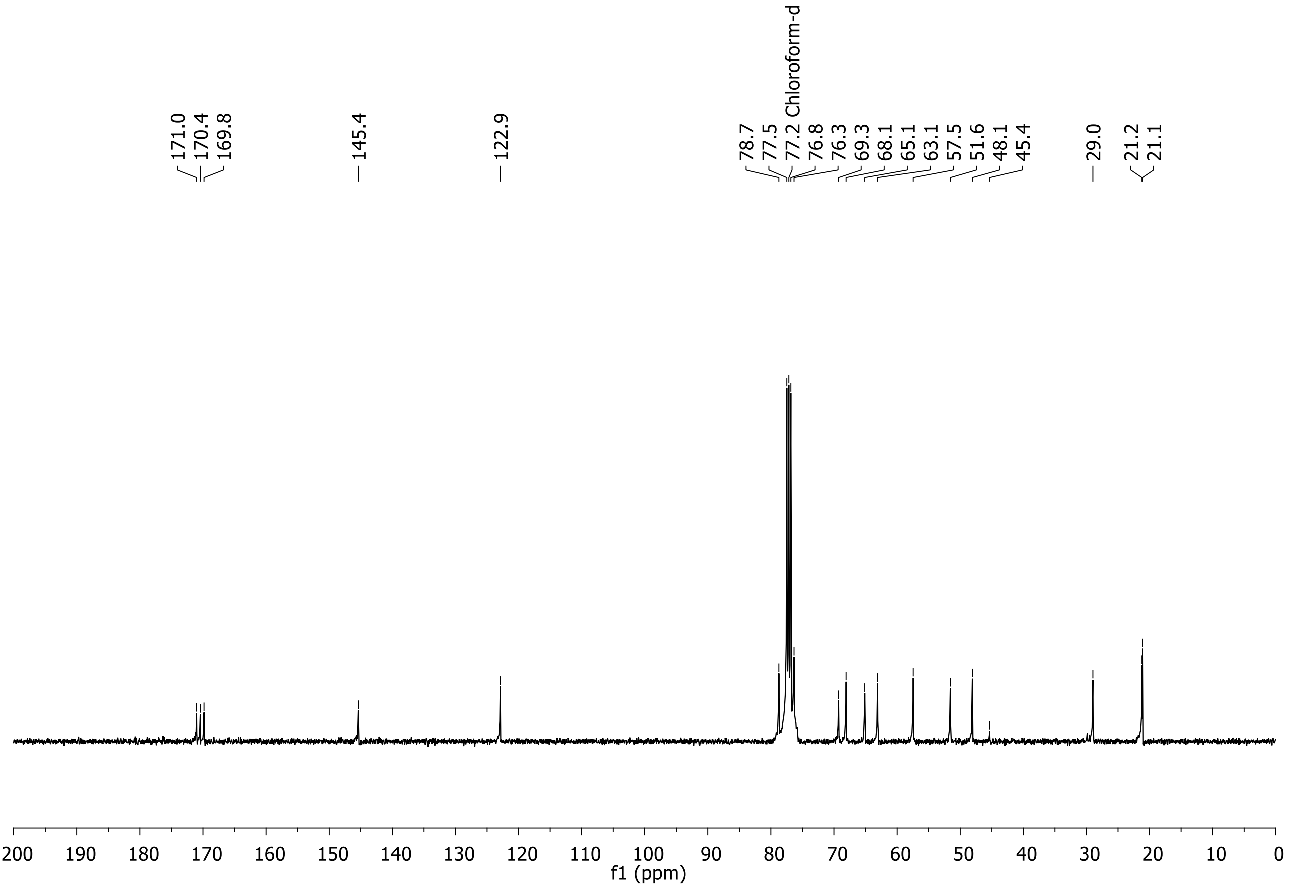

Figure S6: ^{13}C NMR spectrum of 19 (100 MHz, $CDCl_3$).

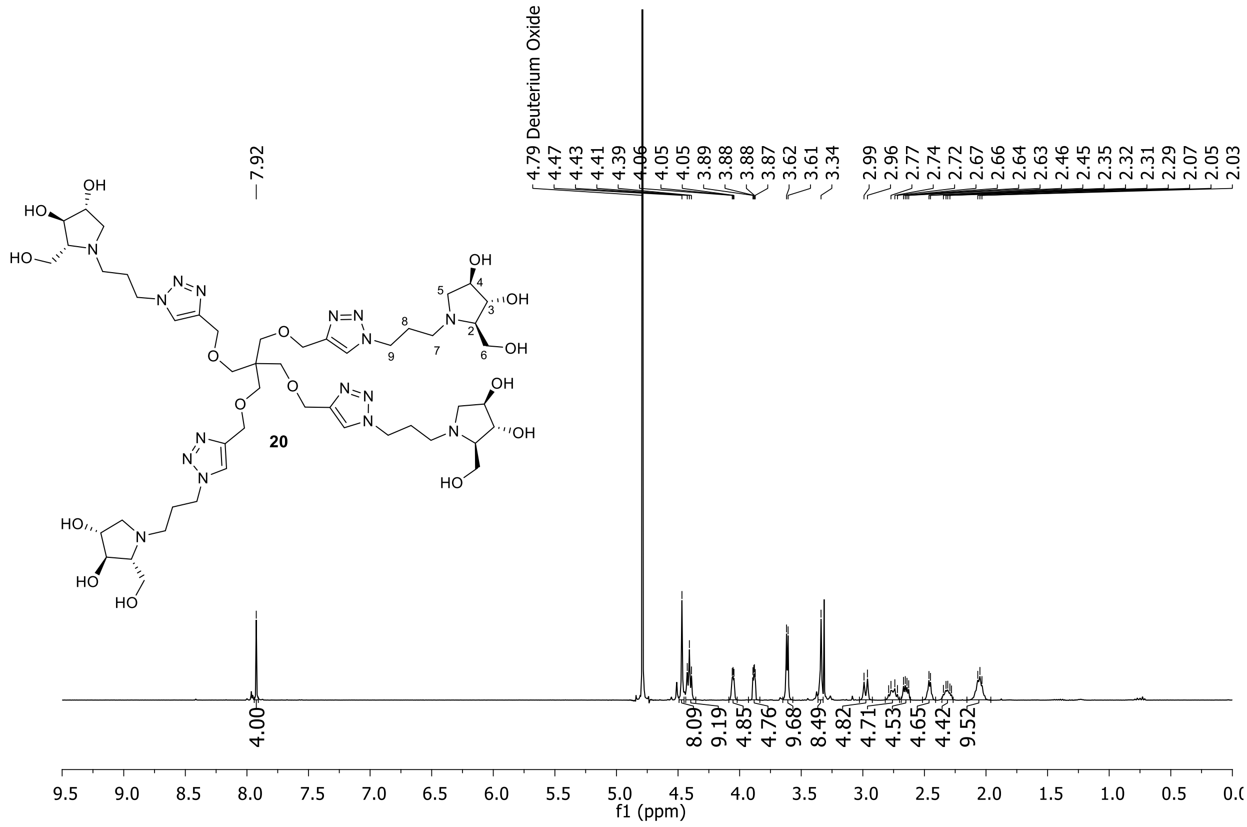

Figure S7: ^{1}H NMR spectrum of 20 (400 MHz, D_2O)

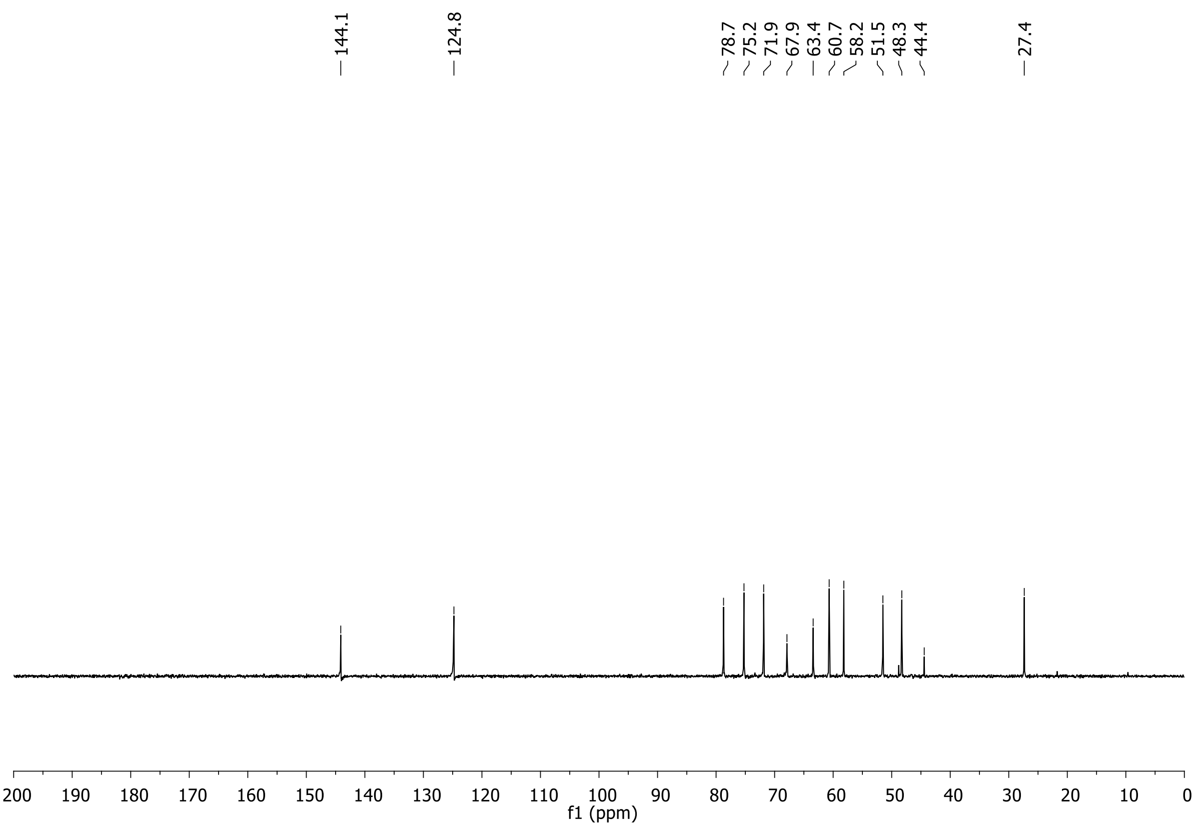

Figure S8: ^{13}C NMR spectrum of 20 (100 MHz, D_2O).

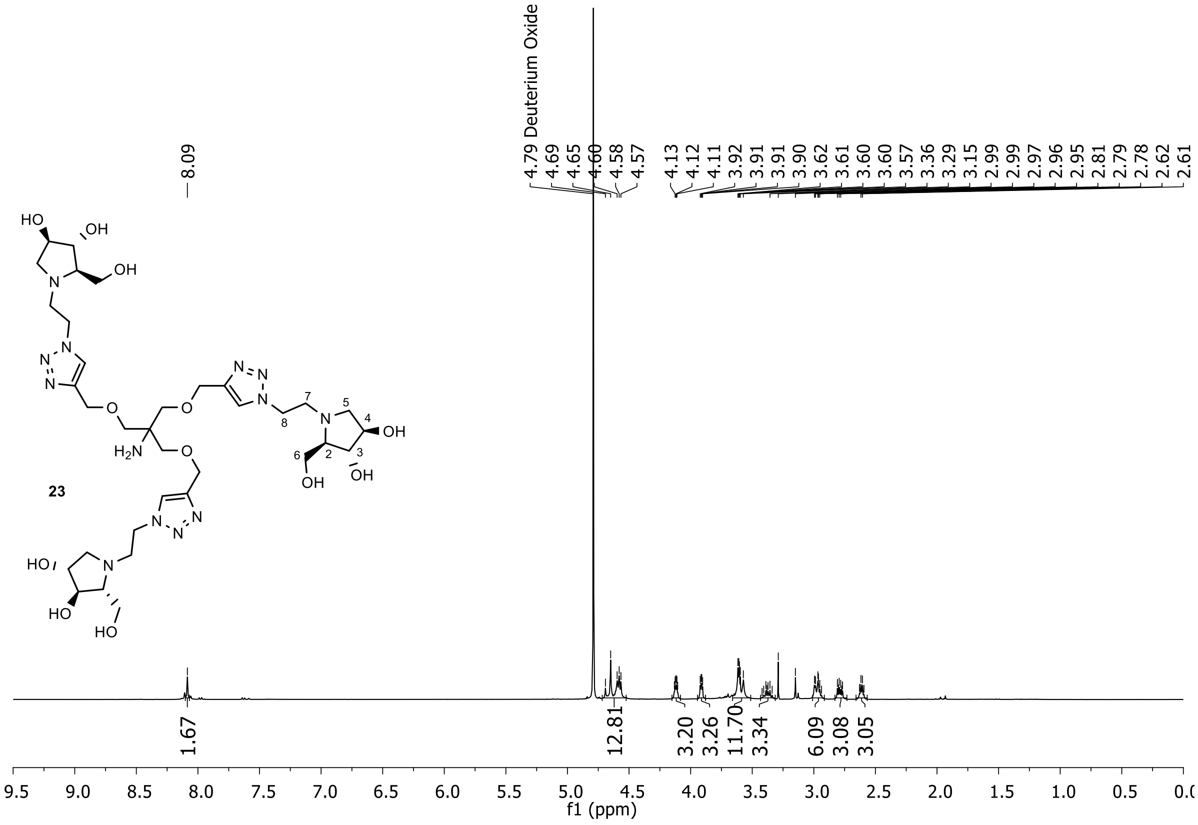

Figure S9: ^{1}H NMR spectrum of 23 (400 MHz, D_2O).

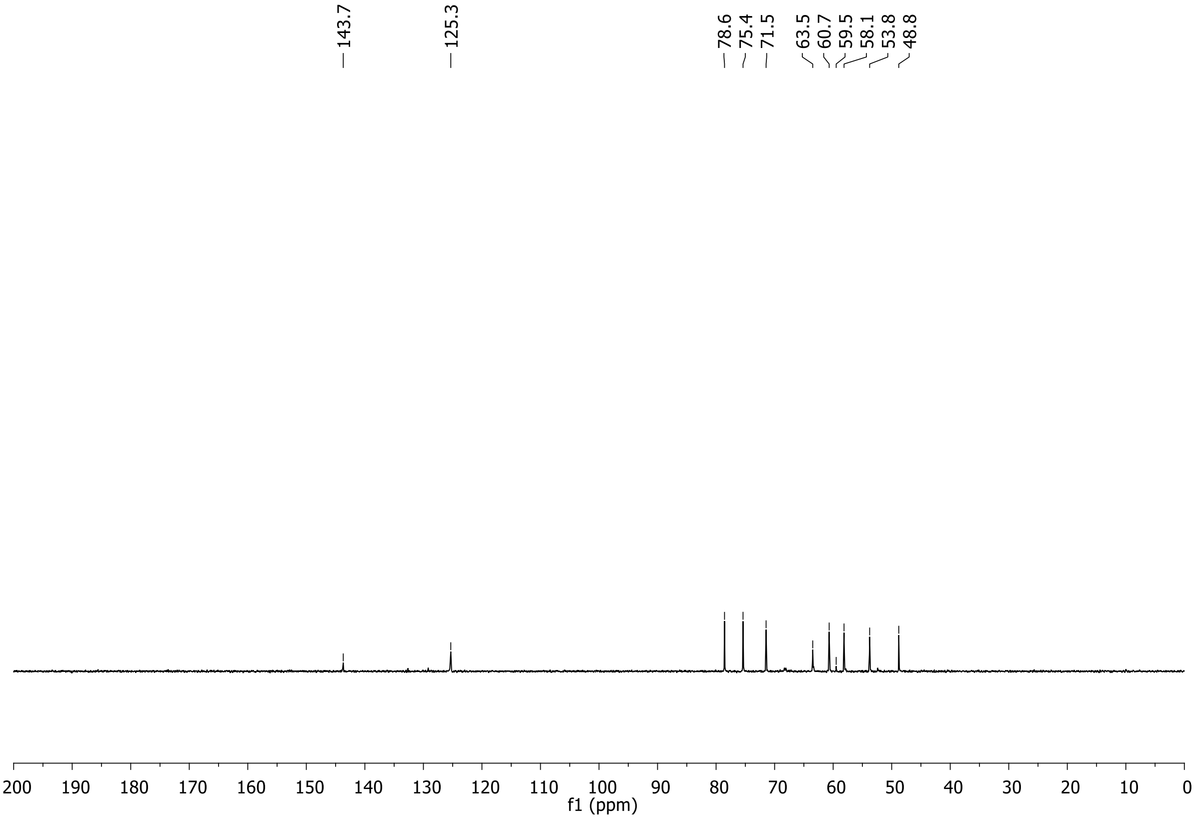

Figure S10: ^{13}C NMR spectrum of 23 (100 MHz, D_2O).

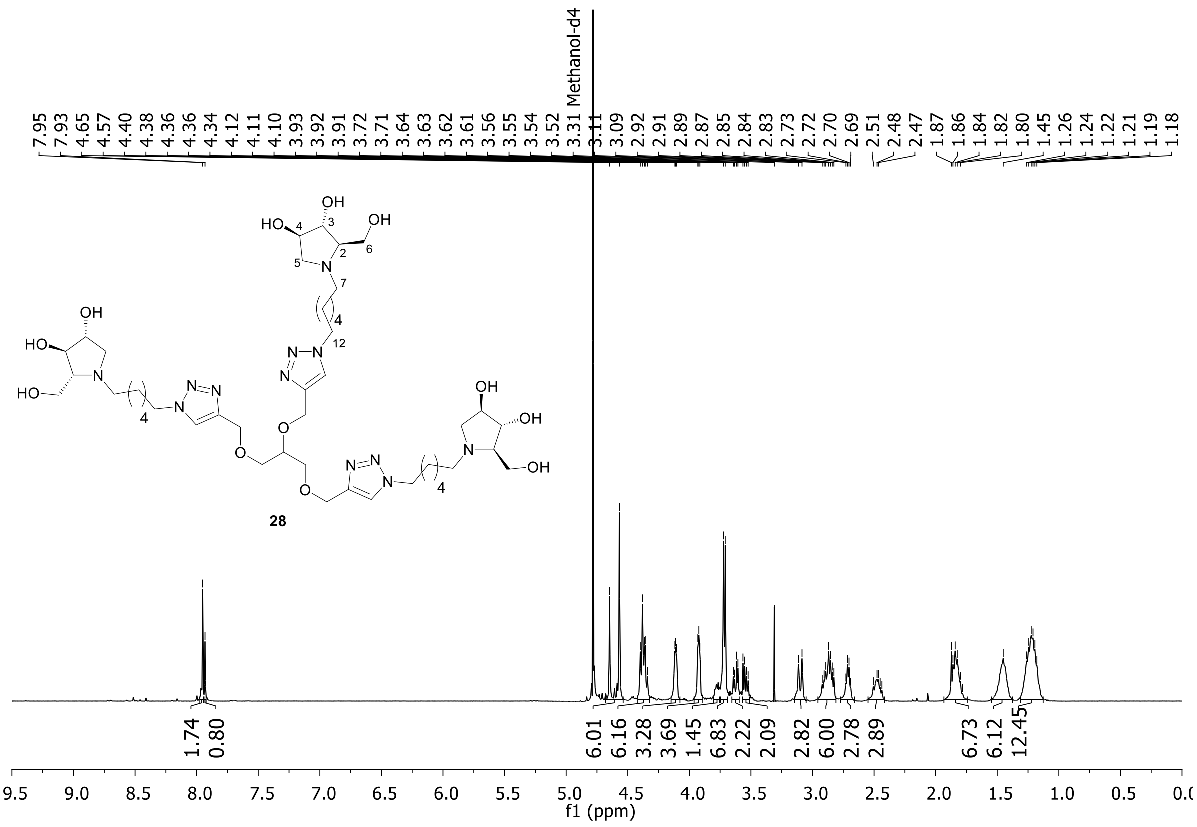

Figure S11: ¹H NMR spectrum of 28 (400 MHz, CD_3OD).

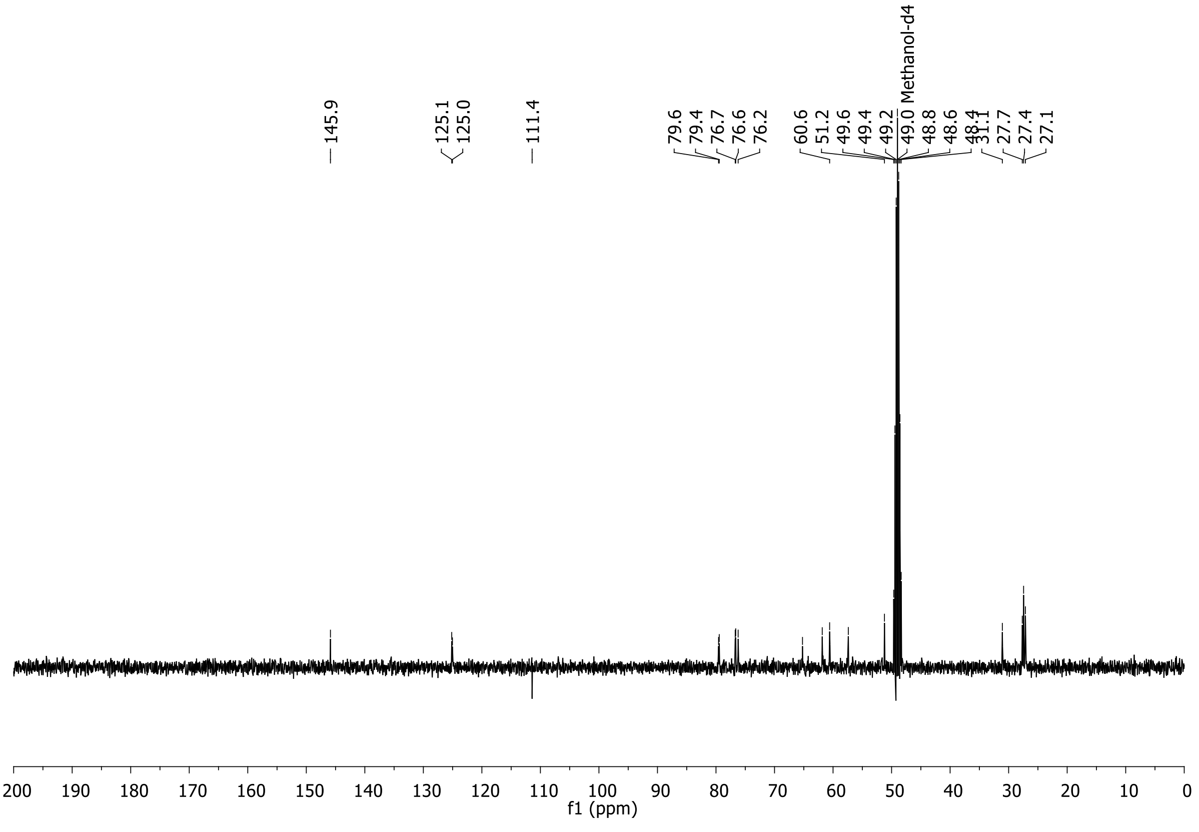

Figure S12: ¹³C NMR spectrum of 28 (100 MHz, CD_3OD).

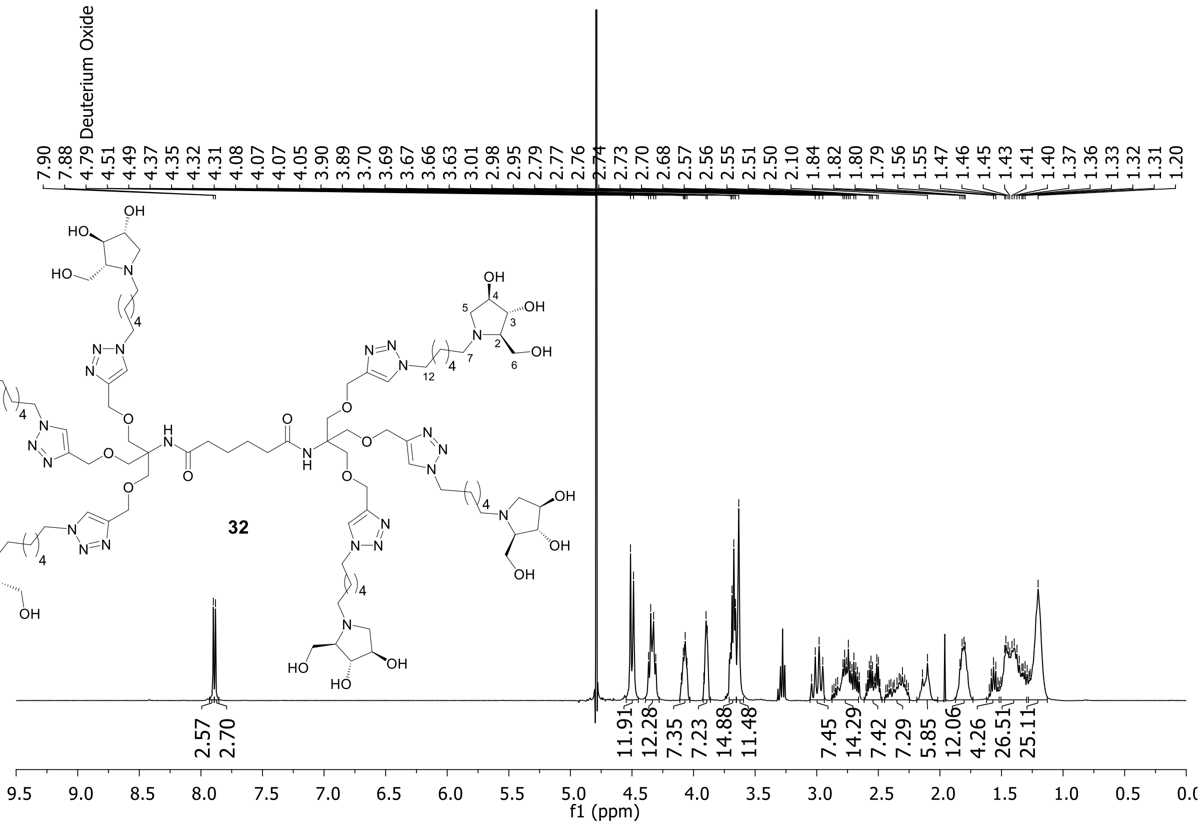

Figure S13: ^{1}H NMR spectrum of 32 (400 MHz, D_2O).

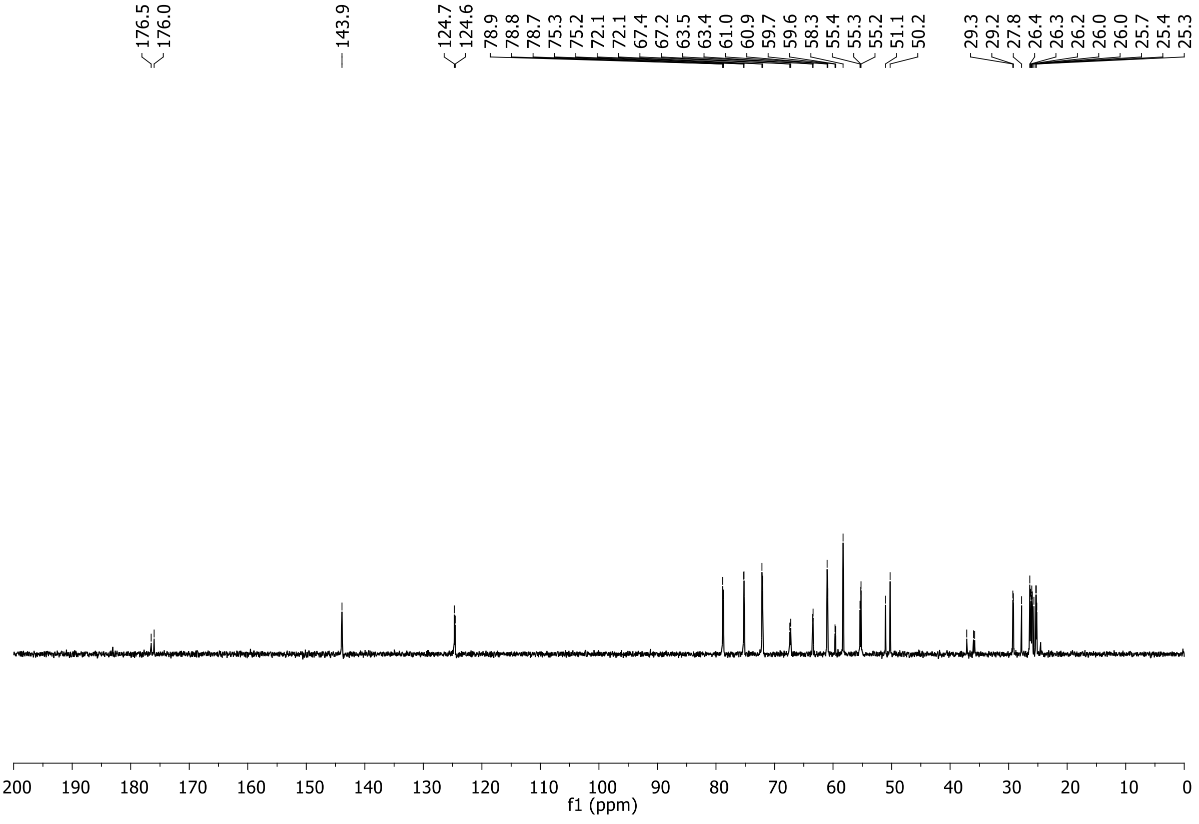

Figure S14: ^{13}C NMR spectrum of 32 (100 MHz, D_2O).

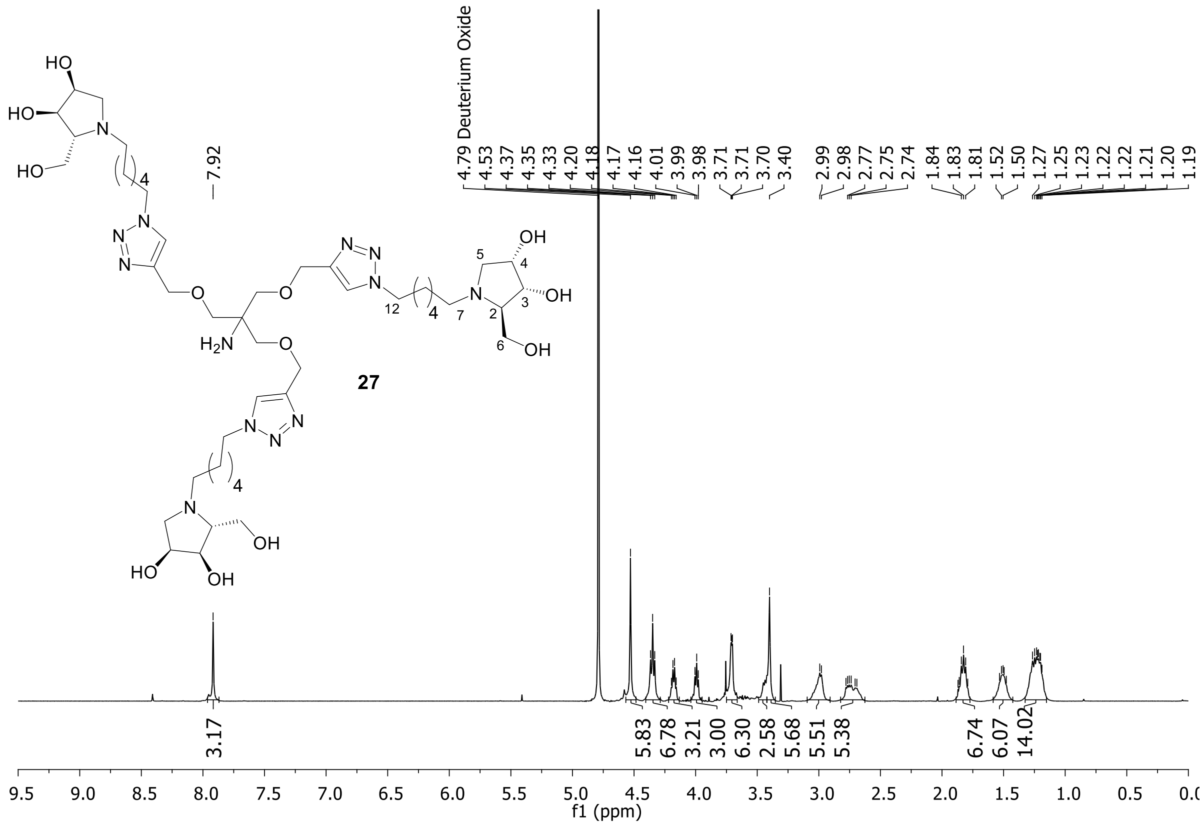

Figure S15: ^{1}H NMR spectrum of 27 (400 MHz, D_2O).

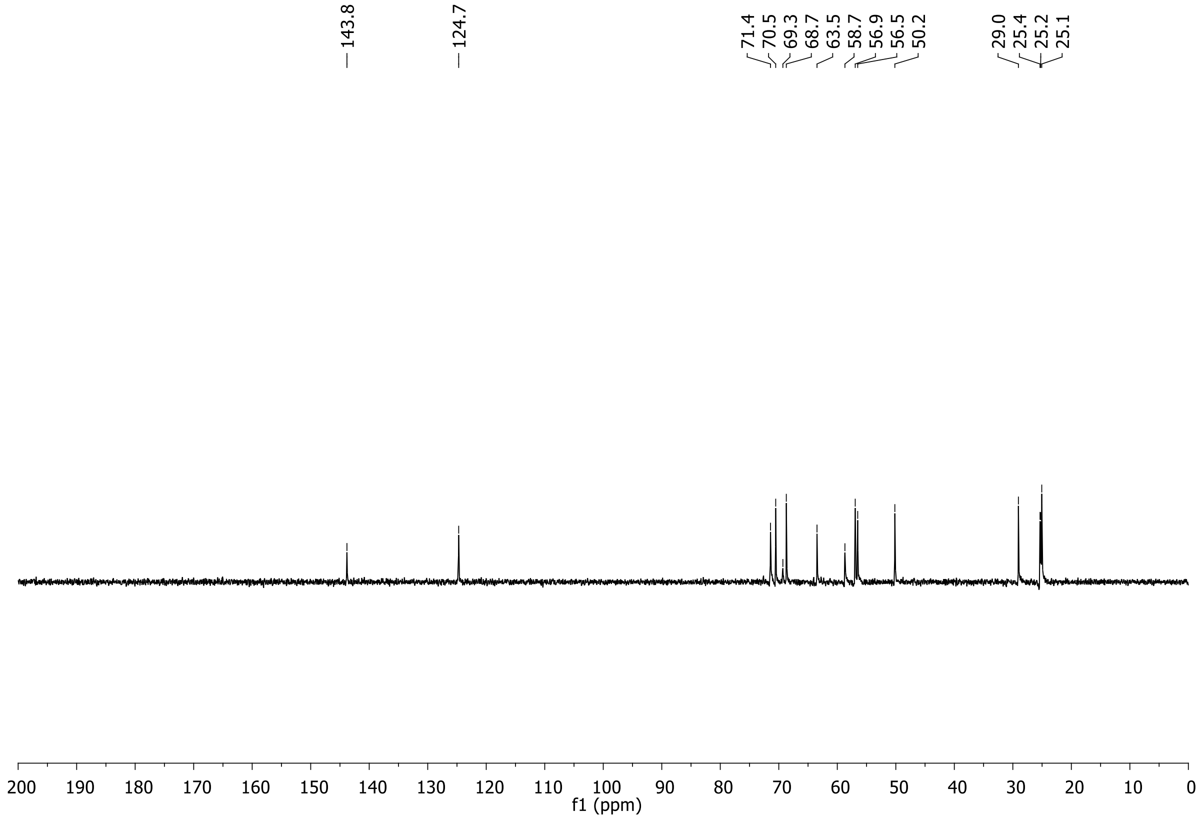

Figure S16: ^{13}C NMR spectrum of 27 (100 MHz, D_2O).

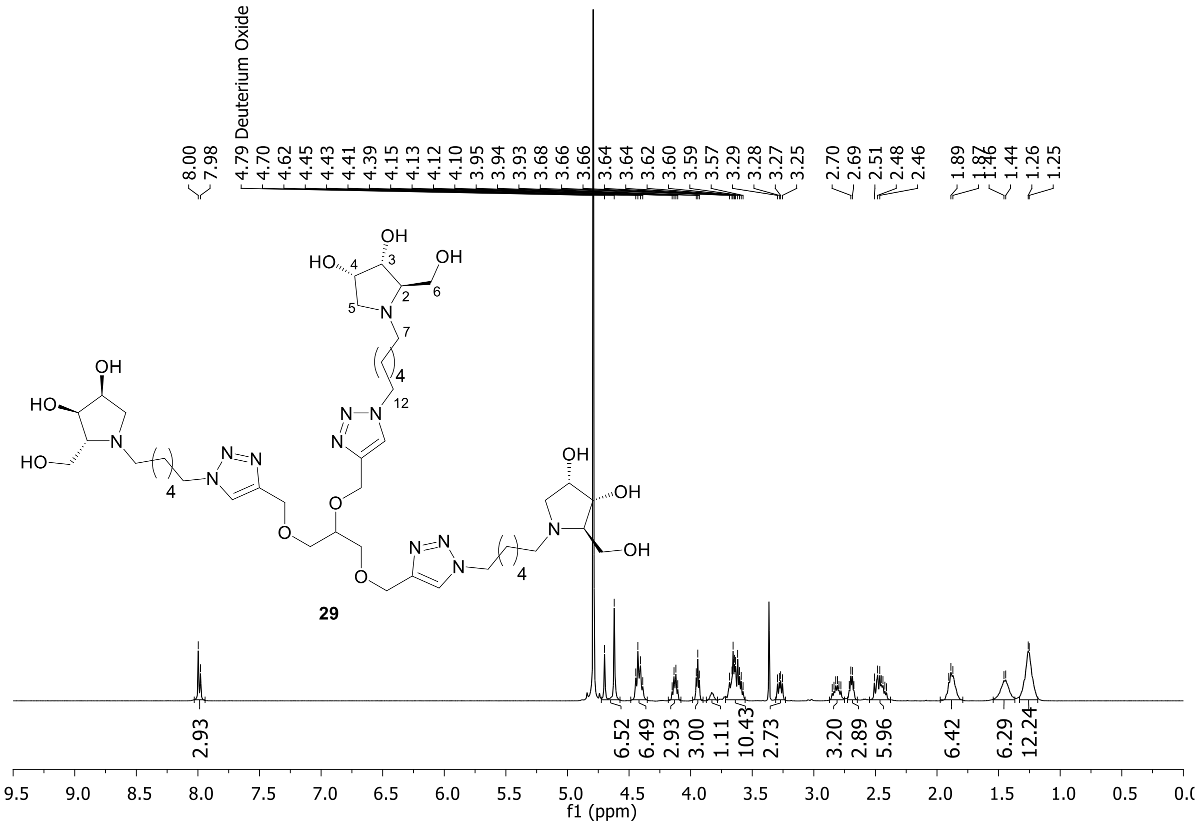

Figure S17: ^{1}H NMR spectrum of 29 (400 MHz, D_2O).

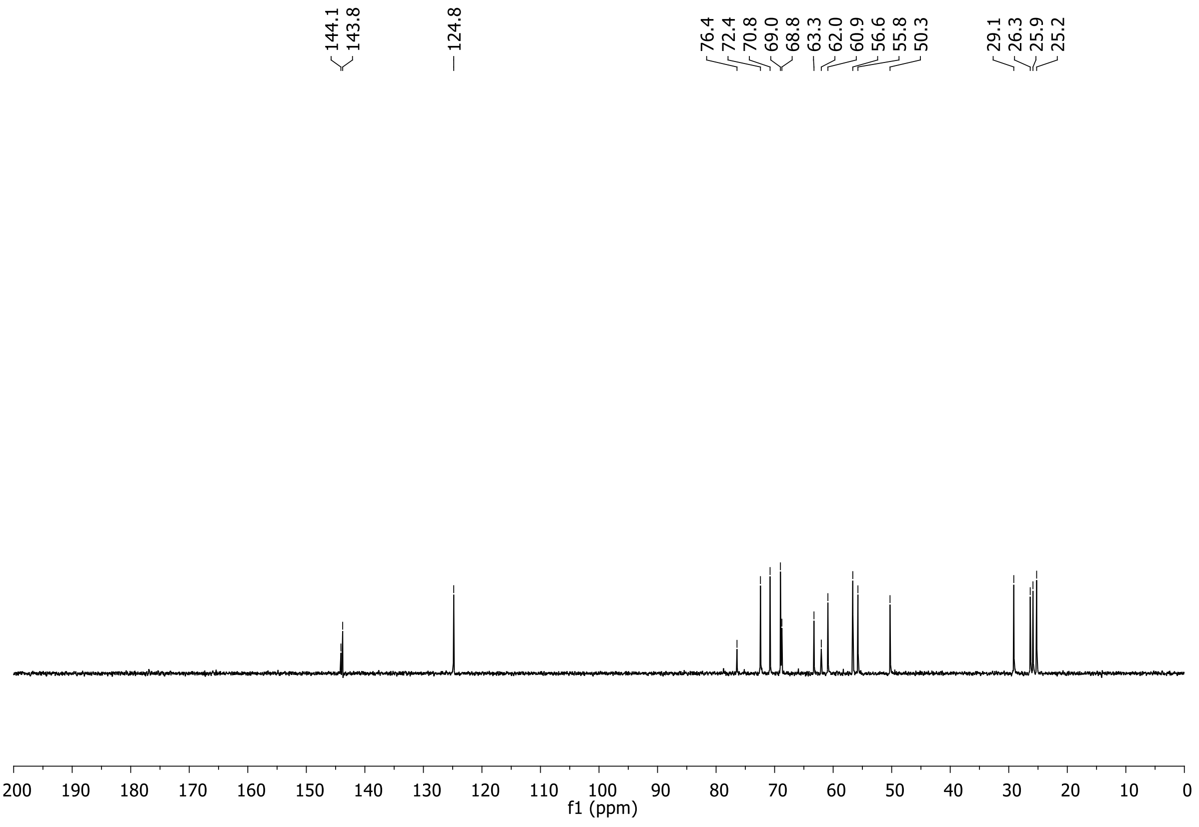

Figure S18: ^{13}C NMR spectrum of 29 (100 MHz, D_2O).

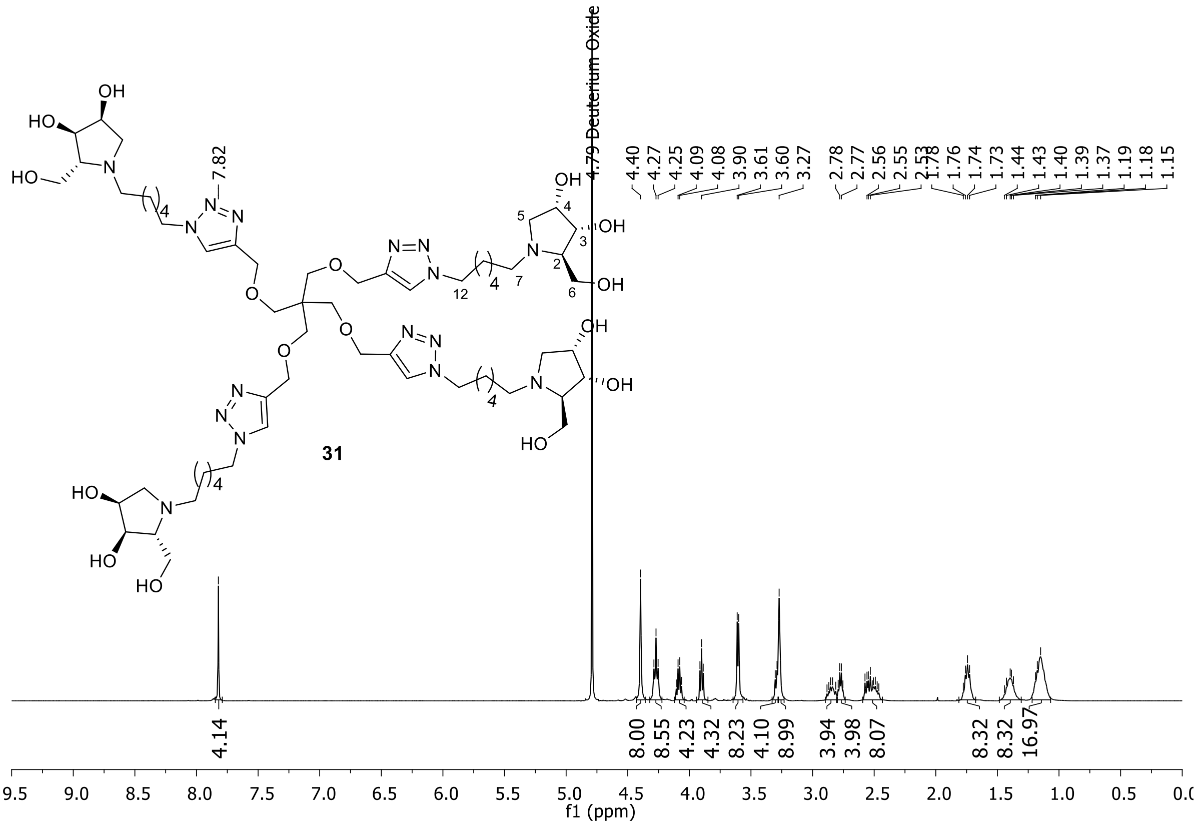

Figure S19: ^{1}H NMR spectrum of 31 (400 MHz, D_2O).

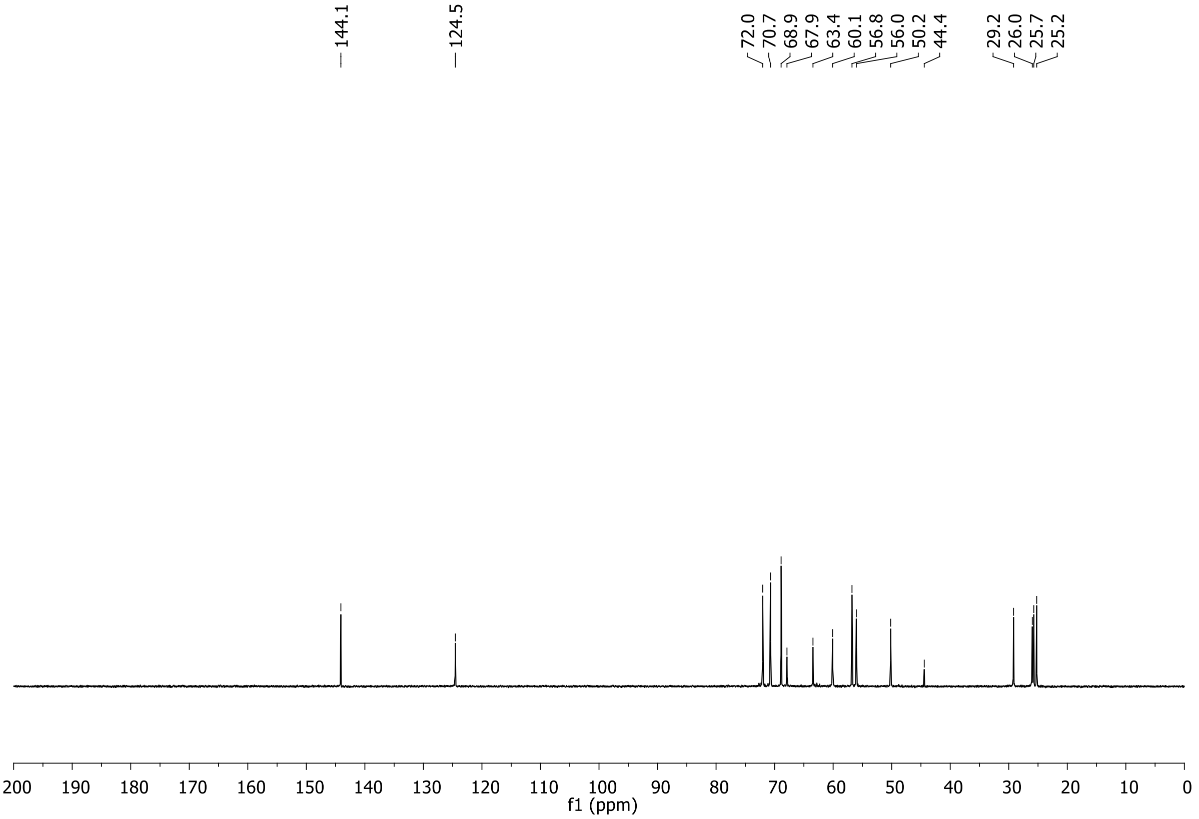

Figure S20: ^{13}C NMR spectrum of 31 (100 MHz, D_2O).

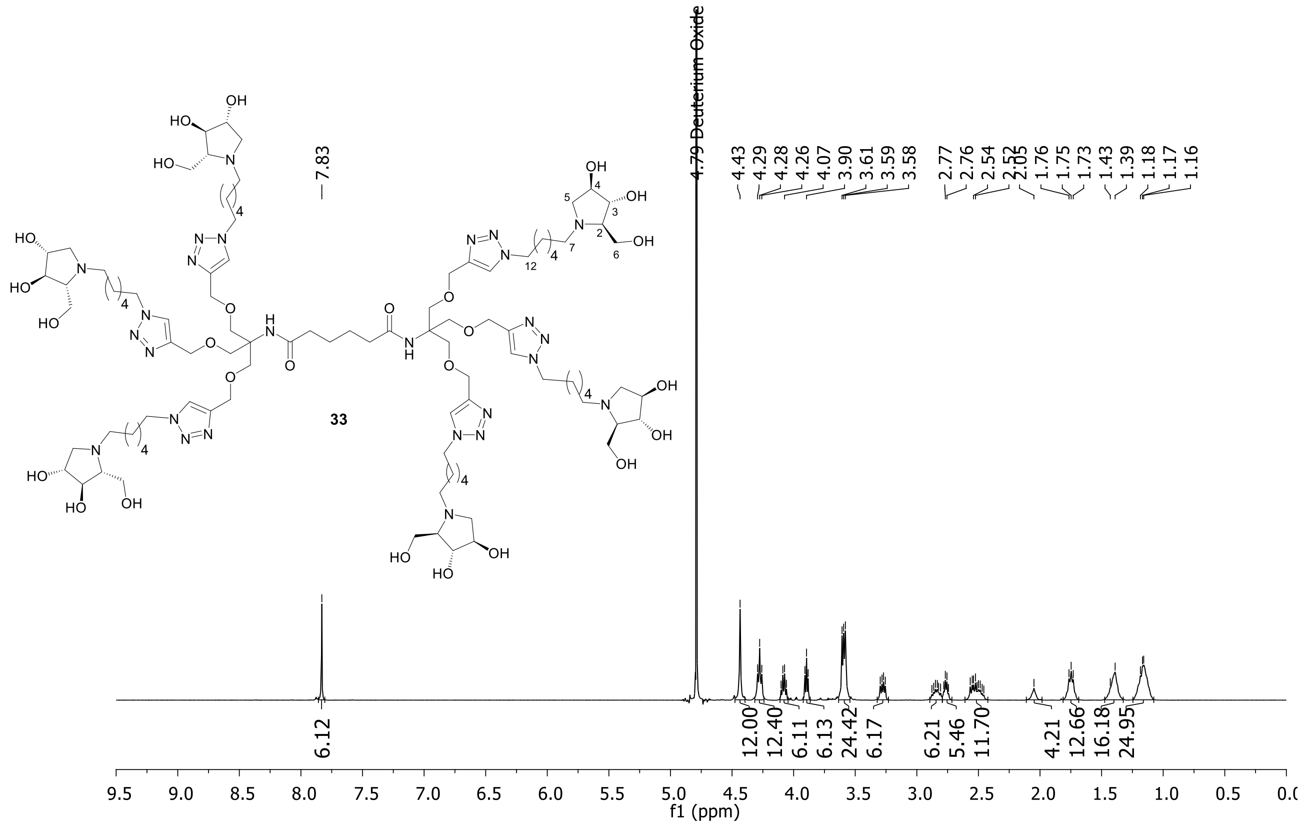

Figure S21: ^{1}H NMR spectrum of 33 (400 MHz, D_2O).

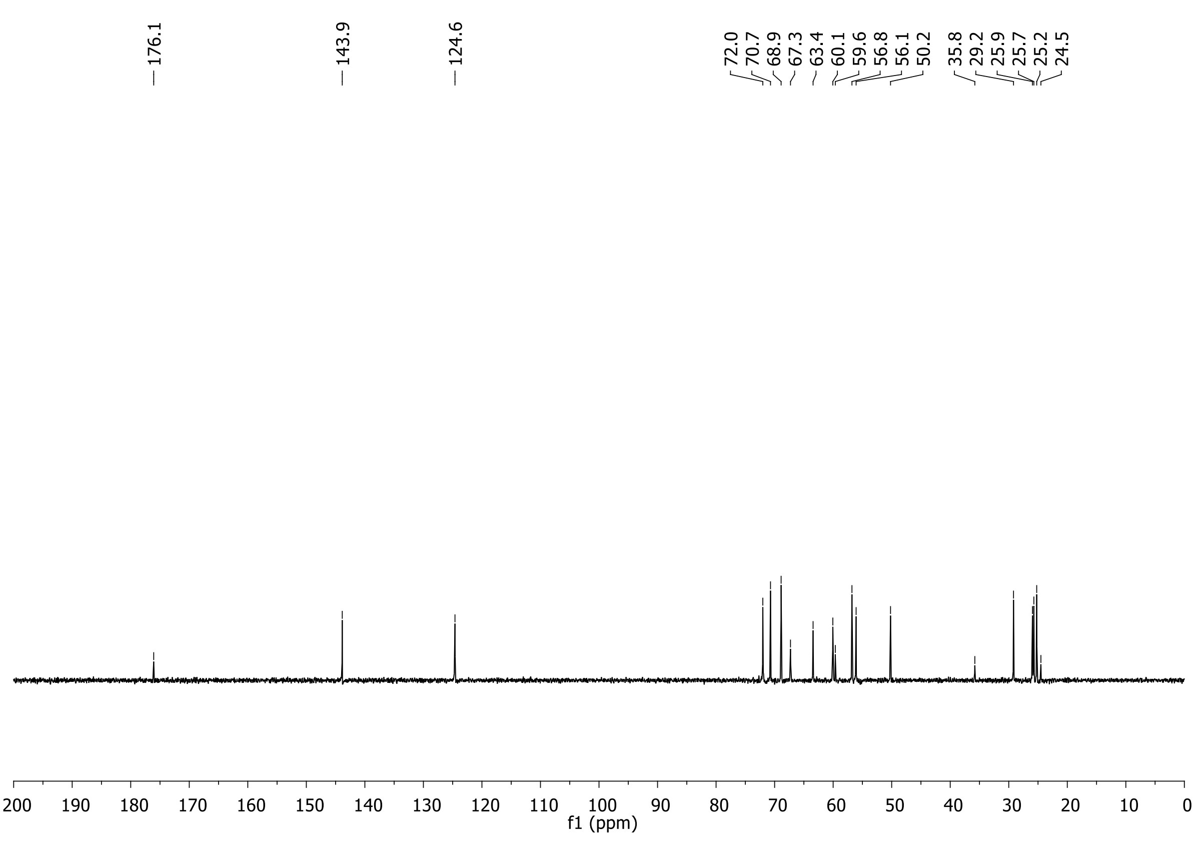

Figure S22: ^{13}C NMR spectrum of 33 (100 MHz, D_2O).

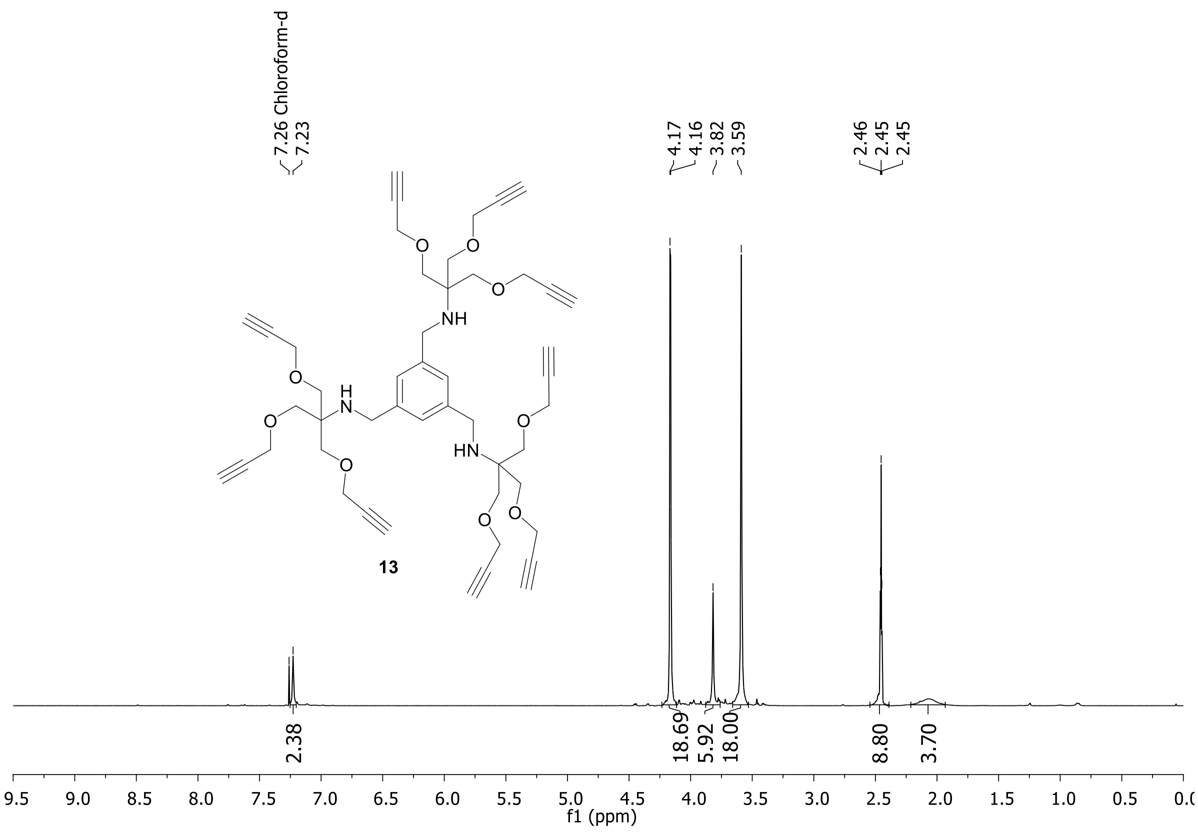

Figure S23: ^{1}H NMR spectrum of 13 (400 MHz, $CDCl_3$)

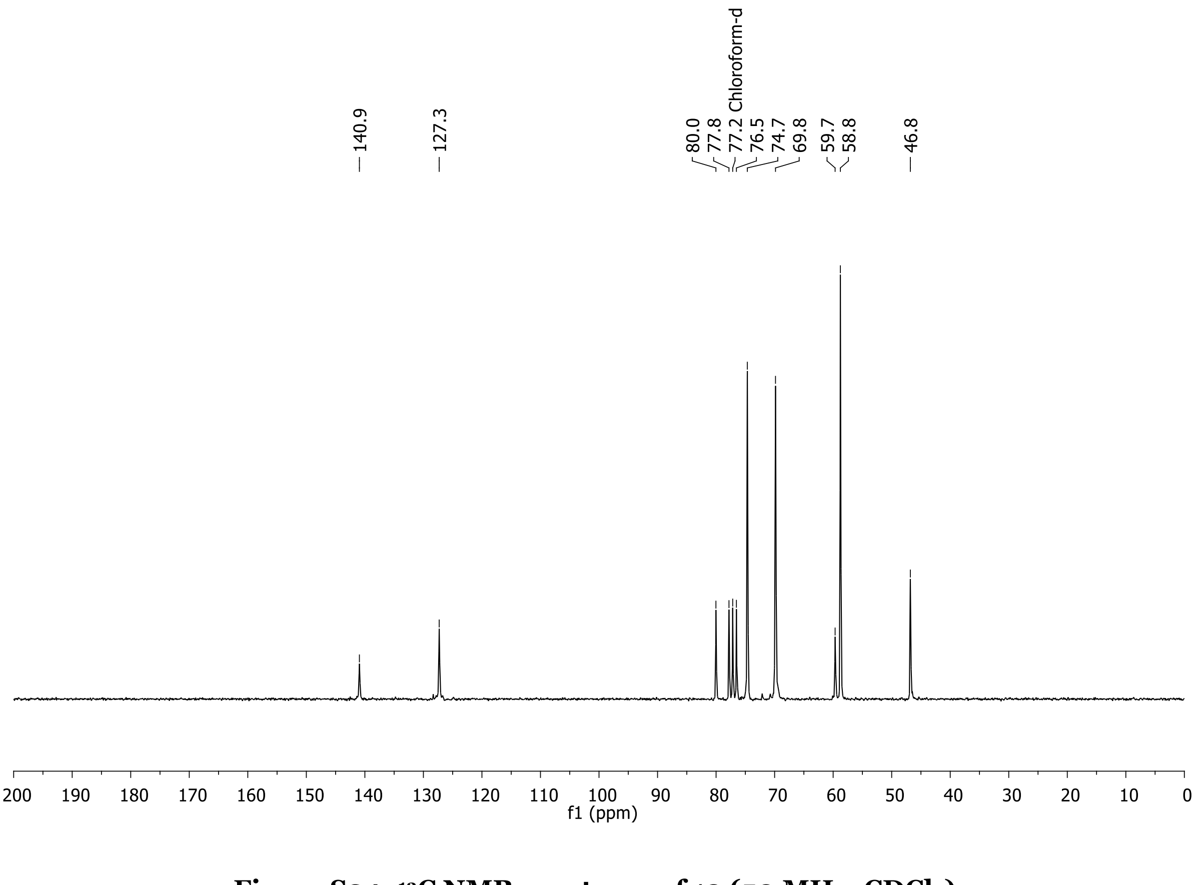

Figure S24: ^{13}C NMR spectrum of 13 (50 MHz, $CDCl_3$).

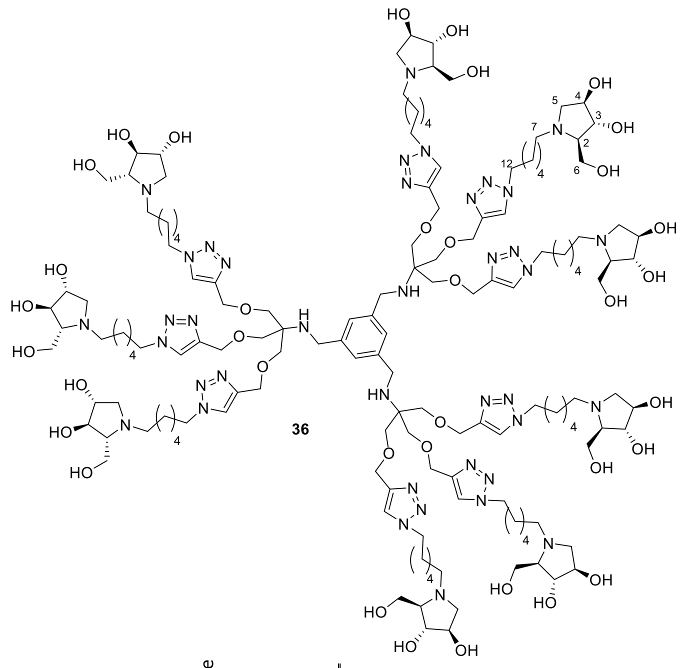

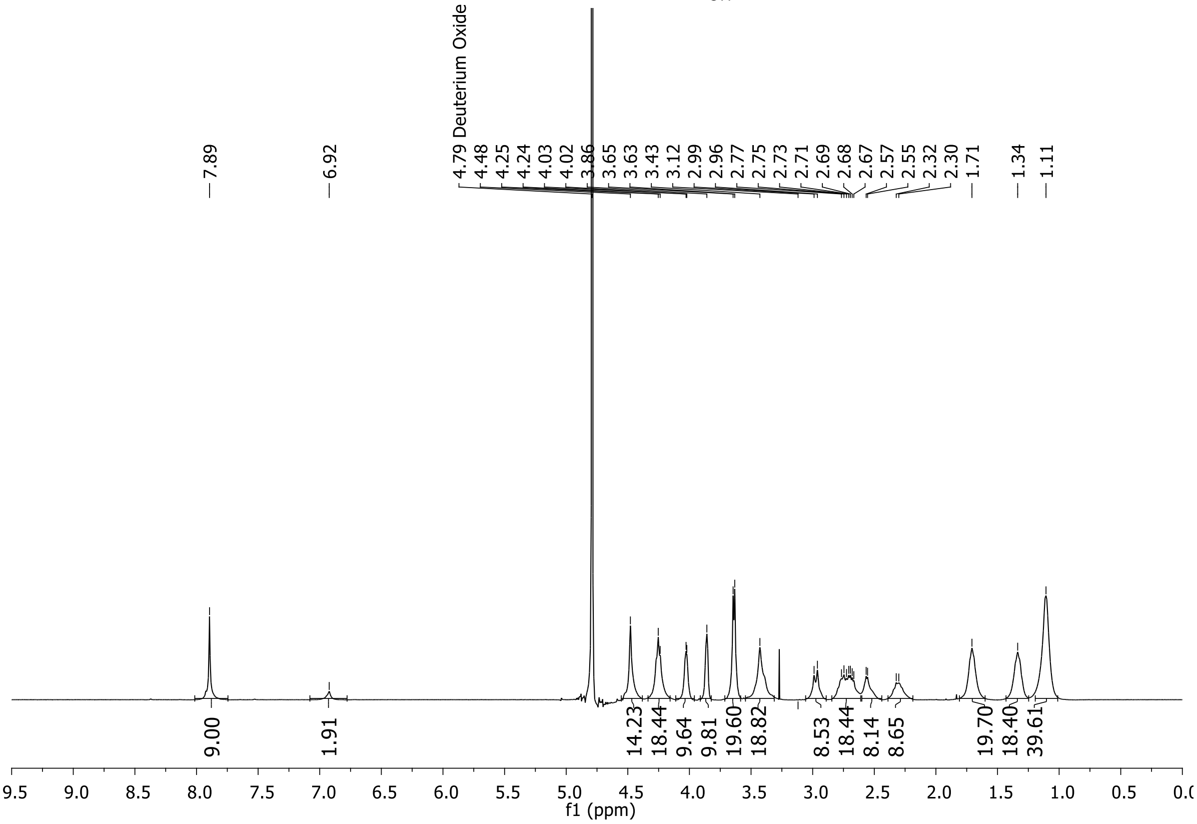

Figure S25: ^{1}H NMR spectrum of 36 (400 MHz, D_2O).

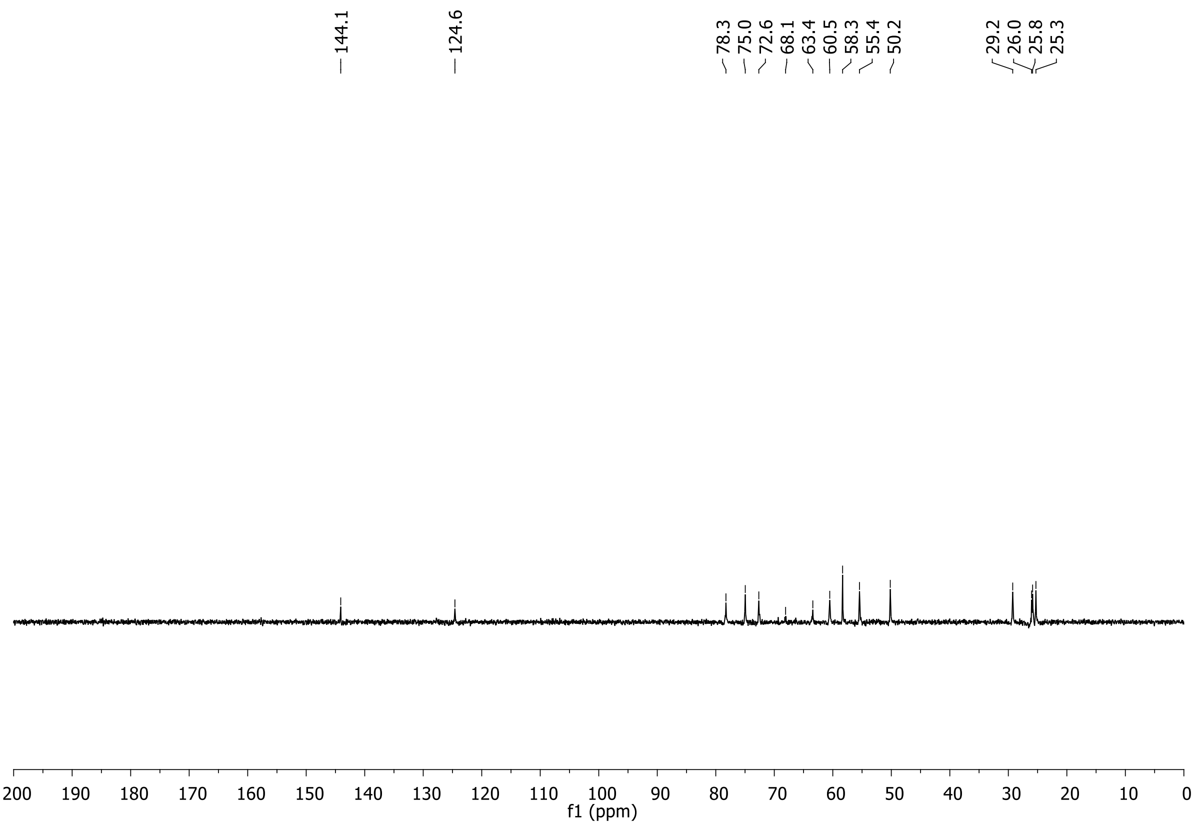

Figure S26: ^{13}C NMR spectrum of 36 (100 MHz, D_2O).

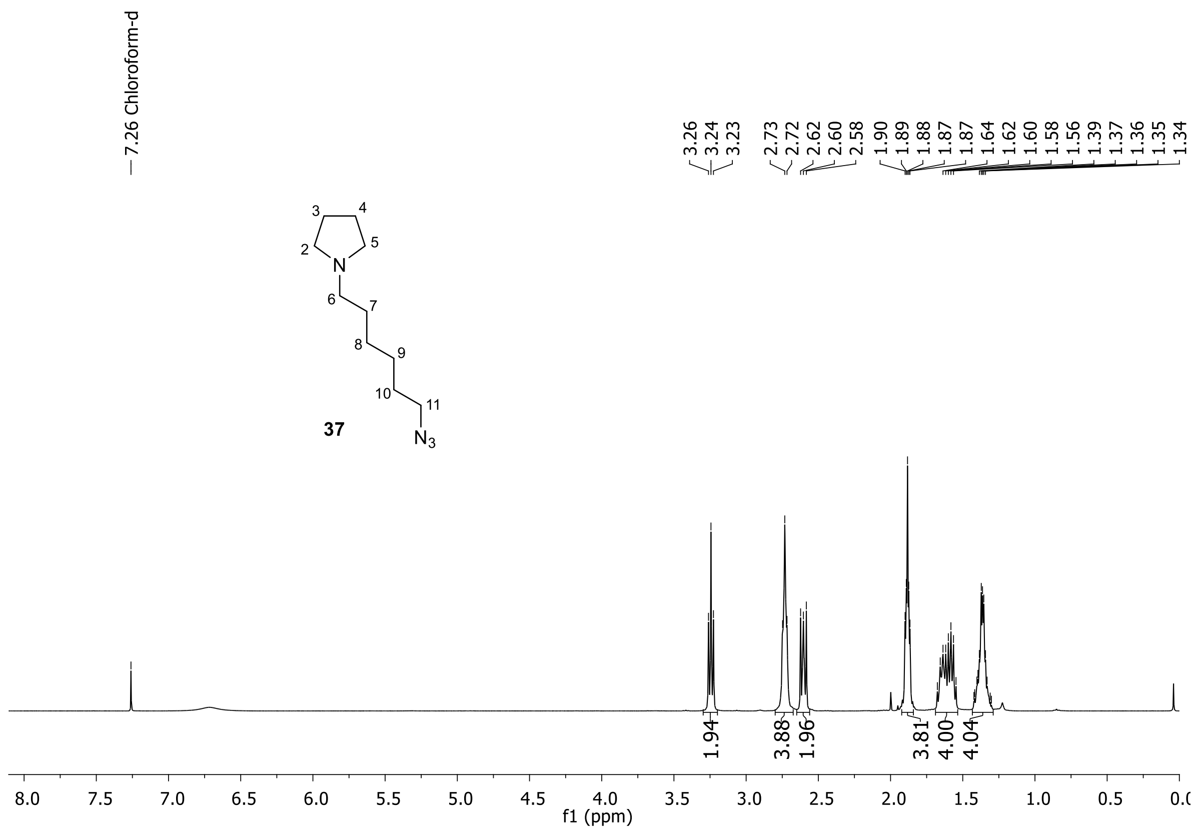

Figure S27: ^{1}H NMR spectrum of 37 (400 MHz, $CDCl_3$)

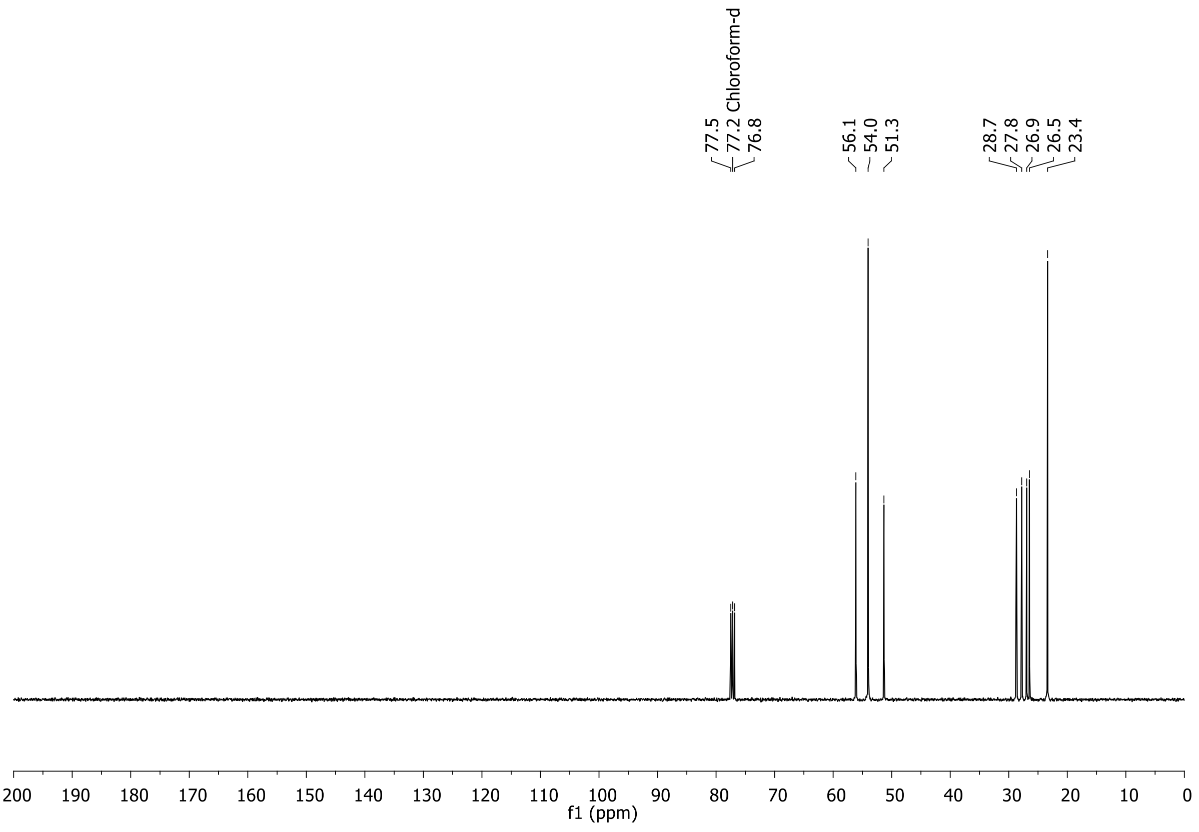

Figure S28: ^{13}C NMR spectrum of 37 (100 MHz, $CDCl_3$).

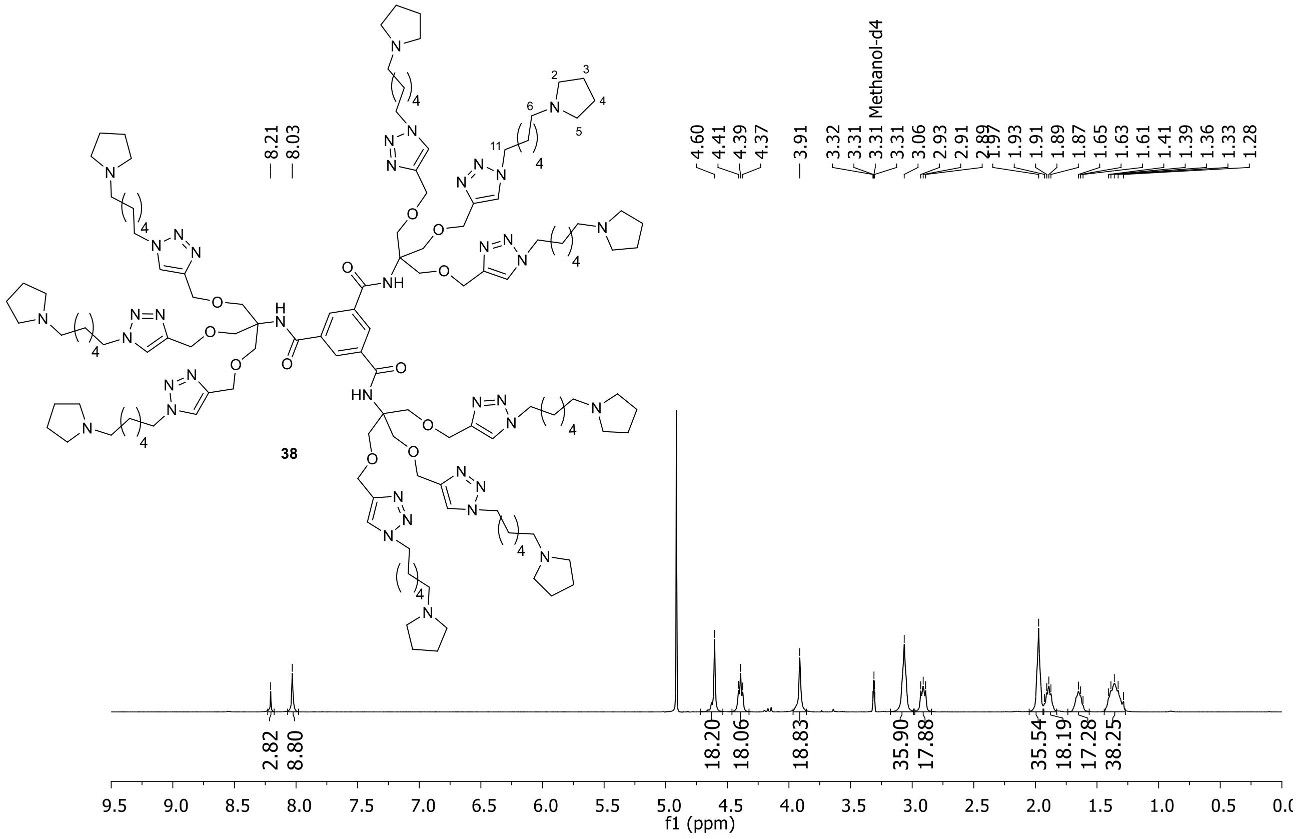

Figure S29: ^{1}H NMR spectrum of 38 (400 MHz, CD_3OD)

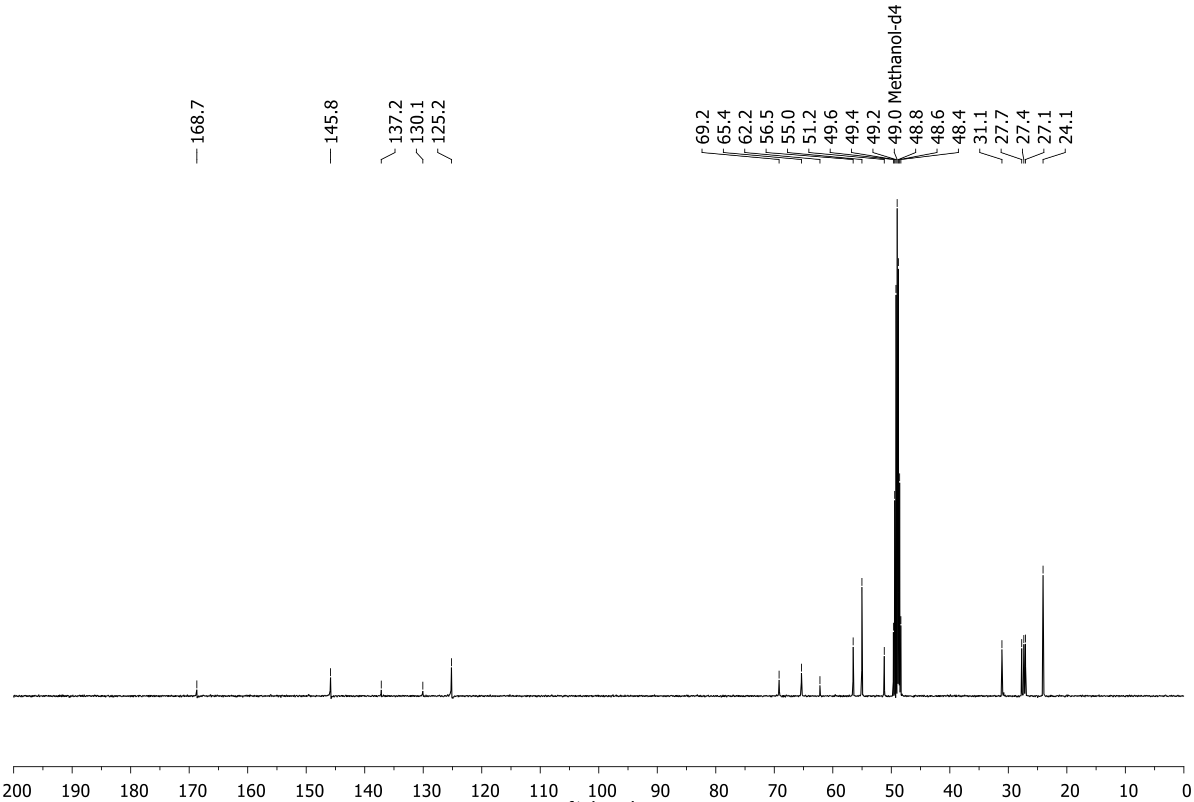

Figure S30: ^{13}C NMR spectrum of 38(100 MHz, CD_3OD).

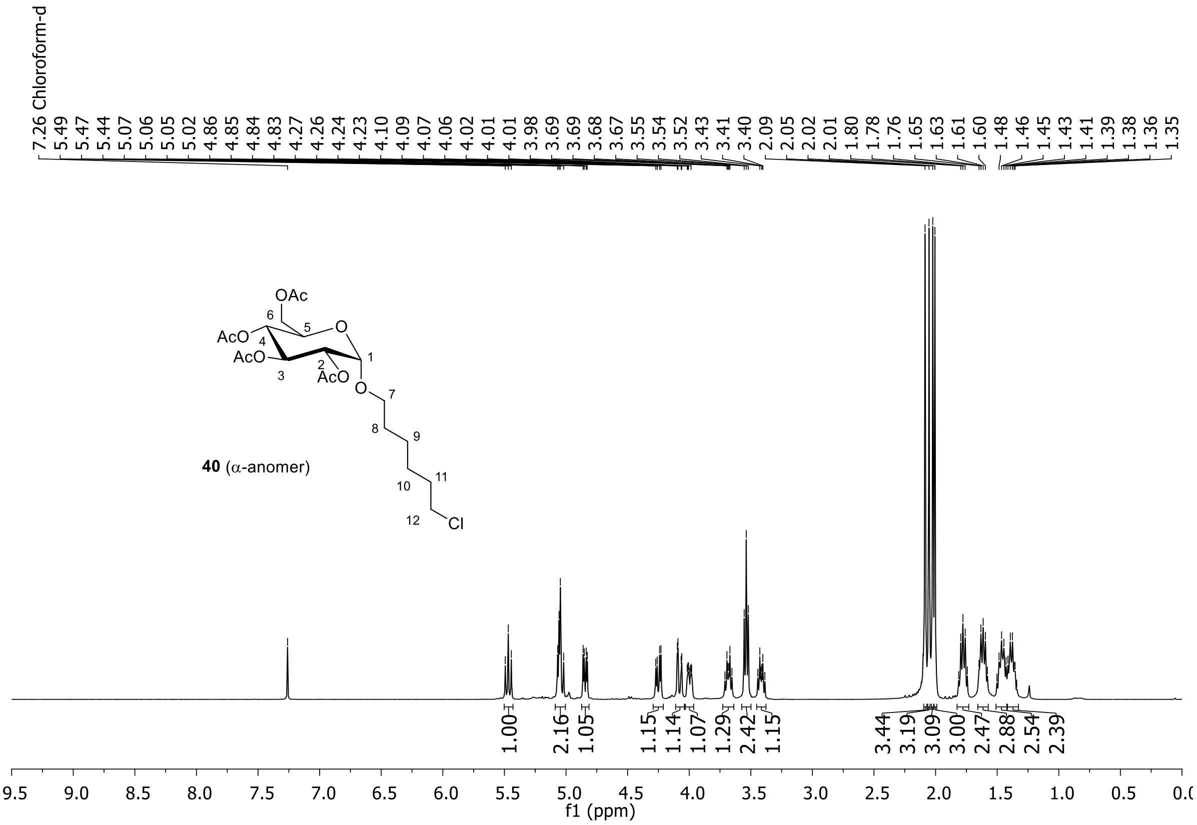

Figure S31: ^1H NMR spectrum of 40 (400 MHz, $CDCl_3$).

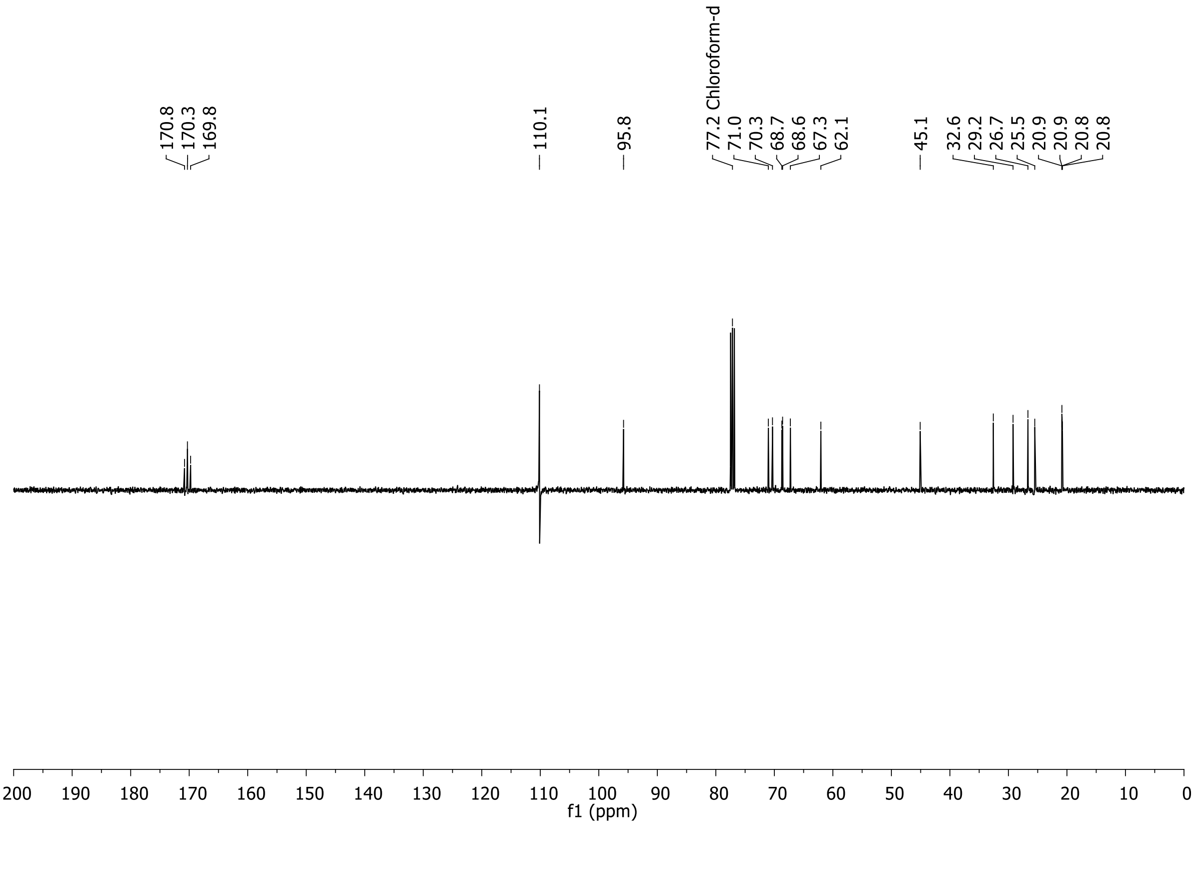

Figure S32: ^{13}C NMR spectrum of 40 (100 MHz, $CDCl_3$).

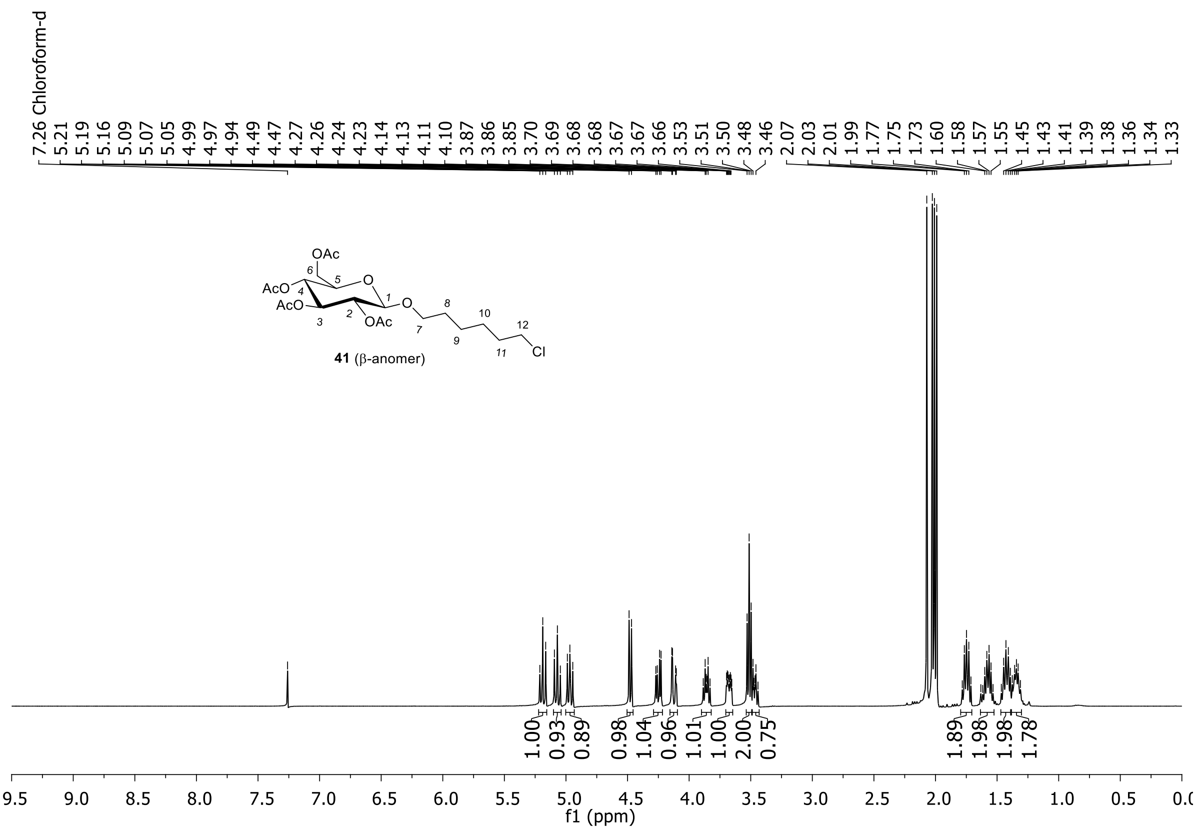

Figure S33: ^{1}H NMR spectrum of 41 (400 MHz, $CDCl_3$).

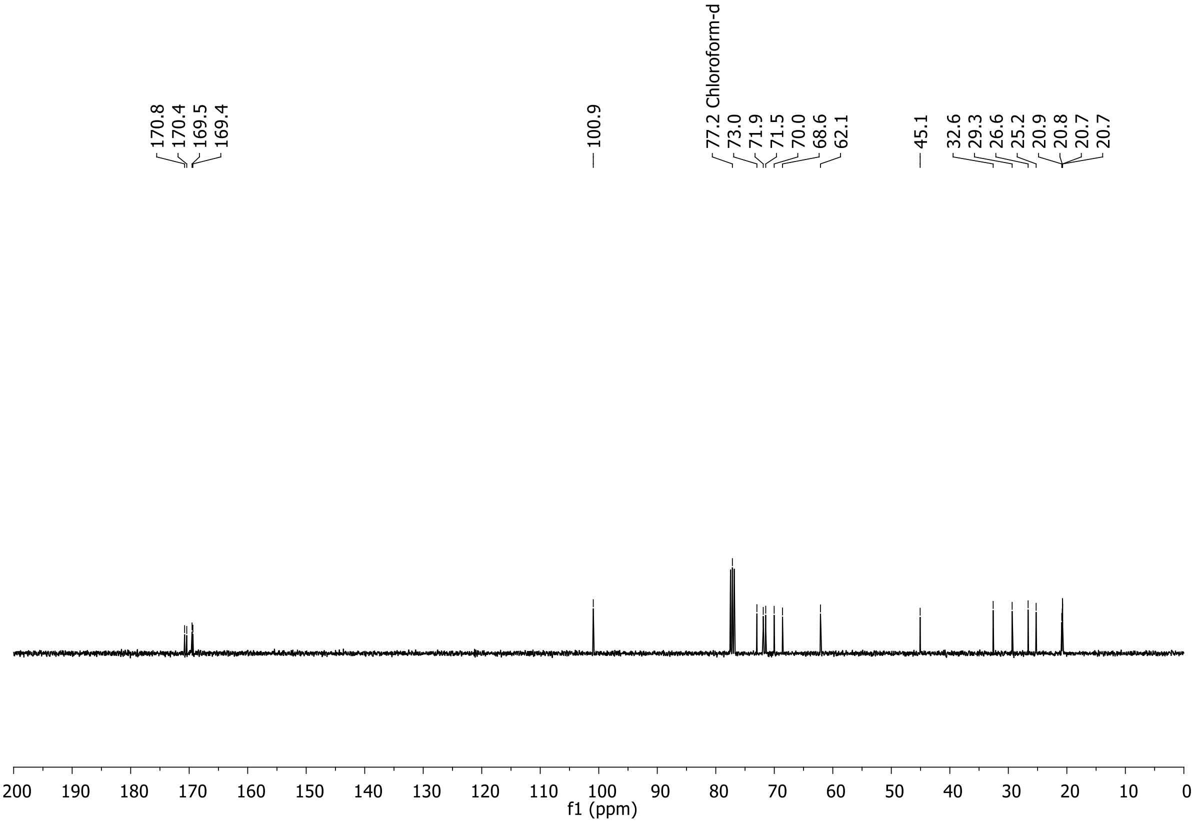

Figure S34: ^{13}C NMR spectrum of 41 (100 MHz, $CDCl_3$).

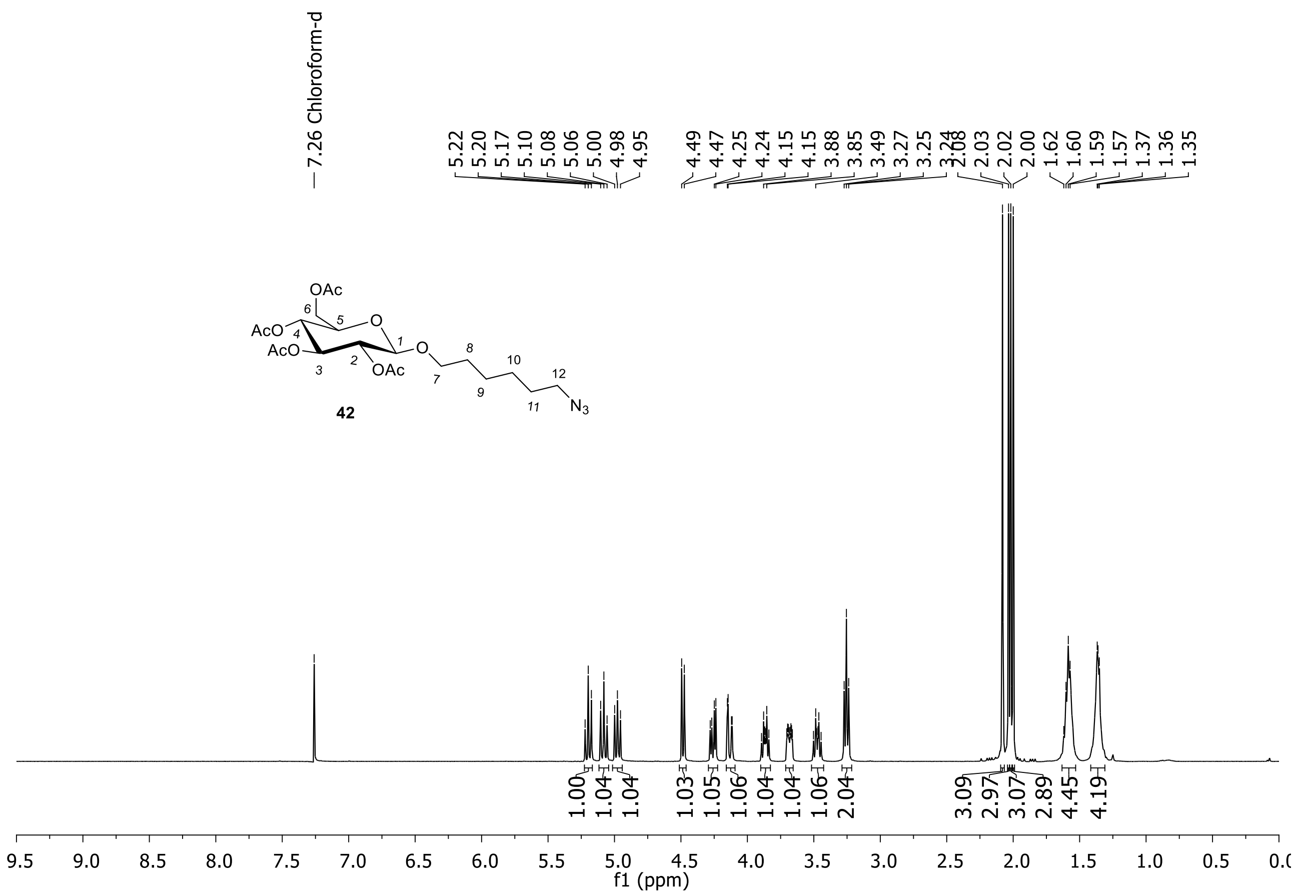

Figure S35: ^{1}H NMR spectrum of 42 (400 MHz, $CDCl_3$).

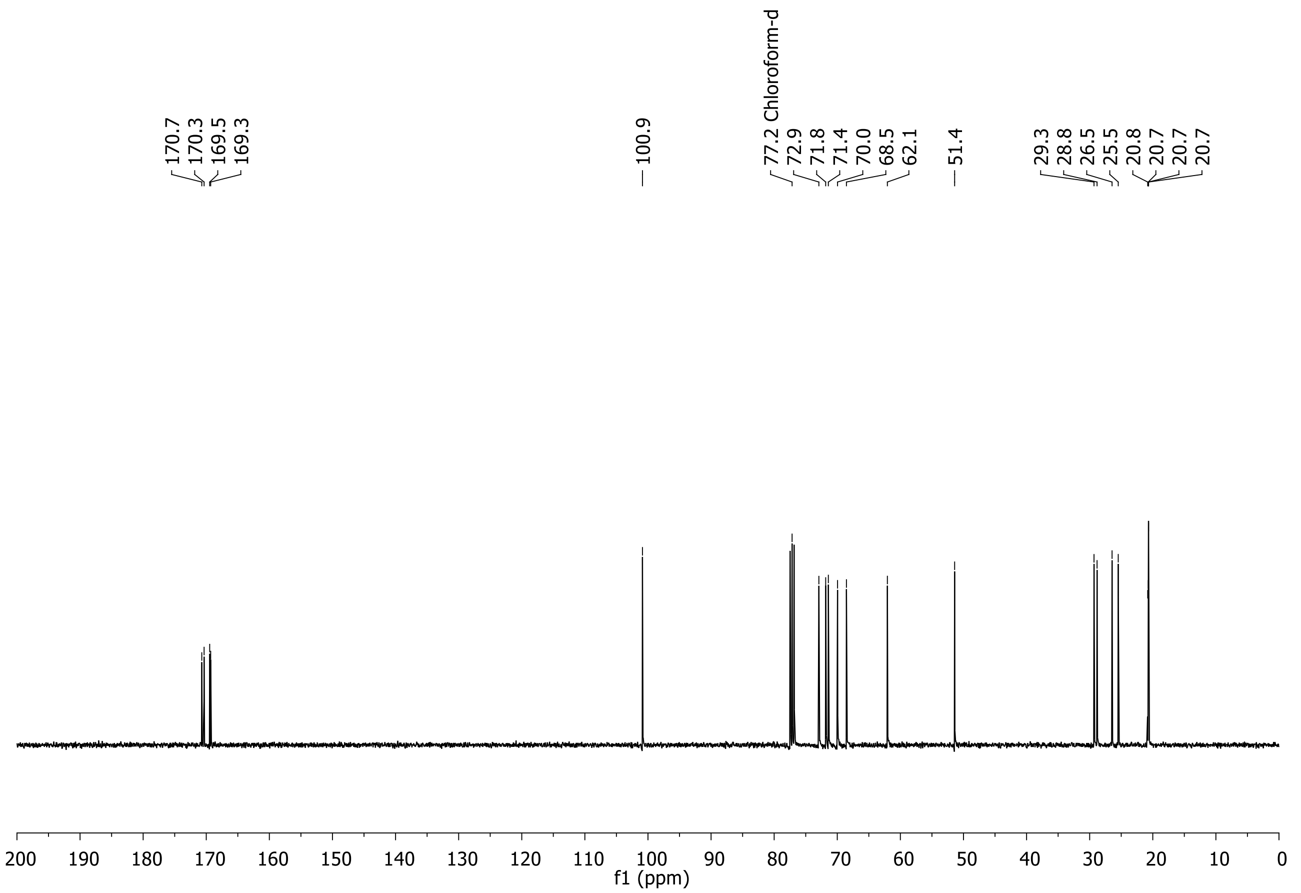

Figure S36: ^{13}C NMR spectrum of 42 (100 MHz, $CDCl_3$).

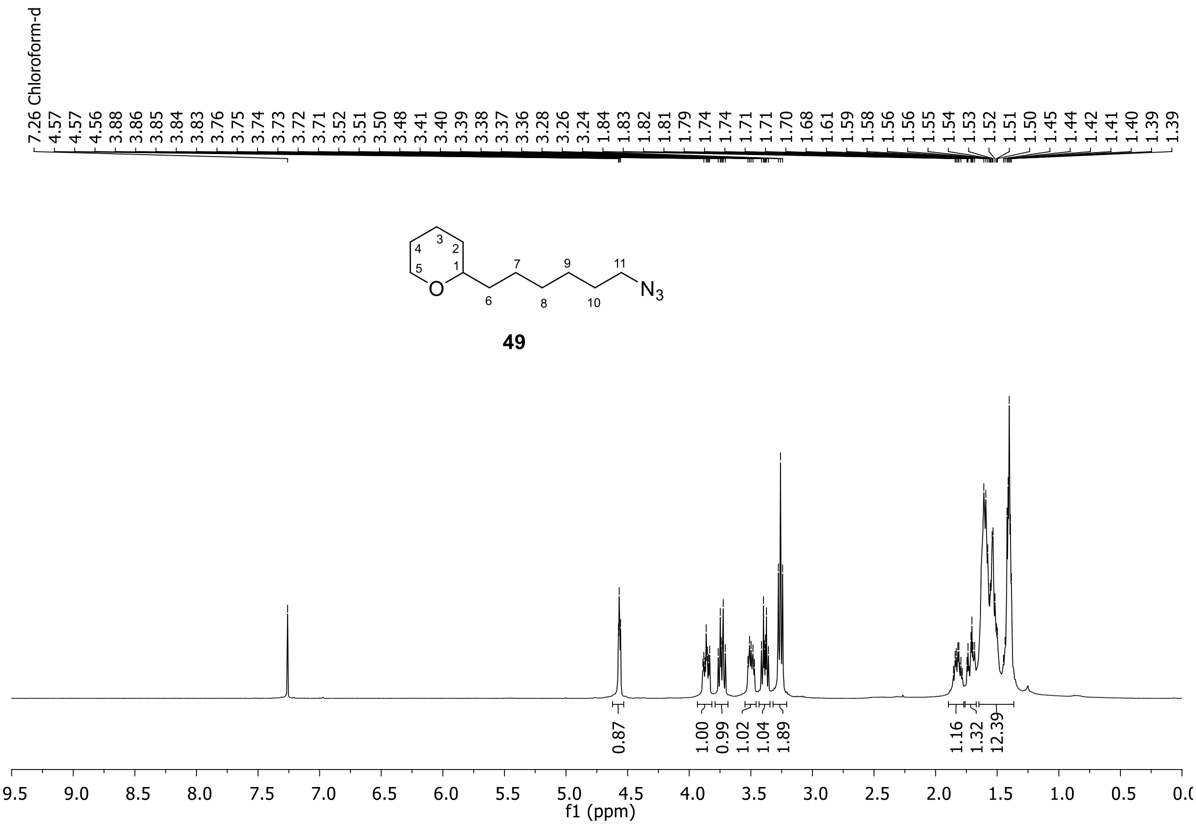

Figure S37: ^{1}H NMR spectrum of 49 (400 MHz, $CDCl_3$).

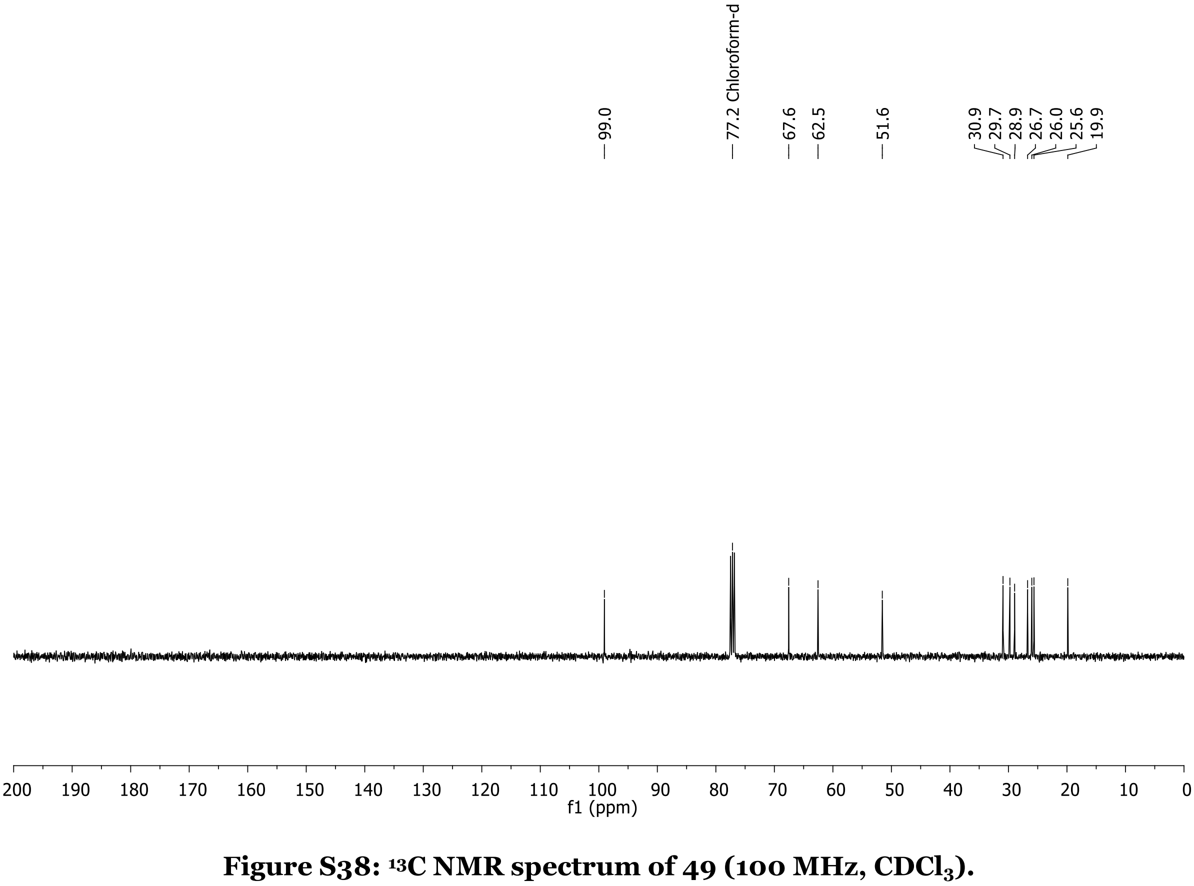

Figure S38: ^{13}C NMR spectrum of 49 (100 MHz, $CDCl_3$).

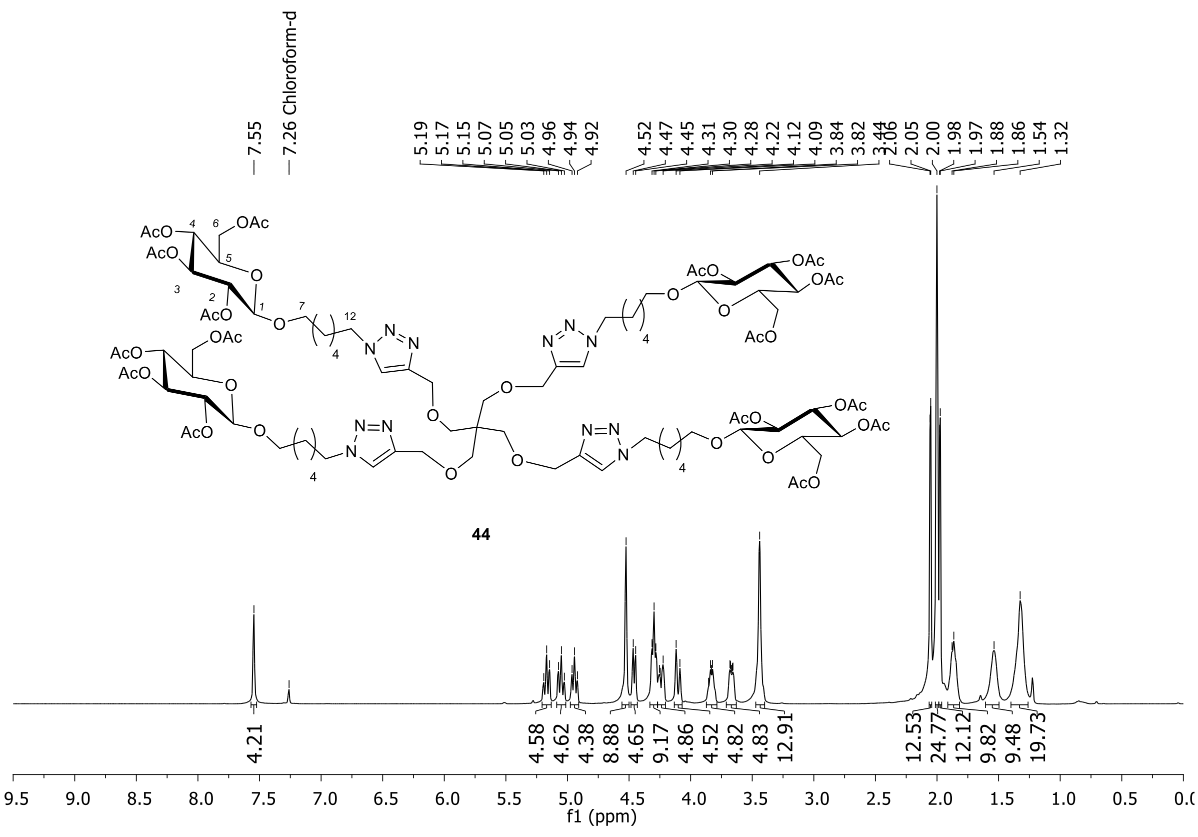

Figure S39: ^{1}H NMR spectrum of 44 (400 MHz, $CDCl_3$).

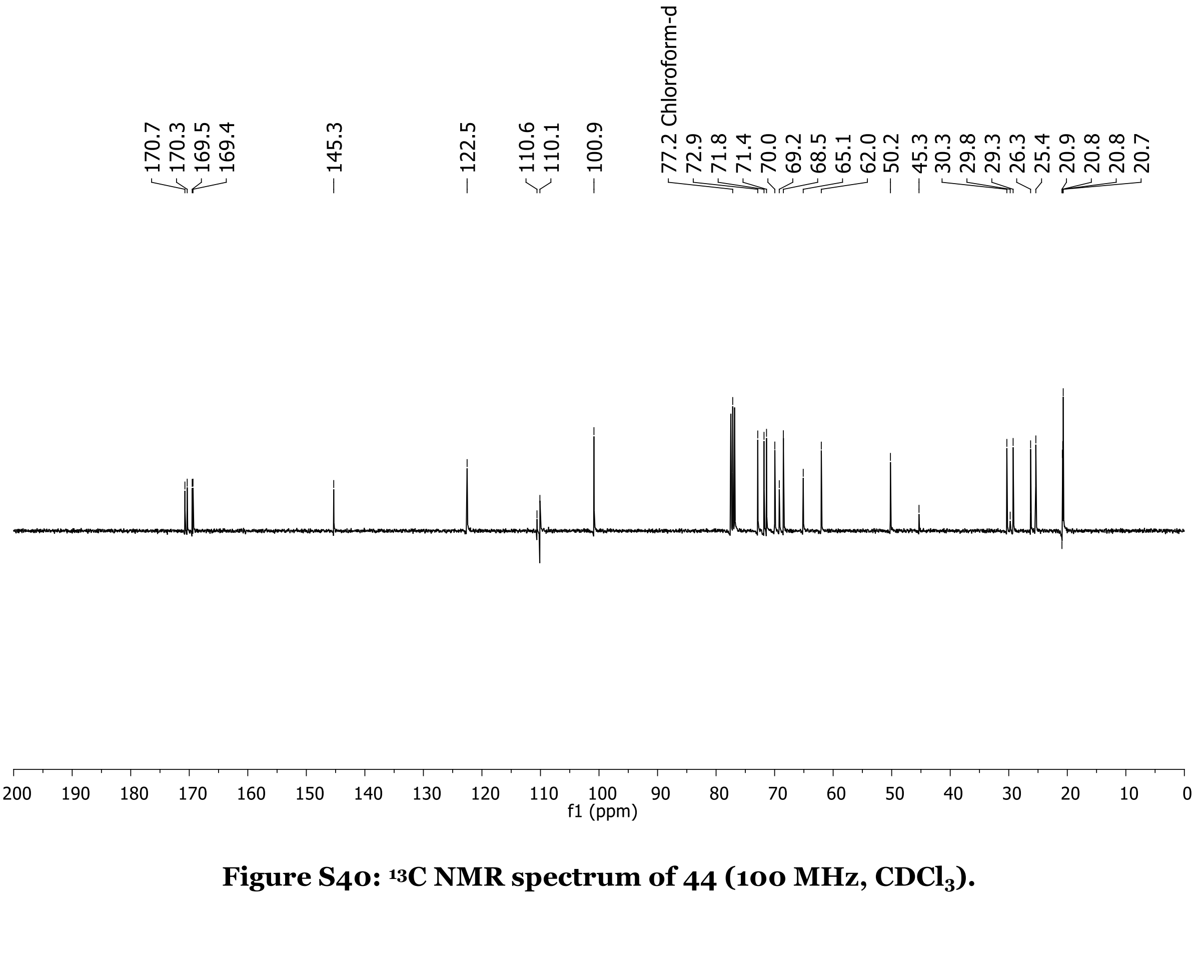

Figure S40: ^{13}C NMR spectrum of 44 (100 MHz, $CDCl_3$).

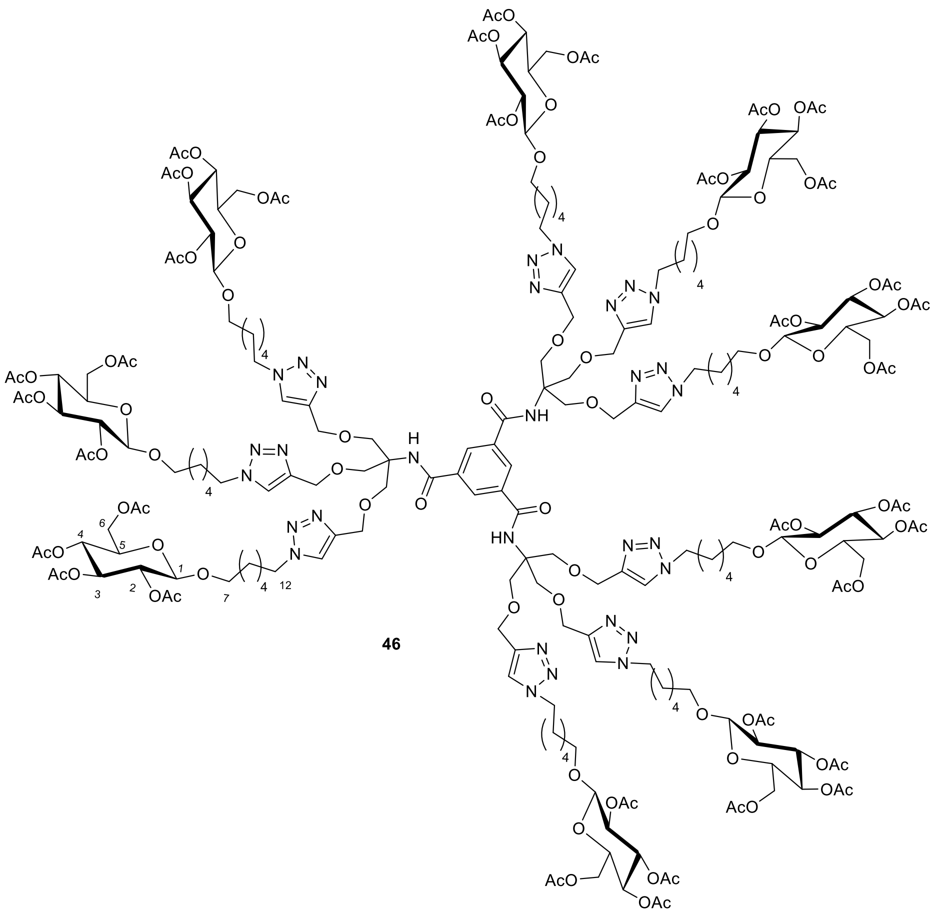

46

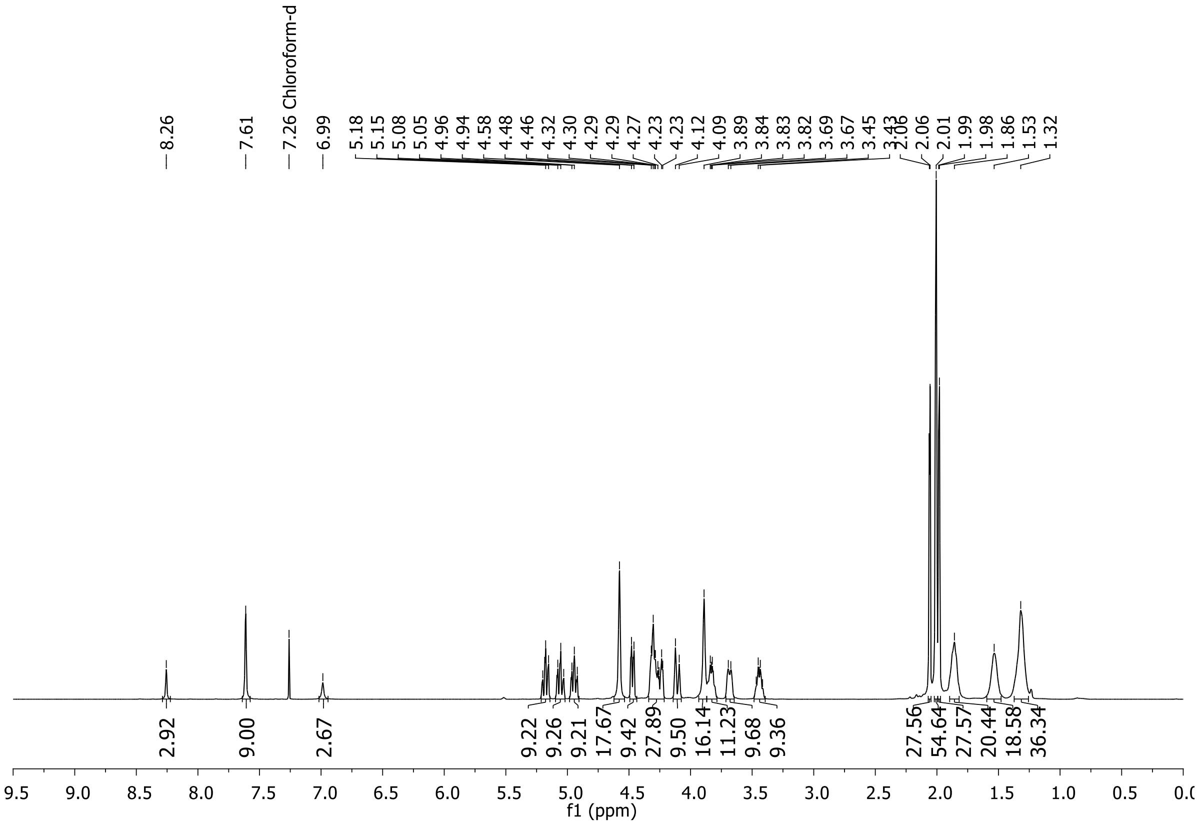

Figure S41: ¹H NMR spectrum of 46 (400 MHz, $CDCl_3$).

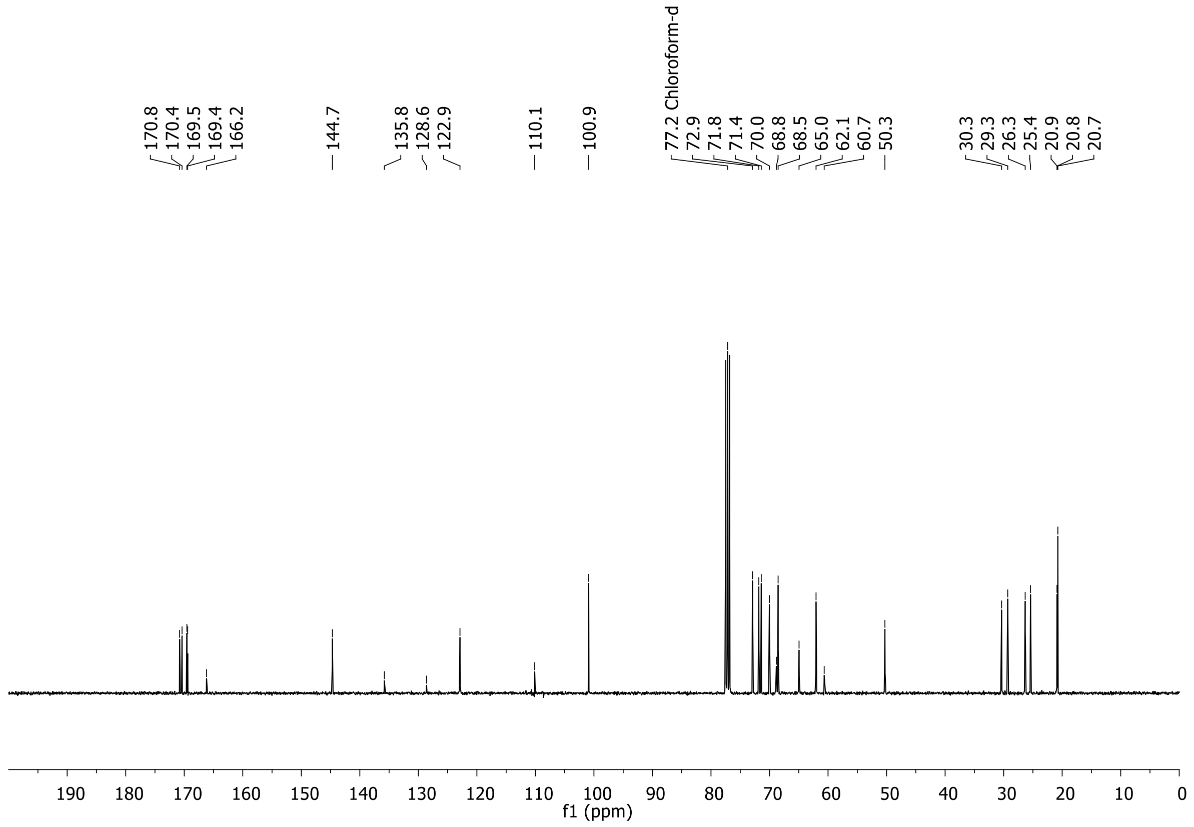

Figure S42: ^{13}C NMR spectrum of 46 (100 MHz, $CDCl_3$).

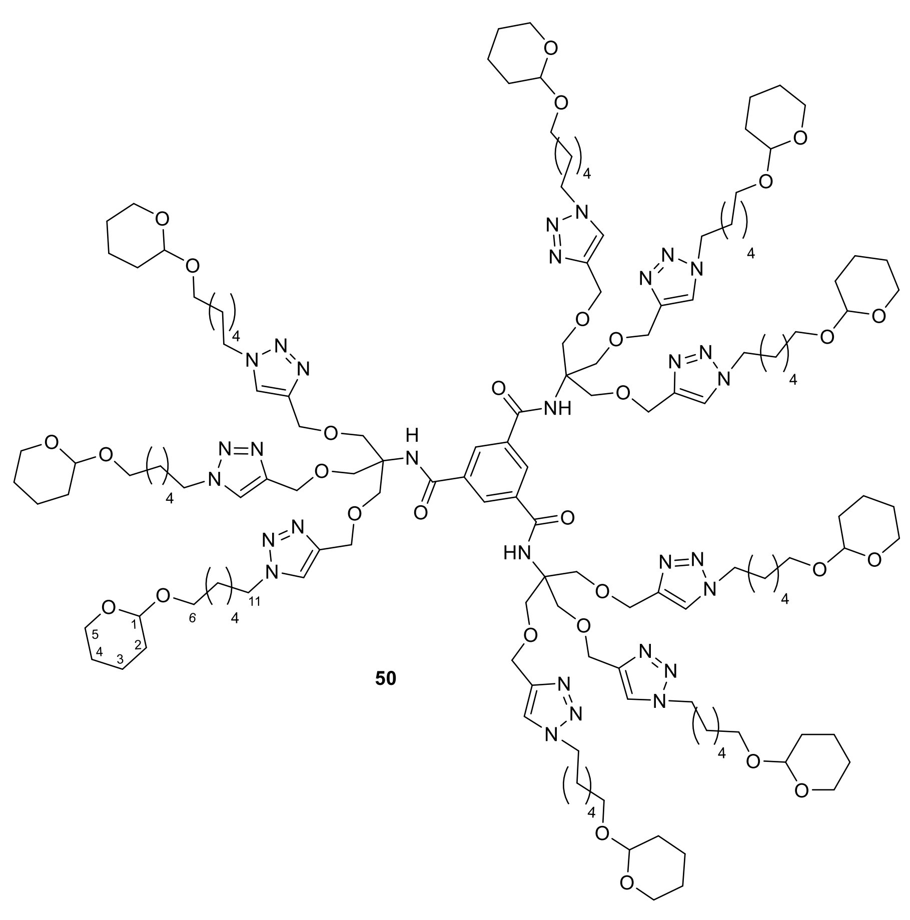

50

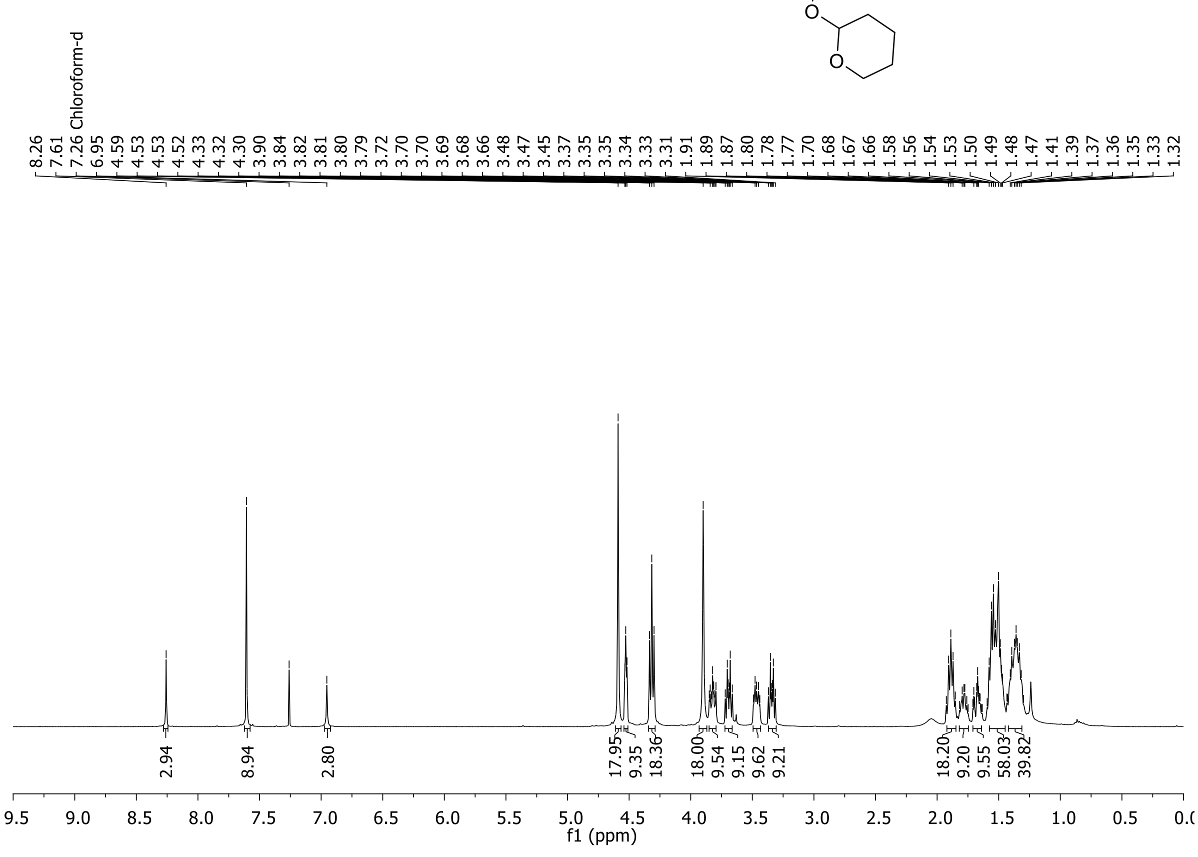

Figure S43: ^{1}H NMR spectrum of 50 (400 MHz, $CDCl_3$).

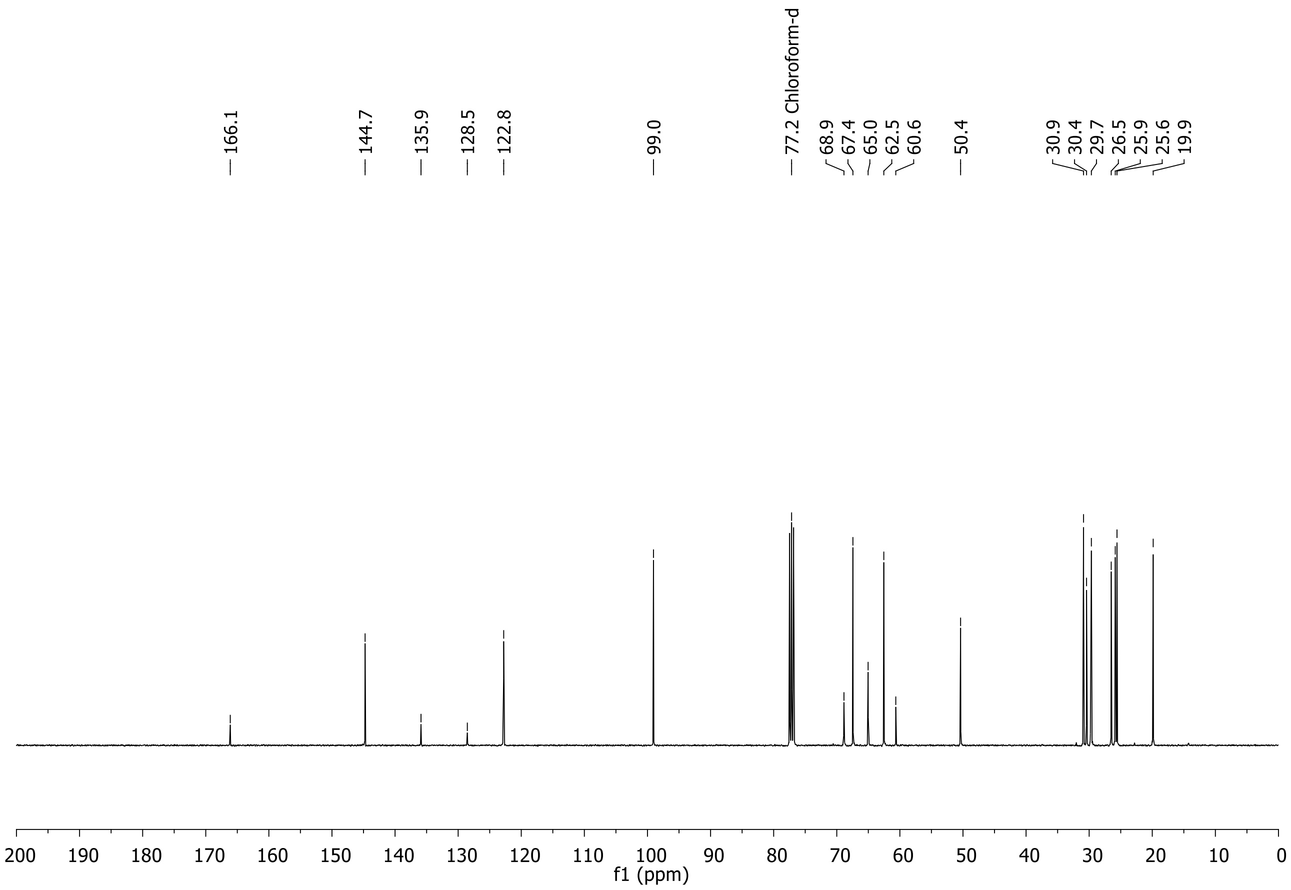

Figure S44: ^{13}C NMR spectrum of 50 (100 MHz, $CDCl_3$).

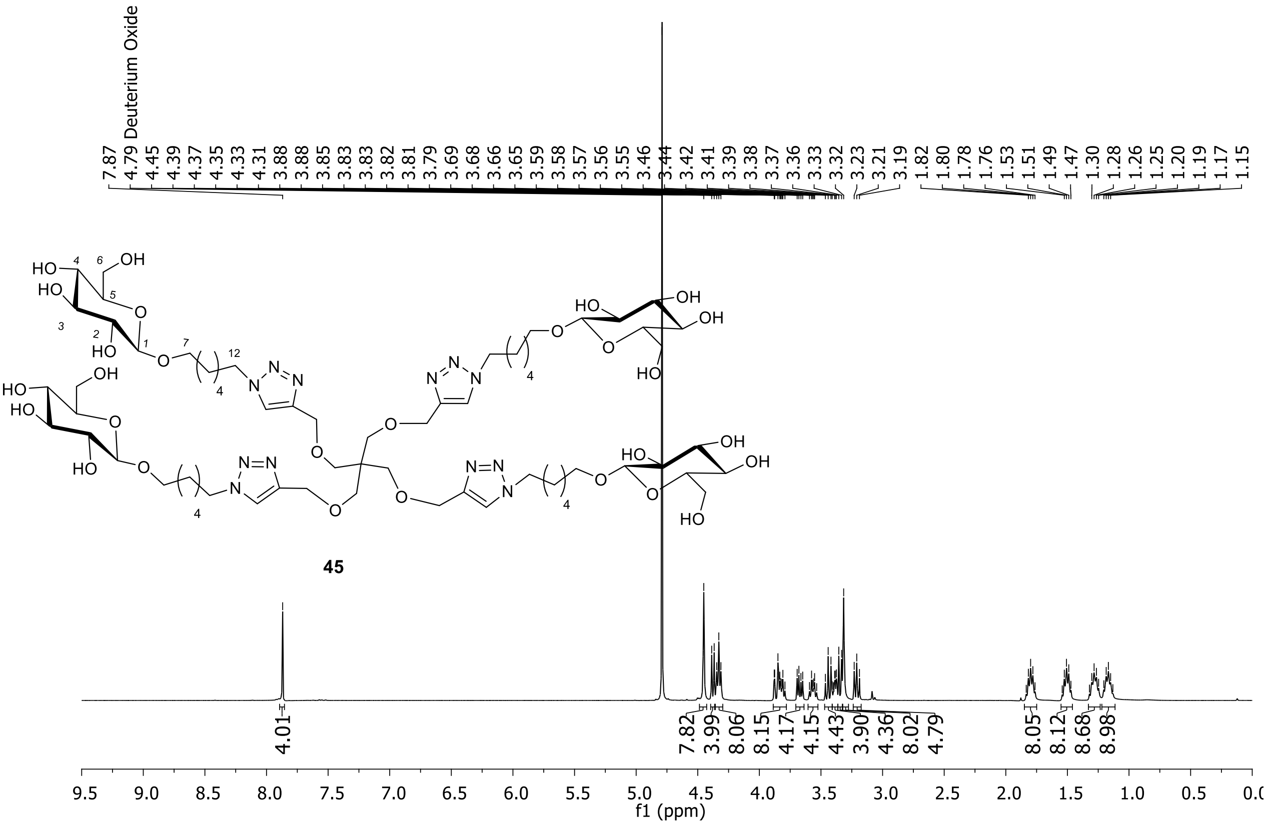

Figure S45: ^{1}H NMR spectrum of 45 (400 MHz, D_2O).

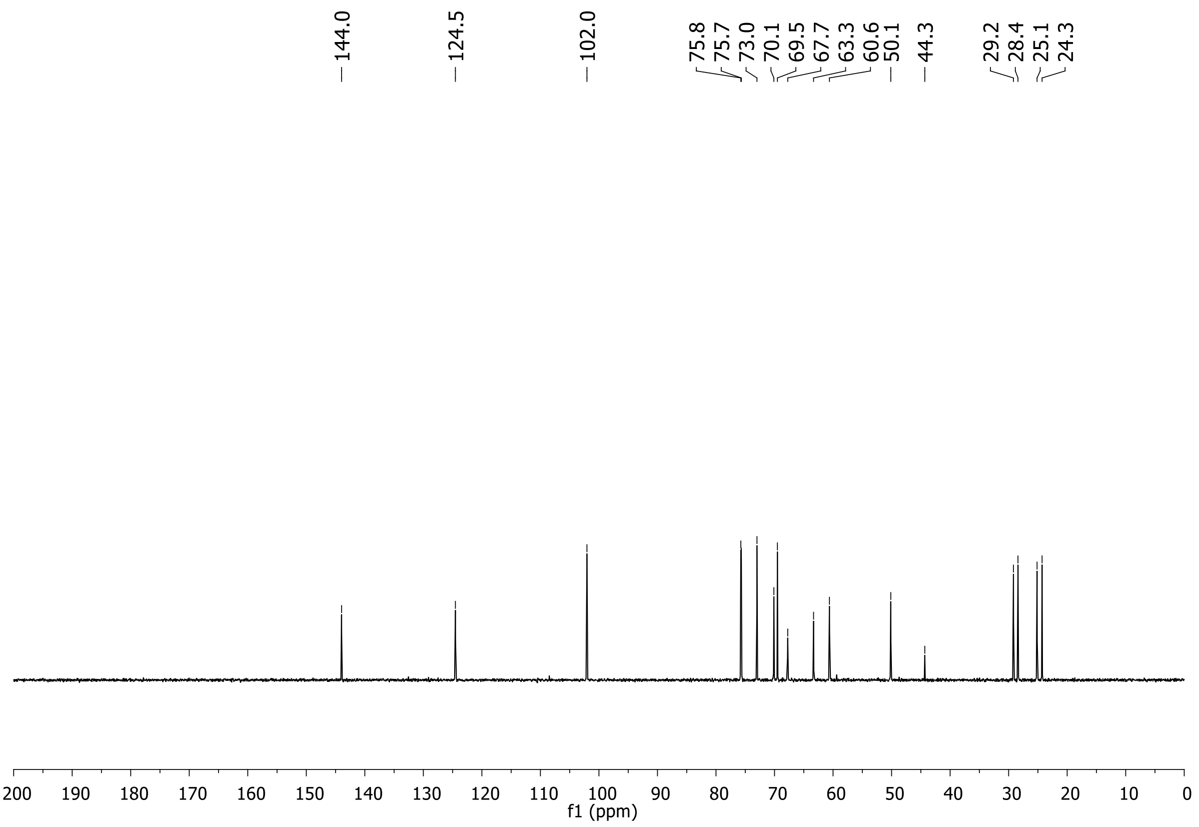

Figure S46: ^{13}C NMR spectrum of 45 (100 MHz, D_2O).

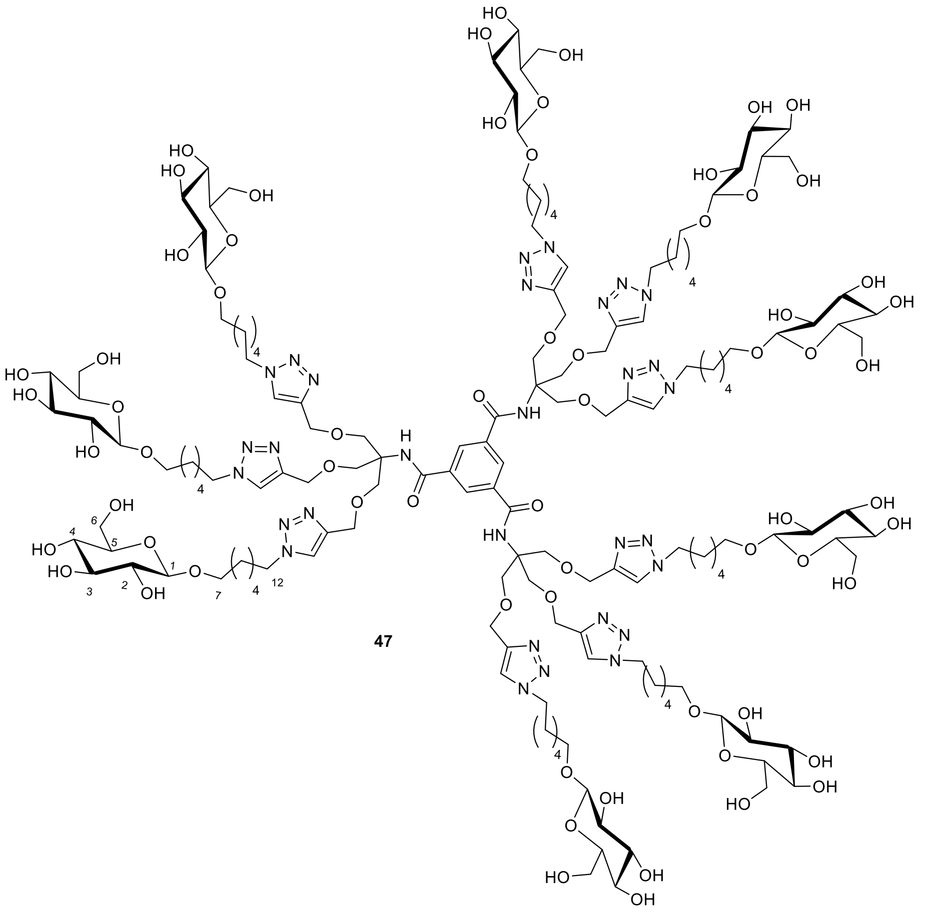

47

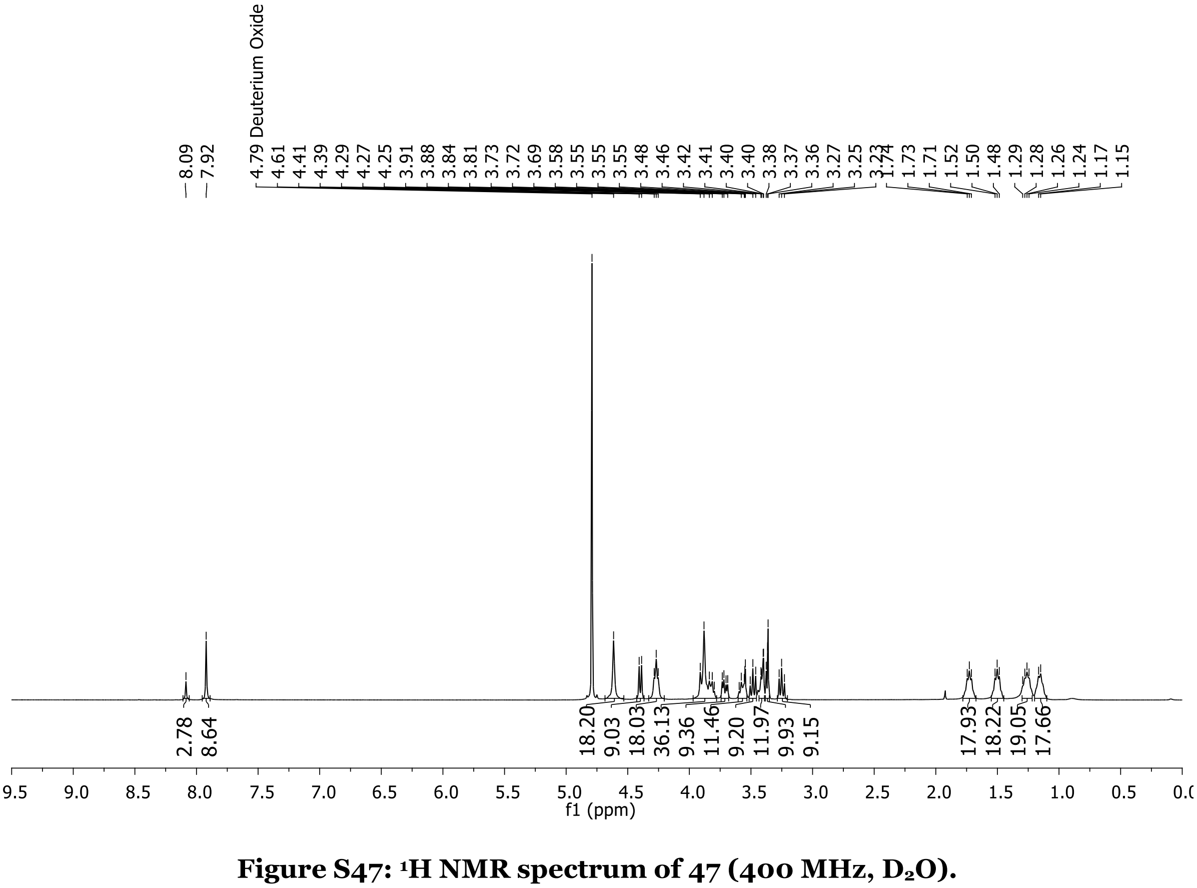

Figure S47: ^{1}H NMR spectrum of 47 (400 MHz, D_2O).

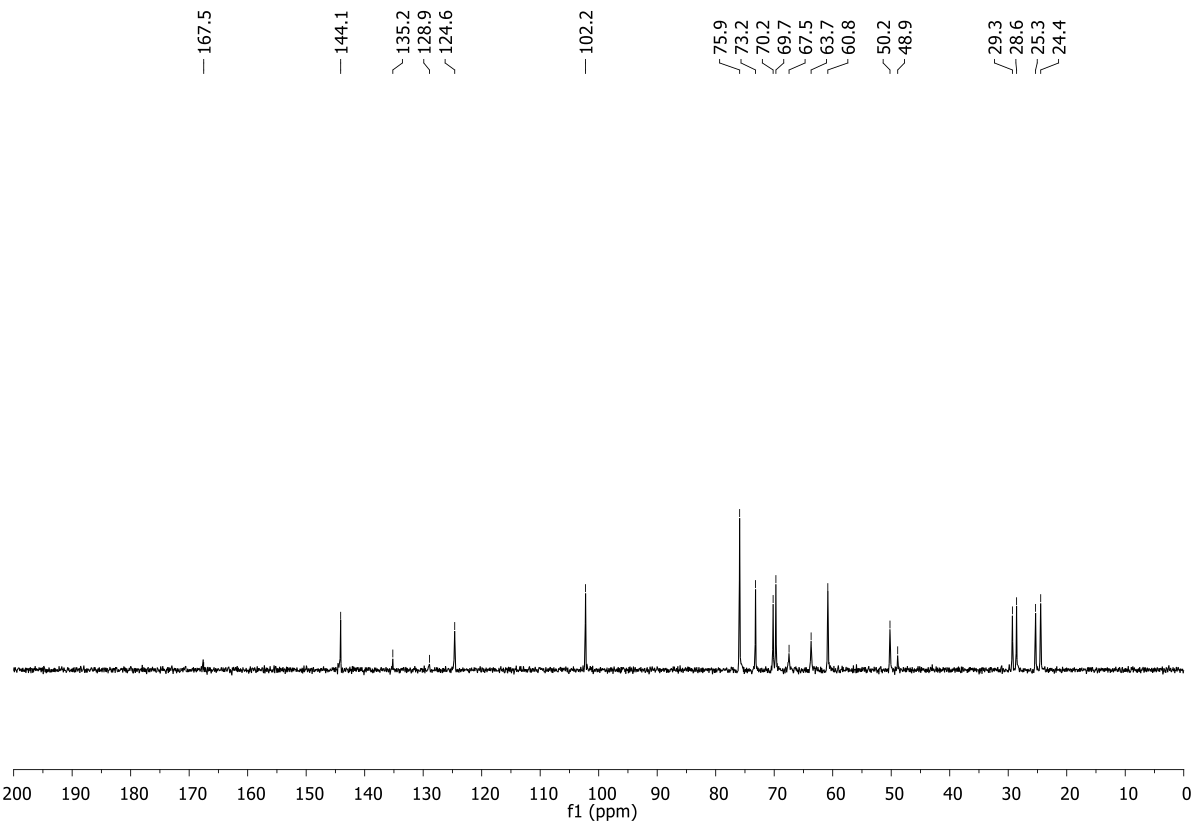

Figure S48: ^{13}C NMR spectrum of 47 (50 MHz, D_2O).

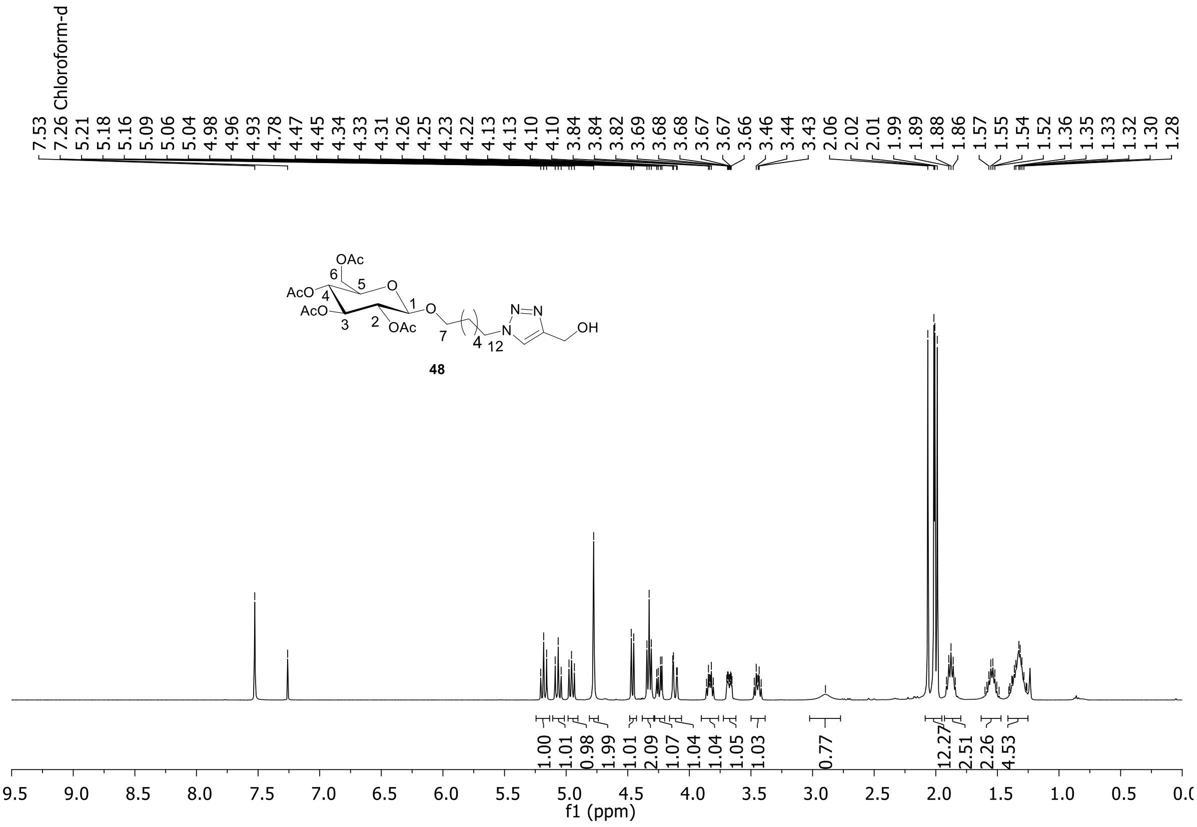

Figure S49: ^{1}H NMR spectrum of 48 (400 MHz, $CDCl_3$).

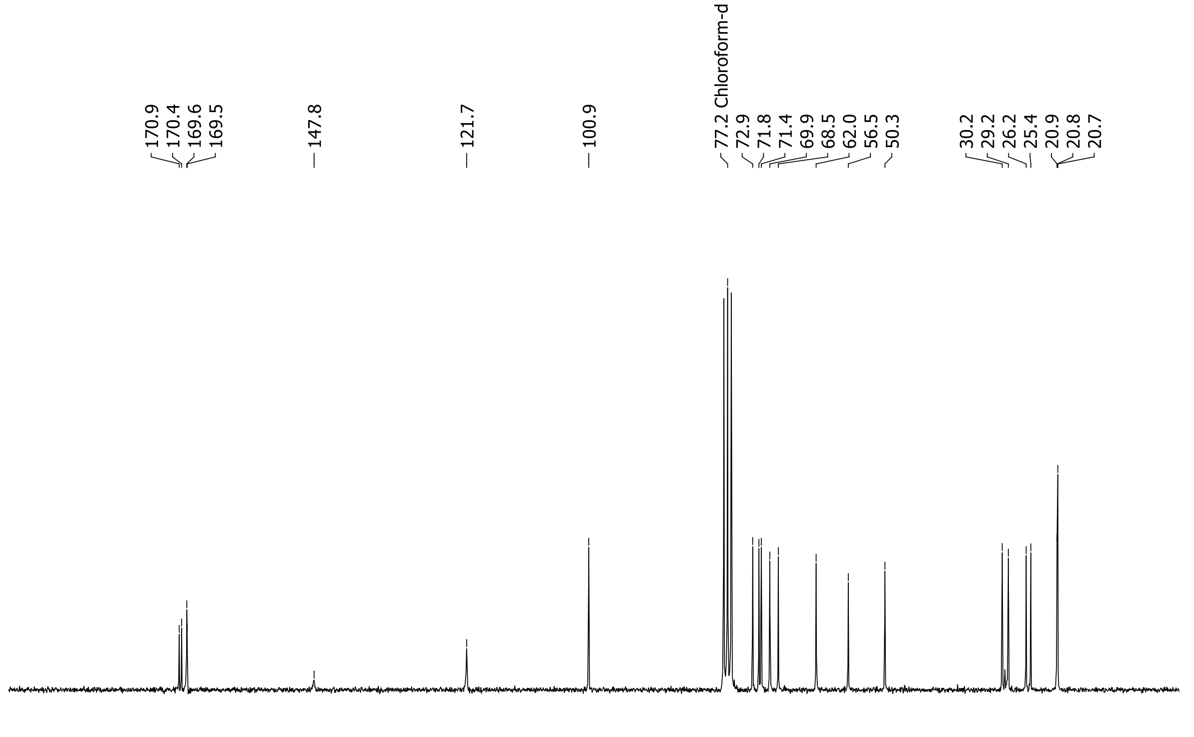

Figure S50: ^{13}C NMR spectrum of 48 (50 MHz, $CDCl_3$).

Stability assessment at pH* [15] 4.3 for 2-(nonyloxy)tetrahydro-2*H*-pyran acetal

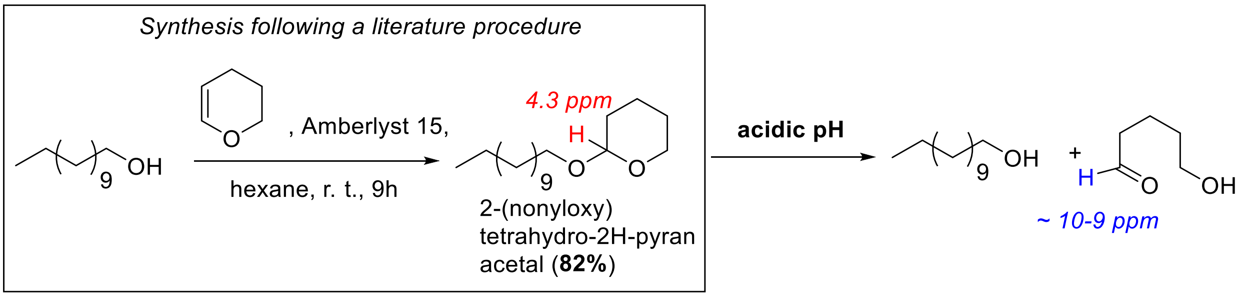

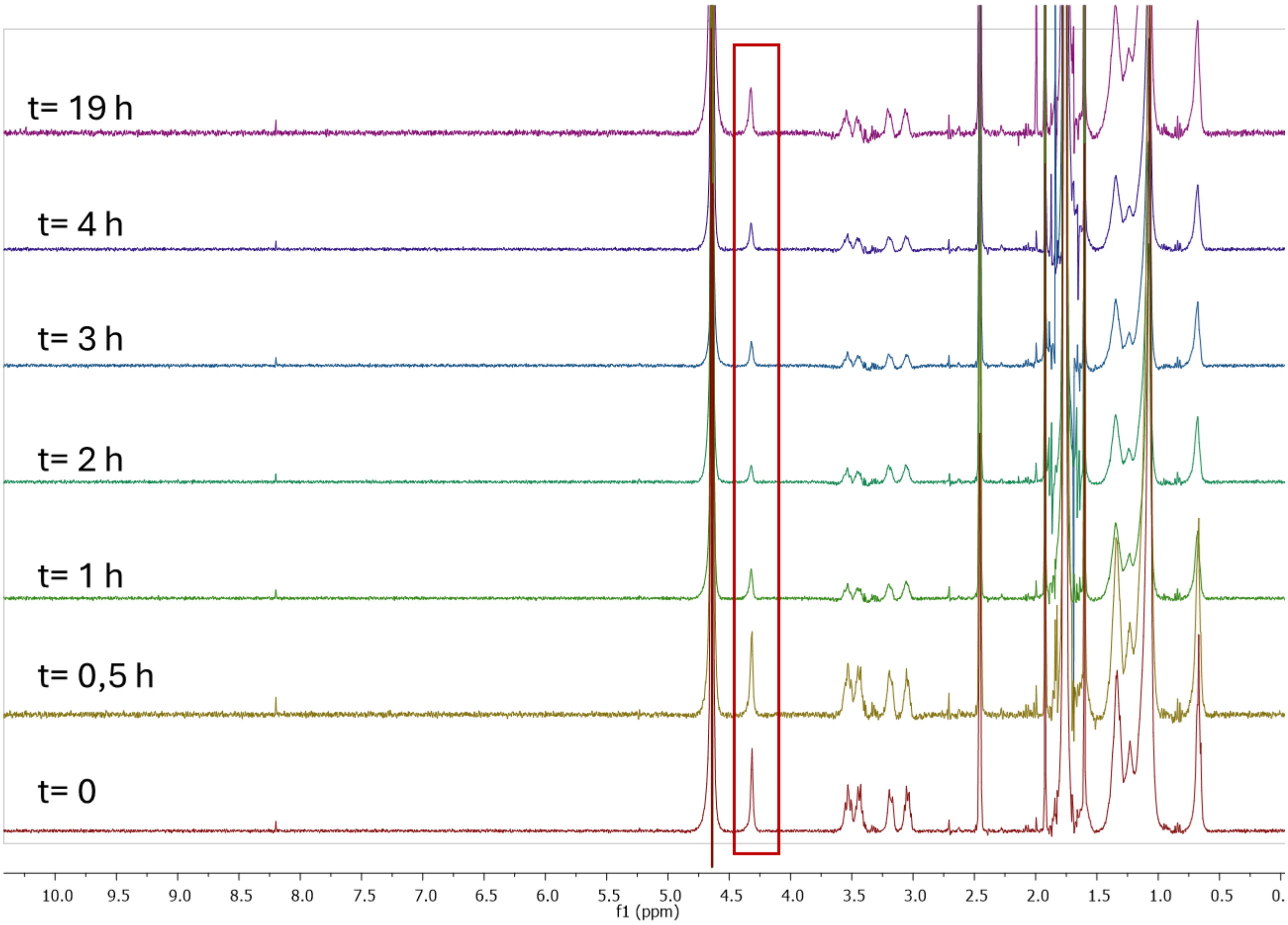

Figure S51. ^{1}H-NMR- spectra at 400 MHz in D_2O and Na-Acetate/acetic acid buffer at pH*4.3 and 37 °C. From bottom to top: spectra of 2-(nonyloxy)tetrahydro-2*H*-pyran acetal at different time points (t = 0 min, 0.5 h, 1 h, 2 h, 3 h, 4h, 19 h)

Enzymatic assays towards human GALNS in leukocytes isolated from healthy donors.

All experiments on biological materials were performed in accordance with the ethical standards of the institutional research committee and with the 1964 Helsinki Declaration and its later amendments. In keeping with ethical guidelines, all blood and cell samples were obtained for storage and analysed only after written informed consent of the patients (and/or their family members) was obtained, using a form approved by the local Ethics Committee (Assigned code: Lysolate "Late -onset Lysosomal Storage Disorders (LSDs) in the differential diagnosis of neurodegenerative diseases: development of new diagnostic procedures and focus on potential pharmacological chaperones (PCs)." Project ID code: 16774_bio, 5 May 2020, Comitato Etico Regionale per la Sperimentazione Clinica della Regione Toscana, Area Vasta Centro, Florence, Italy). Control and patient samples were anonymized and used only for research purposes.

A preliminary biological screening of compounds at 1 mM was performed towards GALNS in leukocytes isolated from healthy donors (controls). Isolated leukocytes were disrupted by sonication, and a micro BCA protein assay kit (Sigma–Aldrich) was used to determine the total protein amount for the enzymatic assay, according to the manufacturer instructions. Enzyme activity was measured by setting the reaction in 0.2 mL tubes and performing the experiments in triplicates as follows. Compound solution (3 µL), 4.29 µg/µL leukocyte homogenate 1:5 (7 µL) and 20 µL of 4-methylumbelliferyl-β-galactoside-6-sulfate.Na substrate solution (6.66mM) in Na-Acetate/acetic acid buffer (0.1 M/0.1 M, pH 4.3) containing 0.1 M NaCl, 0.02% (w/v) NaN3 and 5 mM Pb-acetate were incubated for 17 h at 37 °C. After step one, the tubes were placed on an ice cooler and the reaction was stopped by addition of 5 µL of Na-phosphate buffer (0.9 M, pH 4.3) containing 0.02 % (w/v) of NaN3 and by efficient mixing with vortex. Then, 10 µL of β-galactosidase from *Aspergillus oryzae* (β-Gal-A-10U) were added to each sample and samples were incubated for 2 h at 37°C. At the end of this period the tubes were placed on an ice cooler and the samples were transferred in a cooled flat–bottomed 96 well plate and the reaction was immediately stopped with 200 µL of $NaHCO_3/Na_2CO_3$ buffer (0.5 M/0.5 M pH 10.7) containing 0.025% (w/v) of Triton X-100. Fluorescence was measured in a SpectraMax M2 microplate reader (Molecular-Devices) using a 365 nm excitation wavelength and a 435 nm emission wavelength. Percentage of GALNS inhibition was given with respect to the control (without compound). Experiments were performed in triplicate, and the mean ± S. D. was calculated.

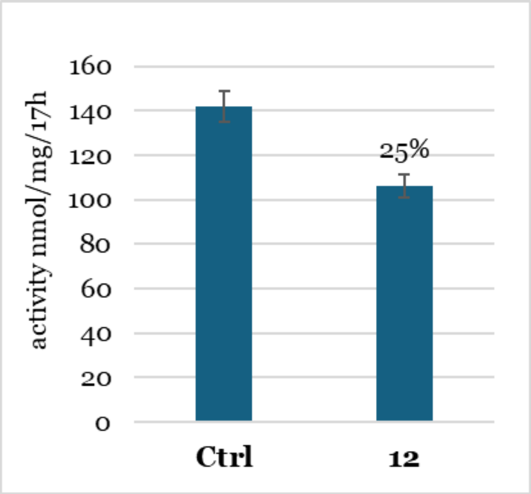

Figure S52. GALNS activity in the absence (Ctrl) and presence of scaffold **12** at a 1 mM concentration in human leukocyte extracts. The corresponding calculated inhibition percentages are shown above each bar.

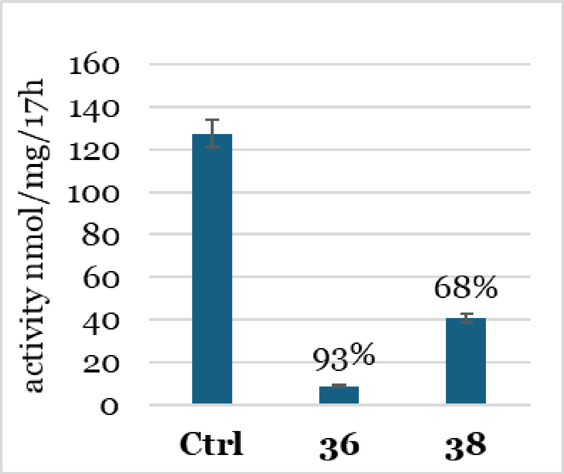

Figure S53. GALNS activity in the absence (Ctrl) and presence of the new multivalent compounds **36** and **38** (as reported in Table 1 of the main text) at a 1 mM concentration in human leukocyte extracts. The corresponding calculated inhibition percentages are shown above each bar.

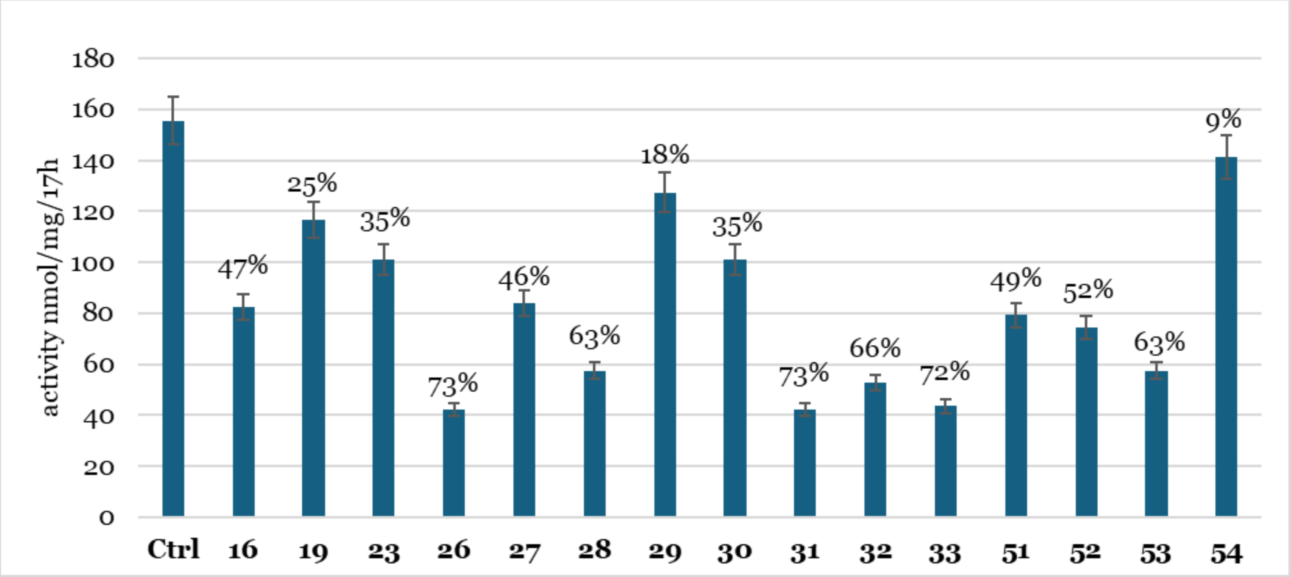

Figure S54 Activity of GALNS in the absence (Ctrl) and presence of multivalent pyrrolidines **16**, **19**, **23**, **26-29**, **30-33**, and **51** and of the multivalent pyrrolizidine compounds **52**, **54** and **53** (reported in Table 2 of main text) at 1 mM concentration in human leukocytes extracts. The corresponding calculated percentage of inhibition is indicated above each bar.

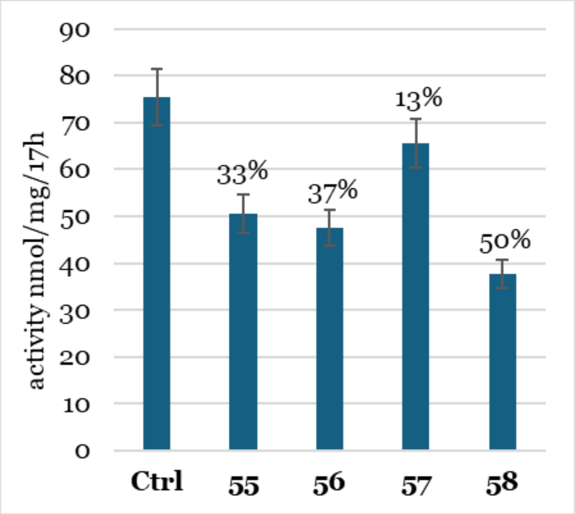

Figure S55. GALNS activity in the absence (Ctrl) and presence of monovalent piperidine **55** and multivalent piperidines **56-58** (as reported in Table 3 of the main text) at a 1 mM concentration in human leukocyte extracts. The corresponding calculated inhibition percentages are shown above each bar.

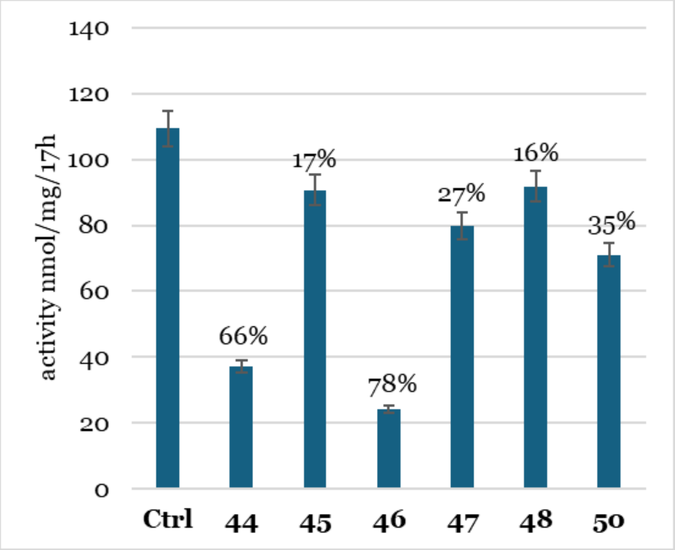

Figure S56. GALNS activity in the absence (Ctrl) and presence of monovalent sugar compound **48** and multivalent sugar compounds **44-47** and multivalent compound **50** (as reported in Table 4 of the main text) at a 1 mM concentration in human leukocyte extracts. The corresponding calculated inhibition percentages are shown above each bar.

IC_{50} determination

The IC_{50} values of inhibitors against human GALNS in leukocytes isolated from healthy donors or against recombinant human GALNS (rhGALNS VIMIZIM®) were determined by measuring the initial hydrolysis rate with 4-methylumbelliferyl-β-galactoside-6-sulfate.Na (6.66 mM) at different concentrations of inhibitors. Data obtained were fitted to the following equation using the OriginPro 2021 (Version 2021. OriginLab Corporation, Northampton, MA, USA):

$$\frac{Vi}{Vo} = \frac{Max - Min}{1 + \left(\frac{x}{IC_{50}}\right)^{slope}} + Min$$

where Vi/Vo, represent the ratio between the activity measured in the presence of the inhibitor (Vi) and the activity of the control without the inhibitor (Vo), "x" the inhibitor concentration, Max and Min, the maximal and minimal enzymatic activity observed, respectively.

IC_{50} in human GALNS in leukocytes isolated from healthy donors

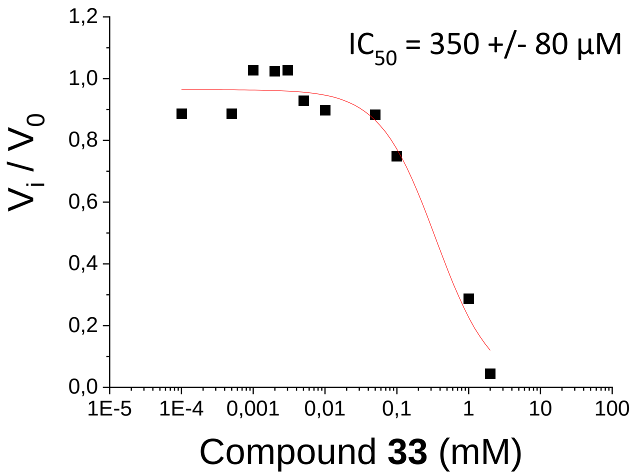

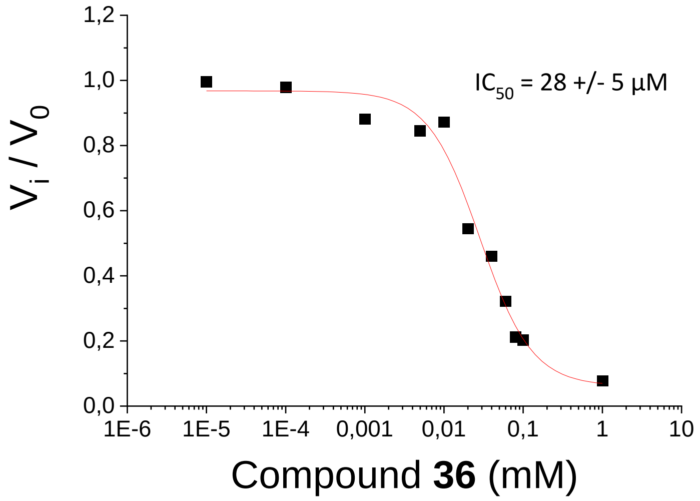

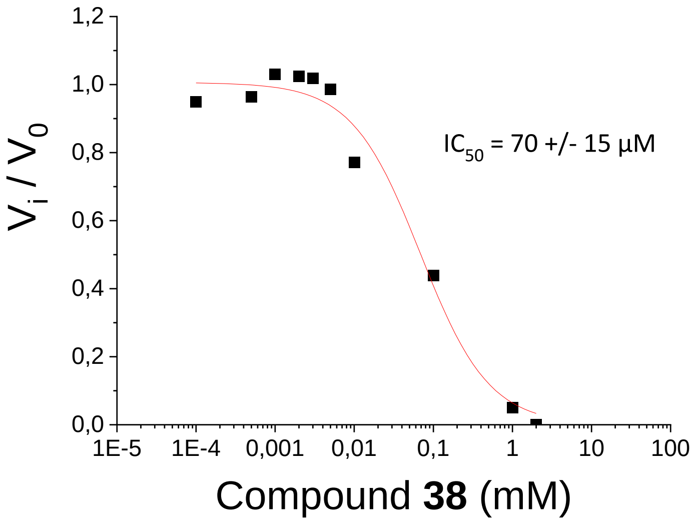

1,2
1,0
0,8
0,6
0,4
0,2
0,0
Vi / V0
IC50 = 70 +/- 15 μM
1E-5
1E-4
0,001
0,01
0,1
1
10
100
Compound 38 (mM)

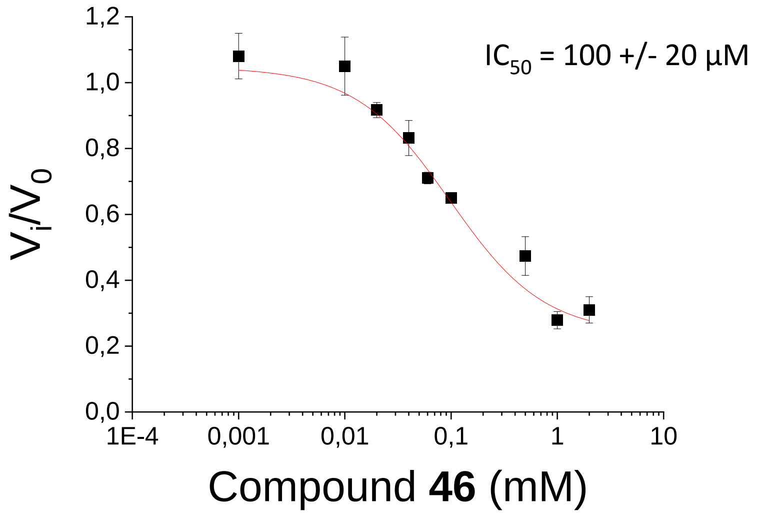

1,2
1,0
0,8
0,6
0,4
0,2
0,0
Vi/V0
IC50 = 100 +/- 20 μM
1E-4
0,001
0,01
0,1
1
10
Compound 46 (mM)

IC_{50} in recombinant human GALNS (rhGALNS VIMIZIM®)

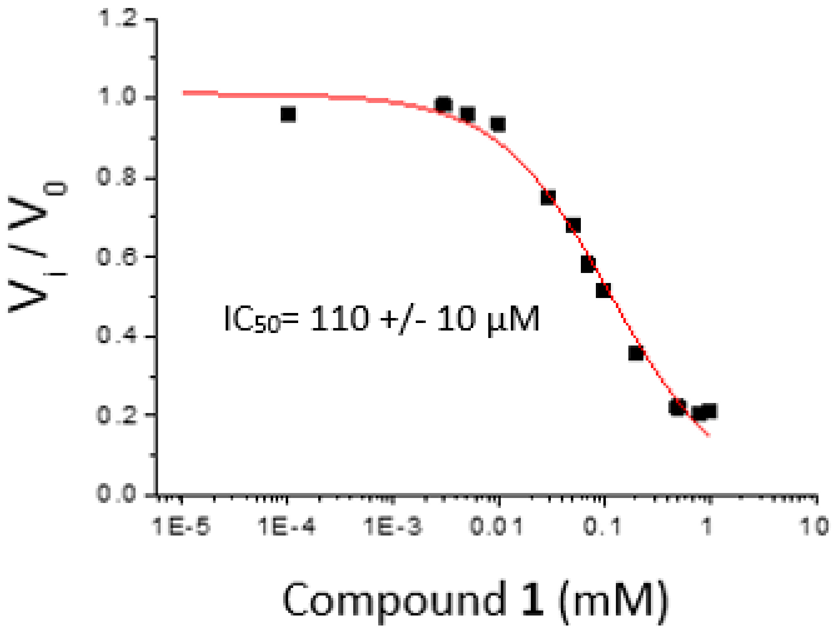

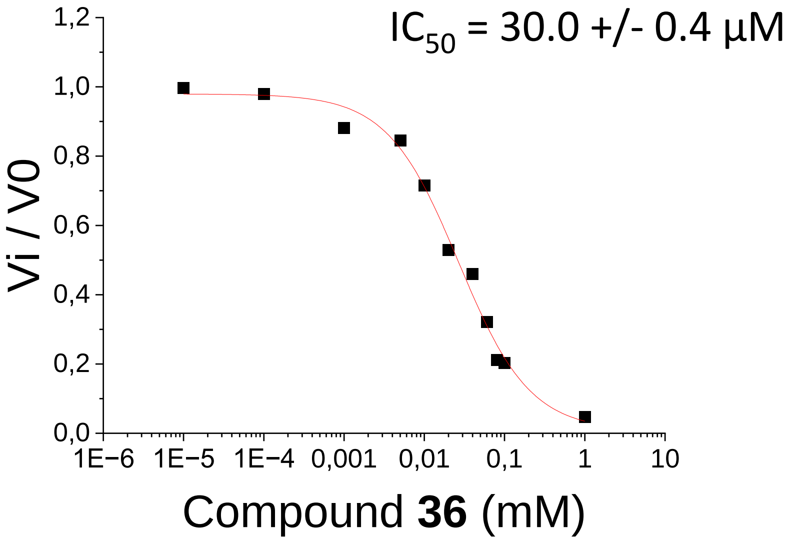

Stabilization of Recombinant GALNS under Thermal Denaturation Conditions [16].

The thermal stability induced by **1** and **36** against rhGALNS VIMIZIM® was determined by measuring the hydrolysis rate with 4-methylumbelliferyl-β-galactoside-6-sulfate·Na (6.66 mM) after denaturation of enzyme at 48 °C for 40 minutes or 60 minutes with or without (Ctrl) different concentrations of compounds.

Compound (3 µL) and rhGALNS (7 µL) were incubated for 10 minutes in an ice bath and then for 0 minutes or 40 minutes or 60 minutes at 48 °C.

After incubation 20 µL of 4-methylumbelliferyl-β-galactoside-6-sulfate·Na substrate solution in Na-Acetate/acetic acid buffer (0.1 M/0.1 M, pH 4.3) containing 0.1 M NaCl, 0.02% (w/v) NaN_3 and 5 mM Pb-acetate was added. The tubes were incubated for 17 h at 37 °C. After this first incubation the tubes were placed on an ice cooler and the reaction was stopped by addition of 5 µL of Na-phosphate buffer (0.9 M, pH 4.3) containing 0.02 % (w/v) of NaN_3 and by efficient mixing with vortex. Then, 10 µL of β-Gal-A-10U were added to each sample and samples were incubated for 2 h at 37°C. After this time, the tubes were placed on an ice cooler and the samples were transferred in a cooled flat–bottomed 96 well plate and the reaction was immediately stopped with 200 µL of $NaHCO_3/Na_2CO_3$ buffer (0.5 M/0.5 M pH 10.7) containing 0.025% (w/v) of Triton X-100. Fluorescence was measured in a SpectraMax M2 microplate reader (Molecular-Devices) using a 365 nm excitation wavelength and a 435 nm emission wavelength. Experiments were performed in triplicate, and the mean ± S. D. was calculated.

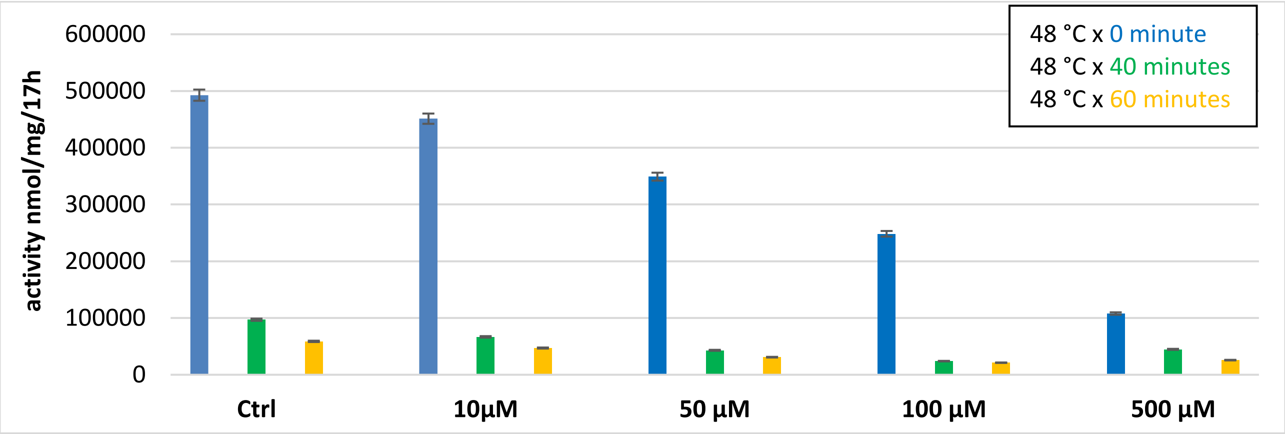

Figure S57. Activity of rhGALNS VIMIZIM® was determined after incubation for 0 minutes or 40 minutes or 60 minutes at 48 °C with or without (Ctrl) different concentrations of **1** by measuring the hydrolysis rate 4-methylumbelliferyl-β-galactoside-6-sulfate·Na.

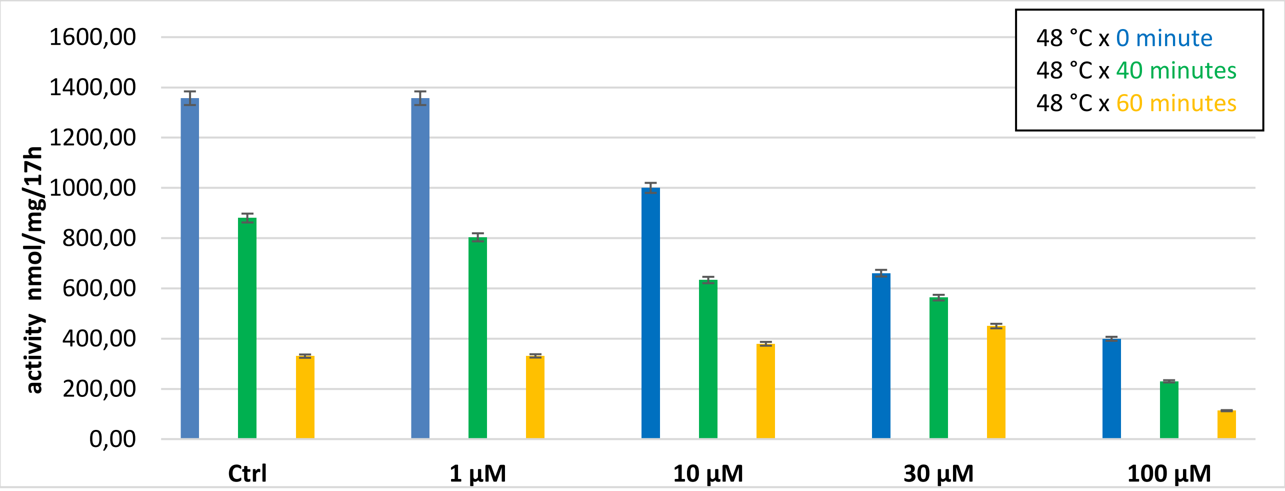

Figure S58. Activity of rhGALNS VIMIZIM® was determined after incubation for 0 minutes or 40 minutes or 60 minutes at 48 °C with or without (Ctrl) different concentrations of **36** by measuring the hydrolysis rate 4-methylumbelliferyl-β-galactoside-6-sulfate·Na.

References:

1. Gottlieb H. E.; Kotlyar V.; Nudelman A. J. NMR Chemical Shifts of Common Laboratory Solvents as Trace Impurities. *Org. Chem.* **1997**, *62*, 7512-7515.
2. Mirabella S.; D'Adamio G.; Matassini C.; Goti A.; Delgado S.; Gimeno A.; Robina I.; Moreno-Vargas A. J.; Šesták S.; Jiménez-Barbero J.; Cardona F. Mechanistic Insight into the Binding of Multivalent Pyrrolidines to α-Mannosidases. *Chem. Eur. J.* **2017**, *23*, 14585-14596.
3. Ferhati X.; Matassini C.; Fabbrini M. G.; Goti A.; Morrone A.; Cardona F.; Moreno-Vargas A. J.; Paoli P. Dual targeting of PTP1B and glucosidases with new bifunctional iminosugar inhibitors to address type 2 diabetes. *Bioorganic Chemistry* **2019**, *87*, 534–549.
4. Papp I.; Dernedde J.; Enders S.; Haag R. Modular synthesis of multivalent glycoarchitectures and their unique selectin binding behavior. *Chem. Commun.* **2008**, 5851–5853.
5. D'Adamio G.; Matassini C.; Parmeggiani C.; Catarzi S.; Morrone A.; Goti A.; Paoli P.; Cardona F. Evidence for a multivalent effect in inhibition of sulfatases involved in lysosomal storage disorders (LSDs). *RSC Adv.* **2016**, *6*, 64847–64851.
6. Mourer M.; Hapiot F.; Tilloy S.; Monflier E.; Menuel S. Easily Accessible Mono-and Polytopic β-Cyclodextrin Hosts by Click Chemistry. *Eur. J. Org. Chem.* **2008**, 5723–5730
7. Martínez-Bailén M.; Jiménez-Ortega E.; Carmona A. T.; Robina I.; Sanz-Aparicio J.; Talens-Perales D.; Polaina J.; Matassini C.; Cardona F.; Moreno-Vargas A. J. Structural basis of the inhibition of GH1 β-glucosidases by multivalent pyrrolidine iminosugars. *Bioorg. Chem.* **2019**, *89*, 103026.
8. Chabre Y. M.; Contino-Pépin C.; Placide V.; Shiao T. C.; Roy R. Expeditive Synthesis of Glycodendrimer Scaffolds Based on Versatile TRIS and Mannoside Derivatives. *J. Org. Chem.* **2008**, *73*, 5602–5605.
9. Segura M.; Sansone F.; Casnati A.; Ungaro R. Synthesis of Lower Rim Polyhydroxylated Calix[4]arenes. *Synthesis*, **2001**, 2105–2112.
10. Chabre Y. M.; Giguère D.; Blanchard B.; Rodrigue J.; Rocheleau S.; Neault M.; Rauthu S.; Papadopoulos A.; Arnold A. A.; Imberty A.; Roy R. Combining glycomimetic and multivalent strategies toward designing potent bacterial lectin inhibitors. *Chem. Eur. J.* **2011**, *17*, 6545-6562.
11. Michihata N.; Kaneko Y.; Kasai Y.; Tanigawa K.; Hirokane T.; Higasa S.; Yamada H. High-yield total synthesis of (-)-strictinin through intramolecular coupling of gallates. *J. Org. Chem.* **2013**, *78*, 4319–4328.
12. Miyagawa A.; Yamamura H. Synthesis of Branched Tetrasaccharide Derivatives of Schizophyllan-like β-Glucan. Carbohydr. Polym. **2020**, *227*, 115105-115113.
13. Van Scherpenzeel M.; Van den Berg R.; Donker-Koopman W. E.; Liskamp R. M. J.; Aerts J. M. F. G.; Overkleeft H. S.; Pieters R. Nanomolar affinity, iminosugar-based chemical probes for specific labeling of lysosomal glucocerebrosidase. *J. Bioorg. Med. Chem.* **2010**, *18*, 267–273.
14. Higa C. M.; Tek A. T.; Wojtecki R. J.; R. Braslau. Nonmigratory Internal Plasticization of Poly(Vinyl Chloride) via Pendant Triazoles Bearing Alkyl or Polyether Esters. *J. Polym. Sci., Part A:Polym. Chem.,* **2018**, *56*, 2397–2411.
15. Krężel, A.; Bal, W. A formula for correlating pKa values determined in D2O and H2O, *J. Inorg. Biochem.* **2004**, *98*, 161-166.
16. Buco F.; Matassini C.; Vanni C.; Clemente F.; Paoli P.; Carozzini C.; Beni A.; Cardona F.; Goti A.; Moya S. E.; Ortore M. G.; Andreozzi P.; Morrone A.; Marradi M. Gold nanoparticles decorated with monosaccharides and sulfated ligands as potential modulators of the lysosomal enzyme N-acetylgalactosamine-6-sulfatase (GALNS). *Org. Biomol. Chem.* **2023**, *21*, 9362-9371.
